# Recording animal-view videos of the natural world using a novel camera system and software package

**DOI:** 10.1371/journal.pbio.3002444

**Published:** 2024-01-23

**Authors:** Vera Vasas, Mark C. Lowell, Juliana Villa, Quentin D. Jamison, Anna G. Siegle, Pavan Kumar Reddy Katta, Pushyami Bhagavathula, Peter G. Kevan, Drew Fulton, Neil Losin, David Kepplinger, Michael K. Yetzbacher, Shakiba Salehian, Rebecca E. Forkner, Daniel Hanley

**Affiliations:** 1 School of Biological and Behavioural Sciences, Queen Mary University of London, London, United Kingdom; 2 Theorem Engine, Alexandria, Virginia, United States of America; 3 Department of Biology, George Mason University, Fairfax, Virginia, United States of America; 4 Department of Computer Science, George Mason University, Fairfax, Virginia, United States of America; 5 School of Environmental Sciences, University of Guelph, Guelph, Canada; 6 Drew Fulton Photography, Gainesville, Florida, United States of America; 7 Day’s Edge Productions, San Diego, California, United States of America; 8 Department of Statistics, George Mason University, Fairfax, Virginia, United States of America; 9 US Naval Research Laboratory, Washington, DC, United States of America; University of California Davis, UNITED STATES

## Abstract

Plants, animals, and fungi display a rich tapestry of colors. Animals, in particular, use colors in dynamic displays performed in spatially complex environments. Although current approaches for studying colors are objective and repeatable, they miss the temporal variation of color signals entirely. Here, we introduce hardware and software that provide ecologists and filmmakers the ability to accurately record animal-perceived colors in motion. Specifically, our Python codes transform photos or videos into perceivable units (quantum catches) for animals of known photoreceptor sensitivity. The plans and codes necessary for end-users to capture animal-view videos are all open source and publicly available to encourage continual community development. The camera system and the associated software package will allow ecologists to investigate how animals use colors in dynamic behavioral displays, the ways natural illumination alters perceived colors, and other questions that remained unaddressed until now due to a lack of suitable tools. Finally, it provides scientists and filmmakers with a new, empirically grounded approach for depicting the perceptual worlds of nonhuman animals.

## Abstract 2

Many animals use colors in dynamic displays performed in spatially complex environments, but what do the animals actually see? This study presents a newly developed camera system and video processing pipeline that accurately records animal-perceived colors in motion and in natural settings, allowing researchers to study color signals in their full complexity.

## Introduction

How do animals see the world? This simple question has captured our imaginations and spurred discovery since the advent of modern science [1]. Each animal possesses a unique set of photoreceptors, with sensitivities ranging from ultraviolet through infrared, adapted to their ecological needs [2]. In addition, many animals can detect polarized light [3–5]. As a result, each animal perceives color differently [6]. As neither our eyes nor commercial cameras capture such variation in light, wide swaths of visual domains remain unexplored. This makes false color imagery of animal vision powerful and compelling [7–9]. Unfortunately, current techniques are unable to quantify perceived colors of organisms in motion, even though such movement is often crucial for color appearance and signal detection [10,11]. Overcoming this serious barrier should spur widespread advancements in the field of sensory ecology [12,13]. Here, we provide a solution to these challenges by introducing a tool for researchers or filmmakers to record videos that represent the colors that animals see.

Accurately portraying animal-perceived colors requires careful consideration of the perceptual abilities of the relevant receivers [14–16]. Ultimately, visual stimulation depends on the illumination, the object’s reflectance, and the sensitivity of the receiver’s photoreceptors [17]. Traditional spectrophotometric approaches rely on using object-reflected light to estimate the responses of an animal’s photoreceptors [18,19]. Though accurate, individually measuring reflectance spectra for each object in a visual display is laborious and all spatial and temporal information is lost in the process. Moreover, measurements must be taken from sufficiently large, uniformly colored, relatively smooth and flat surfaces [20]. Unfortunately, many natural colors pose technical challenges. For example, powders and latticed materials, or objects that are iridescent, glossy, transparent, translucent, or luminescent, or animals that shift their colors using iridophores [2,4,5,21–26] are difficult or impossible to measure with spectrophotometry.

Multispectral photography provides the opportunity to accurately measure colored patterns in situ [27–30]. It relies on taking a series of photos in wavelength ranges that are generally broader than standard “human-visible” photographs. Typically, subjects are photographed using a camera sensitive to broadband light, through a succession of narrow-bandpass filters [8,31,32]. In this way, researchers acquire images in regions surpassing the human visual experience (e.g., the UV and infrared ranges), organize these as a stack of multiple, clearly differentiated color channels [27,31,33], from which they can derive camera-independent measurements of color [28,29,34]. These approaches have a rich tradition in the study of pollinator-plant relations, where the relationship between colorimetry and the visual systems of diverse organisms has long been embraced [8,9,31,32,35]. The resultant multispectral images trade some accuracy for tremendous improvements in spatial information, which is often a welcomed compromise when the goal is to understand animal signals. However, by its nature the method works only on still objects, and it alone is unsuitable for studying the temporal aspects of signals [10].

Yet, temporal changes can be central to a biological signal [10,11]. A recently proposed approach for studying dynamic color signals combines multispectral imaging with digital 3D modeling [36,37]. The next step will be to animate the resulting 3D multispectral models and further analyze them in silico, simulating a variety of receiver-specific visual models or viewing conditions [37–40]. Such models will likely play a crucial role in our understanding of the ways animal posture and the receivers’ viewpoints alter visual signals. It is both a strength and limitation that the approach offers full control of the simulated environment. While it will allow researchers to simulate colors viewed from different angles and in variable light environments, it does not aspire to collect these data under natural conditions. In such natural settings, animals present and perceive signals from complex shapes that cast shadows and generate highlights [11]. These signals vary under continuously changing illumination and vantage points, and organisms can position themselves purposefully in these settings. Information on this interplay among background, illumination, and dynamic signals is scarce. Yet, it forms a crucial aspect of the ways colors are used [12,13], and therefore, perceived by free-living organisms in natural settings.

In this paper, we take a different approach. We present a camera system and an associated computational framework that produces animal-view videos, with sufficient precision to be used for scientific purposes. This new tool captures the full complexity of visual signals, as perceived under natural contexts, where moving targets may be unevenly illuminated. The camera system records videos in 4 channels (UV, blue, green, and red) simultaneously. Data from this, or similar, systems can then be processed using a set of transformations that directly translates the video recordings to estimates of perceivable units (quantum catches) for animals of known photoreceptor sensitivity. Using this approach, it is now possible to record moving stimuli—crucially in perceptual units of animal viewers—and study the temporal components of visual signals.

## Results and discussion

**Fig 1:**
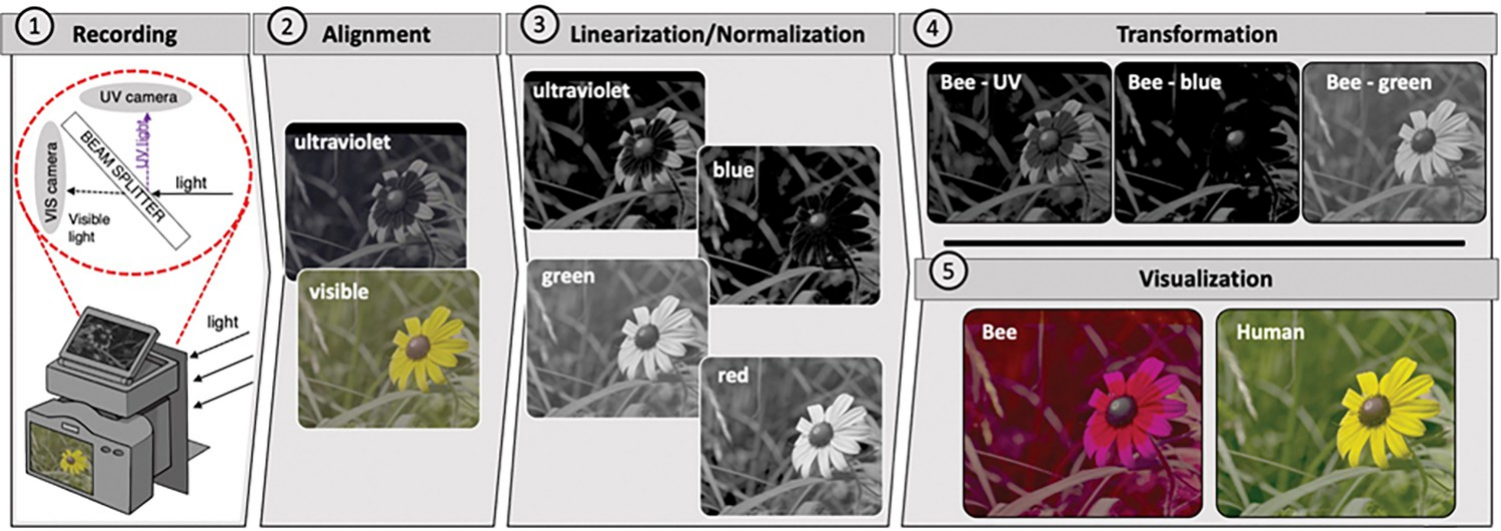
Recording and video processing pipeline. Scenes are (1) projected to an internal beam splitter that reflects UV light and passes visible light to 2 independent cameras. This design eliminates the need for switching filters and so allows for the rapid collection of multispectral recordings (videos or images). Following data collection, users can use our pipeline to (2) align the recordings automatically. The recordings are (3) linearized and normalized automatically using the custom color card or a set of grayscales of known reflectivity. This step estimates the light captured by the camera sensors (camera catches, CC). Finally, the camera catches are (4) transformed to animal quantum catches (AC, in this case representing honeybee *Apis mellifera* vision), which can subsequently be (5) visualized as false color images or videos (labeled as “bee”) by coloring the UV, blue, and green quantum catch images as blue, green, and red, respectively. These are compared to the composition of the linear images or videos (labeled as “human”). In this case, we demonstrate the pipeline using a black-eyed Susan *Rudbeckia hirta*. This flower has a nectar guide that aids recruitment [43]. To our eye, the black-eyed Susan appears entirely yellow because in the human-visible range, it reflects primarily long wavelength light. Whereas in the bee false color image, the distal petals appear magenta because they also reflect UV, stimulating both the UV-sensitive photoreceptors (depicted as blue) and those sensitive to green light (depicted as red). By contrast, the central portion of the petals does not reflect UV and therefore appears red. For more information on the color key, see S7 Fig. For the purpose of this illustration, we applied a gamma correction to the false color images (CC^0.5^ and AC^0.3^, respectively). For details on the pipeline, see Materials and methods. The resultant images in (5) are also available in a larger format with an embedded visual color key (S7 Fig).

**Fig 2:**
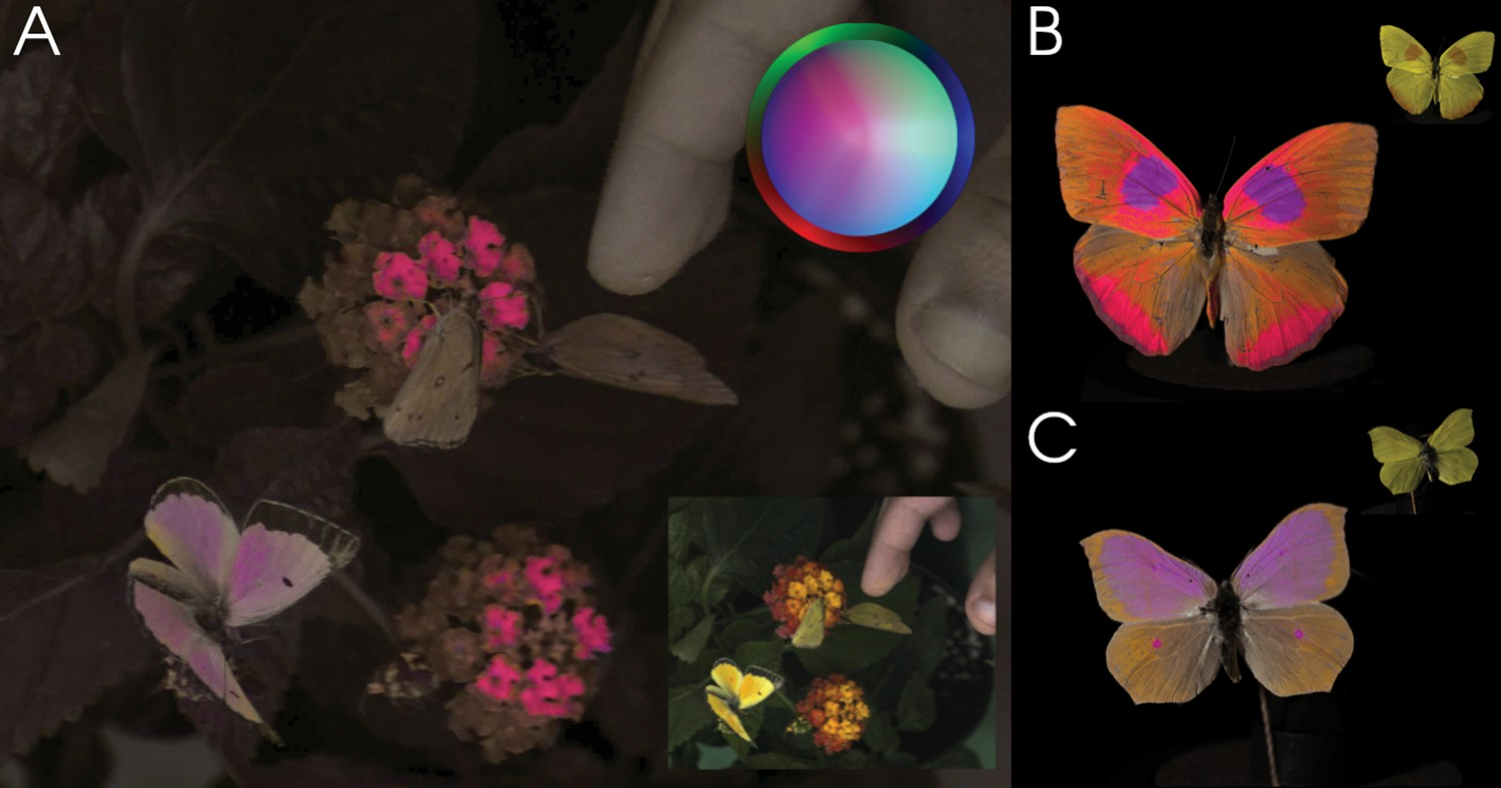
Frame excerpts from false color videos. Our pipeline can produce false color videos of animals behaving in their natural environment. (A) Here, we show 3 male orange sulphurs (*Colias eurytheme*). These butterflies display strong angle-dependent UV iridescence on the dorsal side of their wings. The UV-iridescent portions appear more orange to the human observer than the otherwise yellow wings (see human-visible inset). The avian false color depiction (main image) is based on the coordinates of the receptor noise-limited (RNL) opponent space [17,44]. Coordinates in this space represent differences in an animal’s photoreceptor responses, and the distances between the coordinates approximate the perceptual distances between the colors. The inset color key illustrates human-visible colors around the perimeter of a circle colored according to RNL coordinates, where UV colors start at the epicenter and mix with other colors. For full details, see Method A in S1 Text. In this depiction, the UV-iridescent portions appear purple, because they most strongly reflect in the ultraviolet and the red part of the spectrum. The ventral part of the wings, visible when the animals are in their resting position, are depicted as gray-brown, just like the leaves, as they are close to the achromatic point. See S1 Video. (B, C) A potentially useful application of the system is the fast digitization of museum specimens. Here, we highlight pigmentary and structural UV coloration on specimens of (B) *Phoebis philea* and (C) *Anteos* sp. in RNL false colors. A human-visible inset appears to the top right corner of each. The specimens are mounted on a slowly rotating stand, showcasing how the iridescent colors change depending on viewing angle (S2 and S3 Videos). The bright magenta colors highlight the strongly UV-reflective areas, while the purple areas reflect similar amounts of UV and long wavelength light. For the purpose of this illustration, we applied a gamma correction to the false color video of the (A) live butterflies; the source videos are also available. For further examples, see S4–S12 Videos.

Our pipeline was designed with utility in mind. It combines existing methods of multispectral photography with a newly developed hardware design and a set of transformation functions implemented in Python to record and process videos (Fig 1). The hardware includes a beam splitter that separates UV from visible light, and directs images from these respective wavebands to 2 independent consumer-level cameras. The crucial advantage of this approach is that all color channels are recorded simultaneously, which enables video recording. Recordings are then linearized and transformed into animal-perceived colors [27,28,33,41,42], in the form of photoreceptor quantum catches. The detection of custom color standards and the linearization and transformation processes are automated (see Materials and methods for details). The resulting animal-view videos can be further analyzed or combined as false color imagery (Fig 2) [9,29,31]. As an added benefit of our approach, we provide methods for quantifying estimation error within each experiment. Finally, our pipeline is very flexible, allowing the user to easily swap cameras, lenses, or to visualize color appearance for a variety of animal viewers. As all components are open source, the tools invite continued improvements (see Data availability).

**Fig 3:**
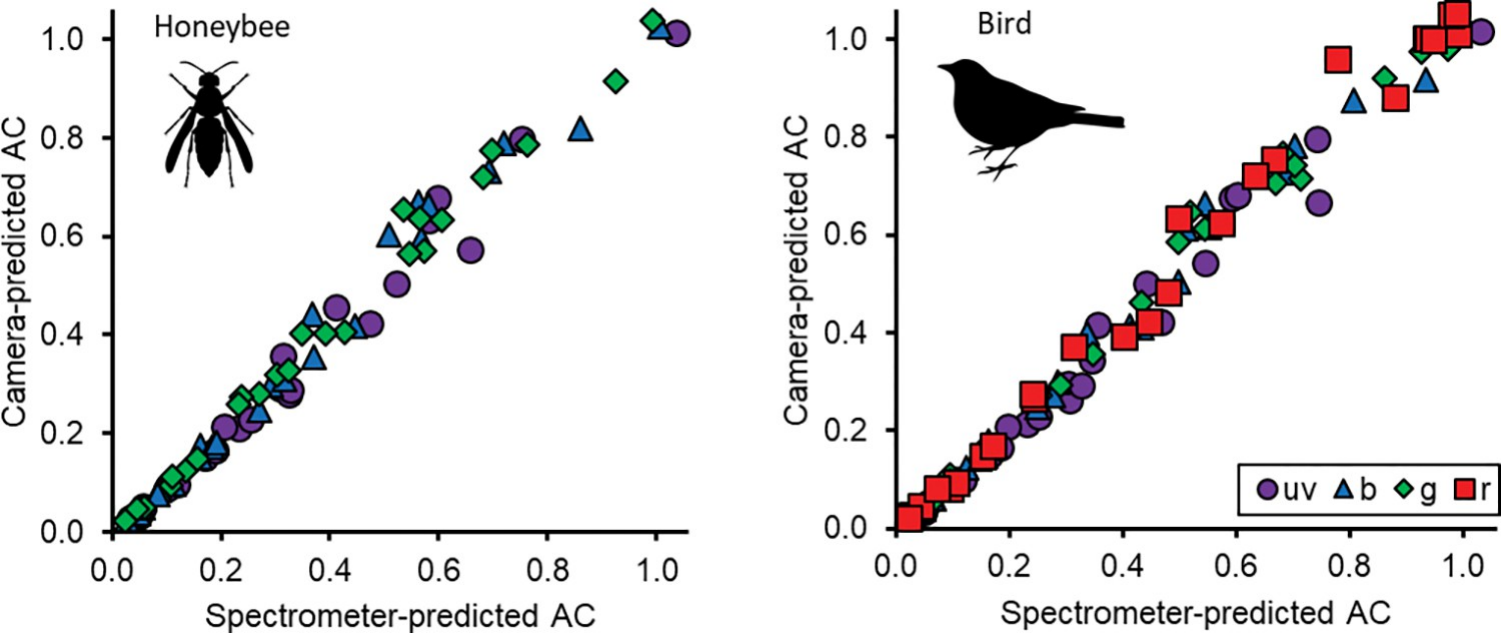
Quantum catch recovery from animal-view video. Video recordings can produce accurate estimates of animal quantum catches. In this case, we illustrate the accuracy by comparing animal quantum catches derived from our pipeline (“Camera-predicted AC”) to animal quantum catches derived from reflectance spectra (“Spectrometer-predicted AC”), for the honeybee (left) and average ultraviolet-sensitive bird (right). We present quantum catch estimates for photoreceptors broadly sensitive to ultraviolet (violet circles; R^2^_honeybee_ = 0.982, R^2^_bird_ = 0.981), blue (blue triangle; R^2^_honeybee_ = 0.985, R^2^_bird_ = 0.989), green (green diamonds; R^2^_honeybee_ = 0.990, R^2^_bird_ = 0.992), and red light (red squares; for the bird alone; R^2^_bird_ = 0.988). The camera-predicted colors were within 2 just noticeable differences from spectrometer-predicted colors (mean ± s.e. [max]: *bee* = 0.810 ± 0.089 [2.02] jnd; *bird*: 0.805 ± 0.082 [1.786] jnd). For a detailed description of the data, see S1 Fig and S1 Table; for further tests see S2–S6 Figs and S2–S7 Tables. The inset bee (“Hymenoptera icon” by Shyamal L.) and bird (“bird” by SVG SILH) silhouettes were released under Creative Commons CC0 license. The data underlying this figure can be found in S1 Data.

This approach accurately produces photoreceptor quantum catches for honeybees (*Apis mellifera*) or the average UV-sensitive avian viewer [45] and it can work for any organism provided users supply data on photoreceptor sensitivity and those sensitivities overlap with the sensitivity of the camera system. We tested the accuracy of the pipeline on a collection of color standards, by comparing the estimates of the camera system to those predicted from spectrophotometry (see Materials and methods for details; Figs 3 and S1–S6 and S1–S6 Tables, including for other species: S7 Table). Under ideal conditions, i.e., under stable, direct sunlight, the coefficients of determination (R^2^) of the predictions are in the range of 0.962 < R^2^ < 0.992 (S1–S3 Figs and S1–S3 Tables). Recording in the lab, under a broadband lamp whose emission spectrum has multiple peaks, is less reliable, but still produces fits in the range of 0.928 < R^2^ < 0.966 (S4 and S5 Figs and S4 and S5 Tables). Shaded conditions outside have subtle spatial and temporal variation in light that are imperceptible for the human eye; on videos where the calibration and the test frames were separated by several seconds, this noise lowers the coefficients of determination to 0.928 < R^2^ < 0.965 (S6 Fig and S6 Table). The technique is not a substitute for a true hyperspectral camera and relies on the fact that the reflectance of most natural materials is sufficiently nonrandom, and the photoreceptor sensitivity of most animals is sufficiently broadband that a statistical relationship between camera catches and animal quantum catches holds. By exploiting this relationship, the technique reliably predicts animal quantum catches derived from spectrophotometry, under either artificial illumination indoors or natural sunlight outdoors, which highlights the broader applicability of our method.

Animal-view videos provide a new possibility for researchers to study spatially and temporally complex, multimodal displays, in nature where these signals are produced and perceived (Fig 2 and S1–S3 Videos). We highlight 3 such frontiers. First, as the method does not require switching parts (e.g., filters or lenses) between taking UV and visible images, which is common in other applications of multispectral photography, it allows for more rapid image collection (S2–S4 Videos). Therefore, this approach can speed up data acquisition in the pipelines of color estimation and photogrammetry projects [37,40]. Second, signals and displays can be studied in their natural contexts. For example, in natural settings the intensity and spectral distribution of light continuously shifts and some habitats can have very patchy illumination (e.g., dappled light in forests). In addition, the incidence angle of directional light sources, such as sunlight, will alter how animals perceive objects (see micaToolbox User Guide, [28]). This effect is particularly evident on glossy or iridescent surfaces (e.g., some feathers, S4 Video), but ultimately applies to all natural surfaces. As a result, the perceived color of a leaf fluttering in the wind or a bird walking in the undergrowth will constantly change. Our approach provides a simple pipeline to capture and analyze colorful signals as they would be perceived in the environment where they are produced and experienced by free-living organisms [45,46]. Finally, and perhaps most importantly, it is now possible to study the temporal variation of perceived colors using videography. By analyzing animal-view videos of the natural world, researchers can now explore a wide range of cues and signals in motion (S1–S12 Videos). The camera system and associated software package introduced here will open many new research avenues for sensory ecology.

## Materials and methods

### Camera system

**Fig 4:**
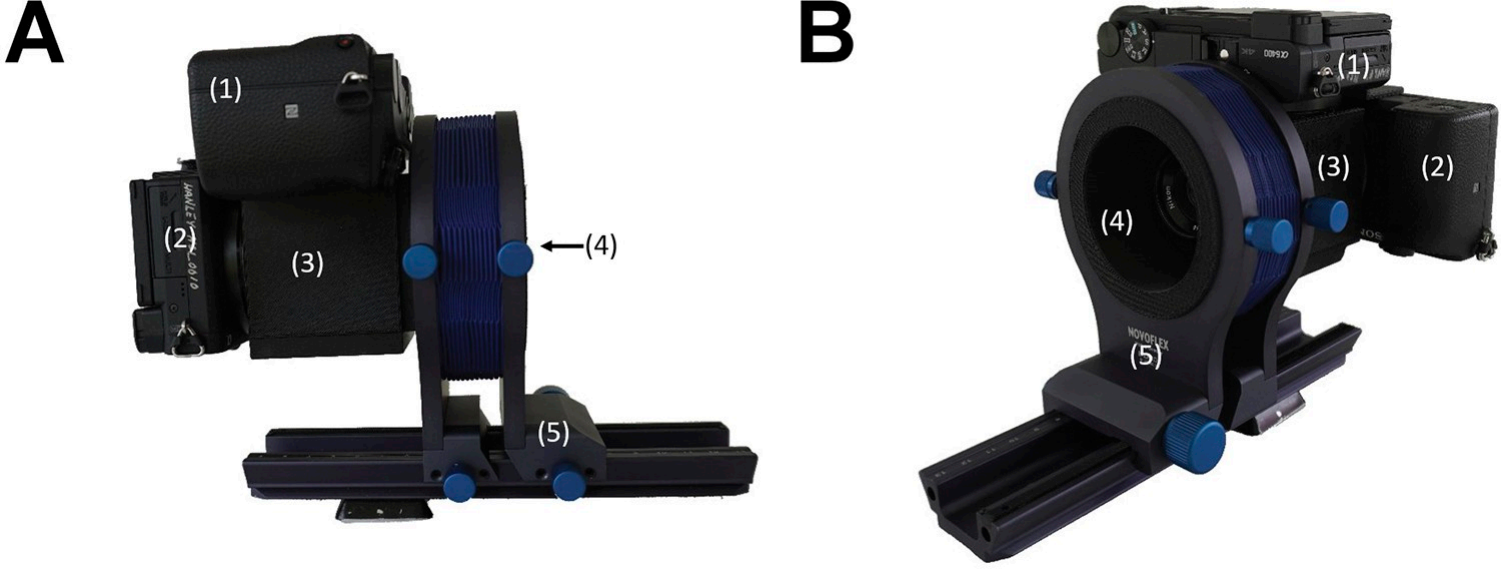
Illustration of the camera system. Views of the camera system from the (A) side and a (B) ¾ perspective illustrate the system’s 2 cameras that are sensitive to (1) UV and (2) visible light, the (3) modular cage, and the (4) enlarging lens within a recessed (see arrow in A) custom mount. Here, we illustrate the camera system on the commercially available (5) Novoflex BALPRO bellows system, which facilitates focusing and mounting of alternative lenses. For plans and further details, please see Data availability.

**Fig 5:**
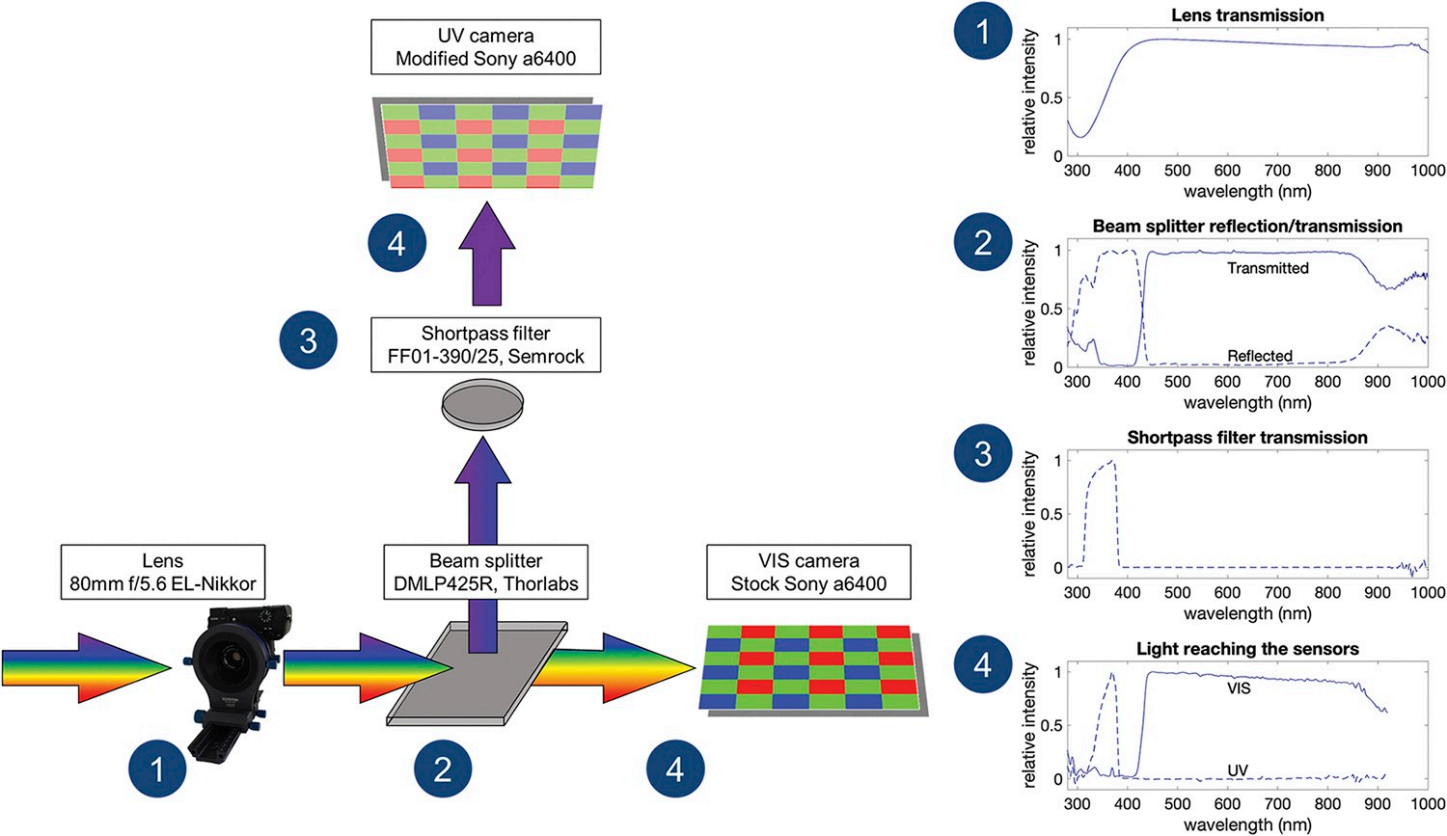
The spectral properties of the optical components. A single camera lens (1) passes light to a dichroic beam splitter that reflects short wavelength (<425 nm) and transmits visible (>425 nm) light. (2) The reflected short wavelength light passes through a shortpass filter (3) before reaching a full-spectrum camera, while the transmitted visible light is captured by a stock camera. This setup ensures that only UV and VIS light reaches the respective cameras (4). The reflection/transmission curves are given for each leg of the optical path in relative units (they are normalized to the range of 0–1). VIS, visible; UV, ultraviolet. For the technical details on the transmission measurements, see Method B in S1 Text. The data underlying this figure can be found in S1 Data.

The camera system is built from commercially available parts and cameras (Sony a6400) fixed in a modular 3D printed housing (Fig 4). The housing (S8 and S9 Figs) consists of a modular cage, mounts for the beam splitter mirror and the shortpass filter, cone baffles that minimize light leakage towards the cameras, and a bellows lens mount. We printed the housing with a commercially available 3D printer (Prusa, Prusa i3 MKS+) from matte black PLA filament (Hatchbox, PLA black) to further minimize possible light contamination (for more details, see Data availability). In this manuscript, we used a single camera lens (80 mm f/5.6 EL-Nikkor enlarger lens); however, we provide designs to accommodate 3 different commercially available lenses (80 mm f/5.6, 135 mm f/5.6, or 210 mm f/5.6 Nikon lenses from the EL-Nikkor Enlarging lenses series). Light that transmits through the lens is passed to a dichroic beam splitter (DMLP425R, Thorlabs), placed at a 45° angle relative to the beam path (Fig 5). This beam splitter reflects short wavelength light (<425 nm), which, after passing through a shortpass filter (FF01-390/25, Semrock) that only transmits short wavelength light (<390 nm), reaches a full-spectrum camera (UV camera, Fig 5). Our full-spectrum camera was modified by Lifepixel (https://www.lifepixel.com/) by replacing the stock internal hot-mirror (that normally blocks light outside of the visible range) with a full-spectrum glass filter; a range of vendors offer this service [47]. Simultaneously, visible light (approximately 425 nm to 720 nm) passes through the beam splitter, to be captured by a stock camera at the rear of the housing (VIS camera, Fig 5). In our build, both cameras are triggered simultaneously using a spliced cable release; however, one could use a remote trigger or a trigger delay (setting the start of the recording several seconds after pressing the shutter button). In the camera system’s simplest form, the lens can be fixed directly to the housing, which results in a fixed focal plane. However, the design allows for other options. For example, we used a Novoflex BALPRO bellows to allow for dynamic focusing (from approximately 64 mm up to infinity; Fig 4) and provide plans for DIY bellows with a sliding rail (S9 Fig). Alternatively, if space allows, a focusing helicoid can be attached to the front lens mounting plate. The enlarger lenses are nearly apochromatic (i.e., having the ability to focus all wavelengths at the same point); however, the focus will inevitably differ slightly between cameras receiving UV and visible light. To overcome this, we designed 2 unique camera mount plates that offset each camera a specific distance from the beam splitter (UV 32 mm; VIS 30 mm).

The system is designed to be flexible. The modular design allows users to devise new mounting plates, typically just a single side of the cube-shaped housing, to accommodate different lenses, cameras, or even internal optical components (e.g., beam splitter or shortpass filter). For example, while we have built our system around capturing the UV and human-visible portions of the light spectrum, this system could easily be adapted to capture infrared by swapping out the filter, beam splitter, and (possibly) the lens. If the system is modified for recording polarized light, care must be taken to employ a beam splitter whose reflection and transmission properties are not sensitive to the polarization plane of the light (for details, see S10 Fig). All 3D printed parts of this build are available for download, along with detailed instructions for printing and assembly (see Data availability).

### Estimating sensor sensitivity

**Fig 6:**
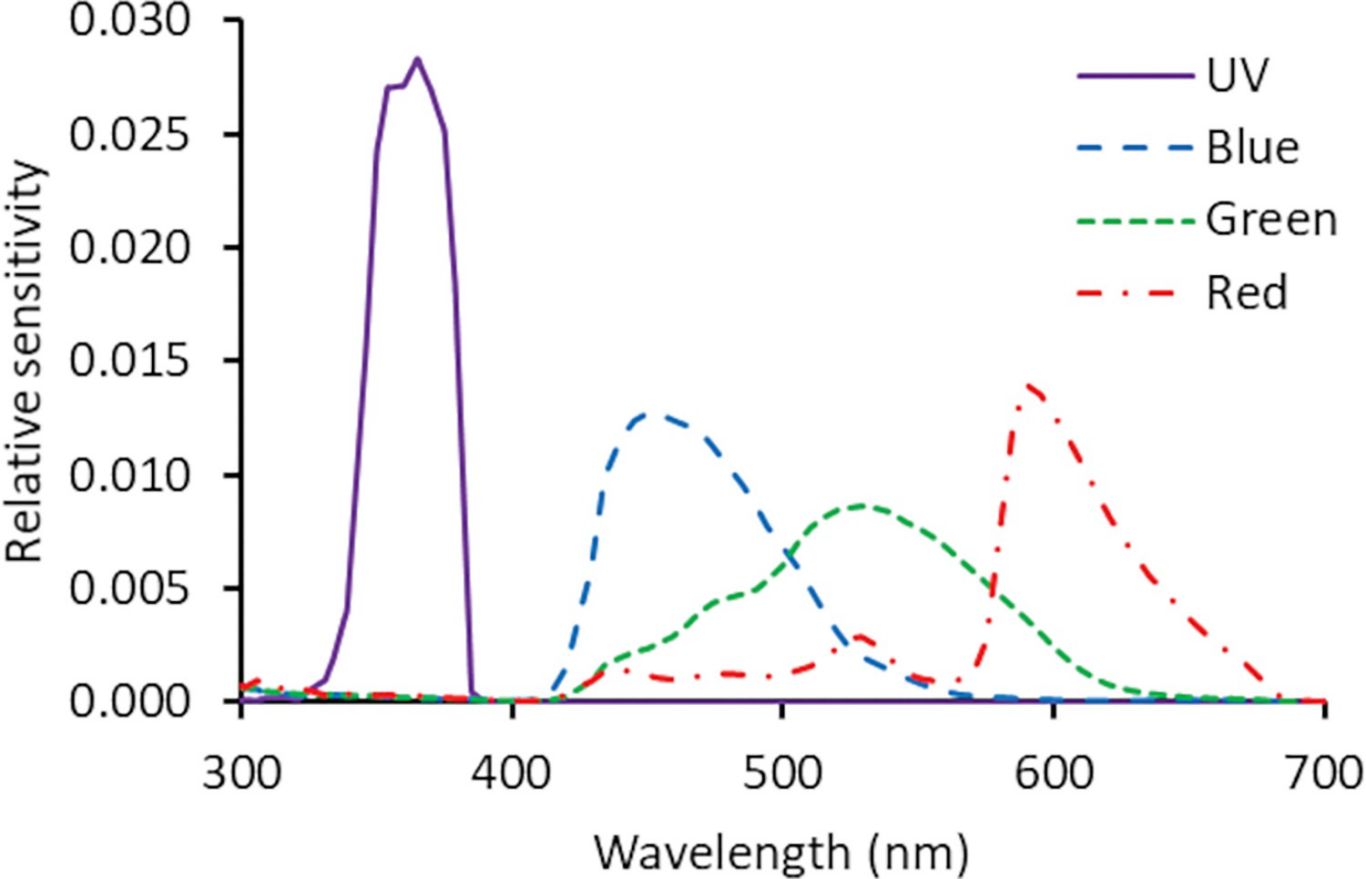
Camera sensor sensitivity. Estimates of relative camera sensor sensitivity for the UV (solid purple lines), blue (dashed blue lines), green (dotted green lines), and red (dash-dotted red lines) sensors from 300 nm to 700 nm; see Materials and methods. These sensitivities closely resembled those remeasured over a broader range 280 nm to 1,100 nm (for more details, see S12 Fig, Method B in S1 Text). The data underlying this figure can be found in S1 Data.

We used a monochromator to measure the sensitivity of the camera sensors from 280 nm to 800 nm [47–50]. Specifically, we connected a xenon light source (SLS 205, Thorlabs) to a monochromator (Optimetrics, DMC1-03) via a 1,000 μm single fiber (58458, Edmund Optics). The light from the monochromator was passed through a second 1,000 μm single fiber (58458, Edmund Optics) and collimating lens (Ocean Optics, 74-ACH) to a radiometrically calibrated spectrometer (Ocean Optics, Jaz) equipped with a cosine corrector made of Spectralon (CC-3-DA cosine). We incrementally varied light from 280 nm to 800 nm in approximately 5 nm steps in relatively narrow bands (mean FWHM ± s.e. = 7.3 ± 0.29 nm, S11 Fig). At each increment, we measured the absolute irradiance at a consistent distance and orientation and simultaneously photographed the illuminated cosine corrector in RAW (ARW) format with our camera system. The visible camera’s ISO was set to 100 and the UV camera’s ISO was set to 2,000. We calculated sensitivity from the photos and the absolute irradiance measurements as

$$S(\lambda )=\frac{P_{\lambda }-P_{d}}{\sum_{k=\lambda -20}^{\lambda +20}I(k)},$$ (1)

where S is the sensor sensitivity, *P*_*λ*_ is the pixel value for each measurement area (i.e., the area that is illuminated), *P*_*d*_ is the sensor value for the image from an equivalent sized dark portion of the frame (i.e., dark signal), and *I* is the irradiance of the light projected on the radiometer converted to photon flux (μmol s^−1^ m^−2^). In this case, we used the sum photon flux around the main peak to reduce the impact of second order scattering, which was detected at wavelengths ≥721 nm (second order peaks at ≥360.5 nm). We collected a second set of measurements on the UV camera where we inserted a longpass filter (transmission >415 nm, MidOpt LP415/25) within the beam path to block second order scatter. This reduced the pixel values of the UV sensor from 39.3 ± 2.7 to 1.7 ± 0.05, confirming that our sensitivity estimates were not influenced by second order scatter and that the UV camera was insensitive to longer wavelength light (mean ± s.e. difference from dark pixel = 0.14 ± 0.05, in pixel values in the 0–255 range). Finally, each sensor was relativized by dividing the estimated sensitivity at each wavelength by the total (sum) sensitivity, such that the sum of the resultant sensor sensitivity was equal to one for each sensor (Fig 6).

To ensure that our system was insensitive to infrared light, we remeasured the sensor sensitivities from 280 nm to 1,100 nm using a similar approach (Method B in S1 Text). This showed no measurable sensitivity to light above 800 nm and produced nearly identical sensitivity estimates (S12 Fig). Note that at impractically long exposure times the UV camera is minimally sensitive to infrared light (as is the case for multispectral photography in general); however, this has no measurable impact on data collection (see S12 and S13 Figs). When photographing in full sunlight, where the surface power density of infrared irradiance is 5 to 6 times higher than that of UV, at the shutter speeds required for UV photography, the infrared contamination is statistically indistinguishable from dark noise (comparison of pixel values under no light versus under infrared light, Z-test, *UV*: *p* = 0.743; *blue*: *p* = 0.960; *green*: *p* = 0.841; *red*: *p* = 0.985; for more details, see S13 Fig). However, if users wish to record in a light environment with a greater relative proportion of infrared to UV irradiance, we recommend exercising caution and quantifying the possible infrared contamination (S13 Fig).

Systems built from different cameras will have different sensor sensitivities than ours, and users will need to estimate the responses of their own systems. If the equipment we listed above (monochromator, spectrophotometer, stable full-spectrum light source) is not available, the camera sensitivities can be estimated using chart-based methods (also see online documentation for the micaToolbox, [28,42]).

### Shooting images and videos with the camera system

It is important to note that the angles of the camera, subject, and illuminant will have notable effects on colors [51]. Because our goal is to capture colors as perceived, the camera needs to be positioned at the vantage point of the relevant animal receiver (e.g., overhead or from ground level, depending on the target species). Our current design requires manual focusing, so keeping fast-moving subjects within the focus plane can be challenging. Therefore, initial investigations should focus on subjects that reliably signal within predictable locations (see examples on S1–S12 Videos).

Both cameras should be set to record using an S-Log3 gamma correction, which applies a specific gamma function that reduces the likelihood of under- or overexposure, and in the S-Gamut3 color space, which corresponds to camera native color space. As a result, the S-Log3/S-Gamut3 format provides greater access to the camera’s full dynamic range than other color spaces, which facilitates color grading. The RGB values recorded in this way can be used to accurately reconstruct the camera’s RAW sensor responses from both JPG and MP4 files [52]. Sony provides a transformation function to convert S-Log3 files to native “reflectance” [52], but these are also recoverable using a power function (see Linearization and normalization below, [27,33]). The S-Log3 gamma correction and the S-Gamut3 color space offer a particularly useful format because pixel values recorded this way depend only on their respective RAW values. This is in contrast with typical gamma corrections and color spaces, which employ additional camera-capture-to-RGB mappings, intended to suit human perception. Such mappings depend on additional color matrices that introduce co-dependence among the color channels to transform colors, in ways which often constitute undisclosed proprietary information.

Once in position, the camera’s shutter speed can be adjusted such that a set of isoluminant grayscale (white to black) standards is properly exposed. We recommend a custom grayscale made from mixing barium sulfate paint (Labsphere 6080) with a spectrally flat black paint (Culture Hustle, Black 3.0), which is reliable and comparatively inexpensive. Alternatively, a commercially available set of 8 calibrated Spectralon standards reflecting 99, 80, 60, 40, 20, 10, 5, and 2% of incident light (Labsphere, RSS-08-020) produces excellent results. In this study, we used both custom and Spectralon grayscales and confirmed that they work equally well. The calibration needs to be repeated for each session as it is specific to the light conditions during recording. When collecting images, the calibration shots should be taken at least once for a photo session, but taking calibration shots periodically or including the standards in each image is ideal. When recording videos, the calibration frames should be included in the video. However, just as with other approaches [27,28], the calibration can also be completed just after photographing or filming a behavioral display as long as the light conditions have not changed during the session.

We provide methods to validate the accuracy of the transformation (see Transformation and Validation tests below). These tests require colors that have substantial variation in reflectance over a broad spectral range encompassing the visual sensitivity of the target organism (e.g., 300 nm to 700 nm). As in other studies [28], we used commercially available pastels (Blick Art Supplies, 20 Artists’ Pastels Half Sticks. 21948–1209) alongside the previously described custom grayscale made from a barium sulfate and flat black paint (S14 Fig and S8 Table). These color and grayscale standards were held within a 3D printable holder made from gray PLA plastic (we used Prusament silver PLA and also a darker, Overture black PLA). We placed 4 Augmented Reality University of Cordoba (ARUCO) fiducial markers [53] in the corners of the 3D printable holder. These ARUCO markers are used for automatic linearization and validation tests (see below). The color standards can be challenging to produce, thus we encourage great care in preparing the reference colors. We found that applying the pastels on a vertical surface while vacuuming helps minimize the potential for colored dust to contaminate adjacent colors. For the grayscales, finding adequate mixtures can take some time; however, the precise reflectance spectra do not matter as much as maximizing the coverage of the grayscale range. Finally, these color cards need to be remade and remeasured approximately every 3 months as they can degrade with age.

### Alignment

An initial step of the pipeline involves a two-stage process of aligning the recordings of the VIS and UV cameras. First, we apply a coarse alignment to the UV channel using a homography warp (Method C in S1 Text). Establishing the parameters of this initial coarse alignment requires the user to manually select suitable matching points from the UV and VIS recordings, but the result can be reused indefinitely because the cameras’ relative position remains approximately constant. Although our modular system holds both cameras securely, very slight shifts between sessions are inevitable. In addition, the affine transforms may slightly differ depending on the target’s distance to the camera. For these reasons, as a second step we calculate a fine-grained correction to the coarse alignment. We use the enhanced correlation coefficient (ECC) algorithm to find this correction (see Method C in S1 Text for a description, [54]). Since this algorithm can sometimes fail when image conditions are poor, we calculate several candidate alignments using different image pairs. The number of pairs used is determined by how many images can be stored in memory at a time. We then apply each candidate alignment to a large batch of image pairs and evaluate them using the mean ECC value across each image pair, selecting the alignment with the best average ECC. For videos, the frames also need to be aligned temporally, i.e., the UV channel needs to be shifted so that the frames from the 2 cameras correspond with each other. The temporal alignment algorithm first identifies a batch of frames for which there is adequate motion. For this batch, we calculate the spatial alignment for a range of temporal offsets, then select the best offset by calculating the mean ECC value just as we do when selecting the optimal spatial alignment, only this time finding both spatial and temporal matches simultaneously.

Perfect temporal synchronization is not possible since the 2 cameras operate independently. Therefore, the best temporal alignment can be up to half a frame length misaligned. The optimal exposure of the UV camera, typically 1/30, limits the videos’ frame rate to 30 fps. Accordingly, the best temporal alignments can range from a perfect match up to 1/60 of a second off. Animals moving faster than this rate will produce “ghosting” in addition to the more familiar blurring. In practice, video alignment works well for the majority of cases and we provide a simple method for detecting alignment failures. Specifically, we present the user with a composite image whose red and blue bands are taken from the visible light camera and whose green band is taken from the aligned UV camera. If spatial or temporal alignment has failed, this will be readily apparent as “ghosting” in the composite image, allowing for manual correction if necessary.

### Linearization and normalization

Many digital cameras do not directly output RAW video recordings or these are impractical for applications in the field. Rather, sensor responses are typically subjected to a variety of conversions, such as gamma correction and white balancing, to produce images optimized for a human viewer [33]. Therefore, a necessary prerequisite for converting recordings to animal photoreceptor quantum catches is to estimate the relative responses of the camera system’s sensors (hereafter, camera catches), and to normalize them to a specified range [27,33].

First, the images need to be linearized. If using Sony cameras, both should be set to record using S-Log3 and the S-Gamut3 color space (see above). This setting ensures that the recording is made in a color space with a known relationship to the camera’s native color space, rather than being mapped into a standard color space such as sRGB or adobeRGB. Consequently, RGB values recorded in the S-Log3 color space closely correspond with the camera’s RAW sensor responses for both JPG and MP4 files and the linear sensor responses can be estimated with the transformation functions provided by Sony [52]. The specified relationship between the JPG pixel values and the RAW pixel values is given by:

$$\begin{array}{c} r=c_{1}c_{2}^{x_{\mathrm{JPG}}}+c_{3}\;for\;x_{\mathrm{JPG}}\geq c_{4}\\ r=c_{5}x_{\mathrm{JPG}}+c_{6}\;for\;x_{\mathrm{JPG}}<c_{4}, \end{array}$$ (2)

where *x*_JPG_ is the value of the pixel in the JPG scaled to the range [0, 1], *r* is the reflectance assuming isoluminance, and *c*_1_ = 0.0047058, *c*_2_ = 8166.69, *c*_3_ = −0.01, *c*_4_ = 0.1673609, *c*_5_ = 0.1914153077, *c*_6_ = −0.014023696 are constants. In practice, we found that applying an additional scaling term to the JPG pixel values prior to applying the Sony formula improved the error:

$$\begin{array}{c} r=c_{1}c_{2}^{sx_{\mathrm{JPG}}}+c_{3}\;for\;sx_{\mathrm{JPG}}\geq c_{4}\\ r=c_{5}sx_{\mathrm{JPG}}+c_{6}\;for\;sx_{\mathrm{JPG}}<c_{4}, \end{array}$$ (3)

where the scaling term for the input is *s* = 0.92578125. Using this formula, a “roundtrip” converting from RAW to JPG and back to RAW had a similar mean absolute error to the base conversion from JPG to RAW, implying that this formula reconstructs the original RAW with an accuracy close to the best theoretically possible accuracy given the lossy JPG compression and 8-bit quantization (see Method D in S1 Text for details).

After linearizing, the images need to be normalized [27,28]. This step ensures that a value that is equally reflective over the camera’s full range (e.g., an isoluminant white standard) will register the same values across all of the camera’s sensors. The relationship is specific to the circumstances of the recording; therefore, the normalization parameters need to be estimated for each recording session separately and the illumination and camera settings should be maintained while recording images or videos.

The recorded pixel values should refer to a set of isoluminant standards with known reflectance curves. In this study, we used a custom grayscale (S14 Fig and S8 Table) made from mixing barium sulfate paint (Labsphere 6080) with a spectrally flat black paint (Culture Hustle, Black 3.0) and a commercially available set of 8 calibrated Spectralon standards reflecting 99, 80, 60, 40, 20, 10, 5, and 2% of incident light (Labsphere, RSS-08-020). To reduce the time required for manually selecting calibration and test samples within the images, we provide an automated process that reads the 4 ARUCO fiducial markers [53] on our custom color standard and extracts the pixel values of the color patches. The ARUCO algorithm provides a fast, highly accurate, automated method to locate relevant points on the custom color card.

We calculate the ideal linearized values by predicting the expected camera catch under ideal light conditions for a set of isoluminant standards. Specifically, we calculate the camera catch that would be expected under ideal (isoluminant) illumination for each channel as:

$$CC_{i}=\int_{300}^{700}R_{S}(\lambda )S_{i}(\lambda )I(\lambda )d\lambda ,$$ (4)

where *CC*_*i*_ is the camera catch of the sensor *i*, *R*_*s*_ is the spectral reflectance function of the stimulus, *S*_*i*_ is the spectral sensitivity function of the sensor, and *I* is the photon flux of the illuminant, in this case set to 1.0 across all wavelengths, for each wavelength λ. Having established the target values in this way, we then fit a linear regression between the original pixel values and the target values. These regressions describe the relationships between the recorded pixel values and the target camera catch for each sensor (S15 Fig). We use these linearization and normalization parameters to transform all observed pixel values into relative linearized camera responses (relativized such that an object with 100% reflectance would have a value of 1.0).

Although this method works for cameras that support the S-Log3 format (e.g., all R^2^ values ≅ 1.00, S15 Fig), in practice we found that we could simultaneously linearize and normalize by fitting a power law relationship between the JPG values and the target values on the samples using the trust region reflective algorithm [55]. The power law regression has the form:

$$CC_{i}=a_{1}a_{2}^{p_{i}}+a_{3},$$ (5)

where *p*_*i*_ is the pixel value of the channel *i* and *a*_1_, *a*_2_, *a*_3_ are parameters fit to the data. We found that there was very little difference in error between linearizing using Eq 3 and normalizing using linear regression versus linearizing and normalizing simultaneously using Eq 5 (S15 Fig), which implies that this method is extendable to other cameras as long as they have conventions similar to Sony (Method D in S1 Text).

This step of linearization and normalization removes the effects of unequal illumination from calibration images or frames, provided that the illumination is intense enough across the entire spectrum [27]; however, all other frames will be with reference to these calibration frames, even if there are subsequent shifts in illumination. Therefore, these steps should be repeated whenever the illumination changes. Just like other applications of multispectral imagery [27,28], we recommended placing standards in each frame, or recording standards immediately before or after a photo or video session. Once this step is completed, the camera catches from all 4 color channels are effectively linearized and normalized and ready to be transformed to animal quantum catches.

### Transformation

Natural spectra take on a limited variety of shapes [56]. This regularity enables the mapping of camera responses (camera catches) to photoreceptor quantum catches, which would otherwise be impossible. Thus, we can empirically derive a transformation matrix that reliably transforms from the camera catch to animal quantum catch [27,33,41,42]. This transformation matrix is specific to the camera’s sensors, the target animal’s photoreceptor sensitivities, and to the illumination under which the animal photoreceptor responses are to be estimated. However, it does not depend on the recording session [27].

When estimating a transformation matrix, first we calculate the camera catches (*CC*_*i*_, see above) for each object from a large database of spectral reflectance data. In our case, the sensitivity of the UV camera’s red, green, and blue bands was highly overlapping. To avoid overfitting the transformation functions, we rely only on the UV camera’s red channel; other users may use all 3 channels. We then calculate the expected photoreceptor quantum catch of the target animal using the equation:

$$AC_{j}=\int_{300}^{700}R_{S}(\lambda )S_{j}(\lambda )I(\lambda )d\lambda ,$$ (6)

where *AC*_*j*_ is the quantum catch of the photoreceptor *j*, *R*_*s*_ is the spectral reflectance function of the stimulus, *S*_*j*_ is the spectral sensitivity function of the receptor *j*, and *I* is the photon flux of the illuminant, for each wavelength λ. For simplicity, we assume ideal isoluminance between 300 nm and 700 nm. This corresponds to a scenario where there is sufficient light across the viewer animal’s full visual range (i.e., spanning the range of their photoreceptors’ sensitivity) and where the organism possesses perfect color constancy (i.e., the ability to perform color discrimination similarly despite differences in the light environment, [1]). However, it is possible to use any target illumination (e.g., S16 Fig). In this manuscript, we focus on spectral sensitivities of 2 example animals (S17 Fig): the honeybee *Apis mellifera* [57] and the average UV-sensitive avian viewer [45], using photoreceptor sensitivities downloaded from pavo [58]. As with the illumination, it is possible to enter user-defined spectral sensitivities and we have provided a small collection of spectral sensitivities from other organisms (see Data availability). From these 2 estimates (*CC*_*i*_ and *AC*_*j*_), we derive a transformation matrix, *T*, that maps the camera sensor space into animal receptor space:

AC=TCC, (7)

where *AC* is the vector of animal receptor responses and *CC* is the vector of camera catches. The entries of *T* were estimated by fitting linear models (without intercept) for each of the *j* photoreceptors such that:

$$AC_{j}\approx t_{j1}CC_{1}+t_{j2}CC_{2}+\cdots +t_{ji}CC_{i}.$$ (8)

In this paper, we used a large database of natural Floral Reflectance Database (FReD, consisting of 2,494 spectra, [59]) to fit the transformation matrix *T*. We randomly split the dataset into 2,244 spectra used for fitting *T* and 250 spectra used for evaluating the fit. We report the accuracy of the transformation step for a collection of example animals (including the honeybee and the average UV-sensitive avian viewer), as well as for arbitrary photoreceptor sensitivity curves based on the A1 and A2 templates from Govardovskii and colleagues [60]. To confirm that the results will generalize to other materials, we also tested the fit on a library of natural and artificial materials (Spectral Library of the USGS, [61]).

The conversion step is highly accurate. The coefficient of determination invariably exceeds 0.90, and in most cases performs above 0.99, for all example animals (S18 Fig and S9 Table). The results are generalizable to a set of viewing illuminations (S10 and S11 Tables). Our alternative reflectance library provided similar results, with slightly higher coefficients of determination (all >0.991, S12 and S13 Tables). We found reliable results for A1 and A2 synthetic photoreceptors with peaks between 343 nm and 700 nm (S19 Fig), for a variety of target illuminations (S20 Fig) and when tested on either database (S19 Fig). In the >420 nm range that is fully covered by the sensors of the visible camera, the conversion is very precise (R^2^ > 0.99). The system loses accuracy when extrapolating beyond the camera sensors’ sensitivity, i.e., when predicting the responses of receptors that are sensitive in the extreme end of UV (<340 nm) and becomes inaccurate for receptors that peak below 343 nm (S19 and S20 Figs). In addition, there is a small dip in accuracy at approximately 390 nm, which corresponds with a narrow (33 nm) gap in the cameras’ sensitivities (Fig 6). In particular, accuracy is lower when estimating photoreceptors with peak sensitivities of approximately 390 nm (S19 and S20 Fig) or when measuring materials whose reflectance is restricted to this region (S21 Fig and Method E in S1 Text). Importantly, the pipeline offers users the option to specify the spectral sensitivities of their study organism and the desired target illumination, and evaluate the fit of the transformation matrix on a case-by-case basis.

### Evaluating the success of the system

To ground-truth our method, we compared animal photoreceptor quantum catches calculated directly from reflectance spectra to those derived using our camera system and transformation functions. All measurements were diffuse spectral reflectance, measured using a field-portable spectrophotometer (Jaz, Ocean Optics) and pulsed xenon (Jaz-PX, Ocean Optics) light source. Each spectrum was relative to a fresh 99% Spectralon white standard (WS-1-SL, Ocean Optics) and a dark spectrum taken inside a custom black box. In this case, light was delivered through a 600 μm bi-furcating fiber optic positioned at a coincident oblique measurement angle.

The photoreceptor quantum catches estimated using the 2 methods match well. First, we tested our method on a selection of color standards: an ARUCO standard made from 20 pastels (S14 Fig and S8 Table) and a custom grayscale made from barium sulfate paint and black 3.0 (see above for details), another set of pastels (S22 Fig and S8 Table) and a DKK Color Calibration Chart. The tests were run on different images/frames than the calibrations, typically taken a minute after the frame/image where the calibrations were conducted. We repeated the validation tests for stills and videos, under natural, direct and indirect sunlight and in the lab under metal halide illumination (315W, iGrowtek MH315/10K; S16 Fig), for the 2 target animals: honeybees (*Apis mellifera*, [57]) and the average UV-sensitive avian viewer [45]. In all cases, we found good agreement between the reflectance-based and the camera-based estimations (S1–S6 Figs and S1–S6 Tables, 0.981 < R^2^ < 0.992 for videos in direct sunlight; 0.928 < R^2^ < 0.965 for videos in indirect sunlight; 0.962 < R^2^ < 0.985 for images taken in direct sunlight; 0.928 < R^2^ < 0.966 for images taken in the lab). Tests on different animals yielded comparable results (S7 Table, 0.941 < R^2^ < 0.980). In addition, we verified that the use of Spectralon standards and a custom-made grayscale provides similar results (S2 Table versus S3 Table and S4 Table versus S5 Table, linearized on Spectralon 0.942 < R^2^ < 0.985, linearized on ARUCO 0.928 < R^2^ < 0.983, depending on the channel and the lighting).

Next, we assessed the correspondence between spectrometer- and camera-predicted colors of natural objects. Specifically, we conducted the same tests as above on photos of flowers, leaves, birds’ eggs, and feathers (S14 Table). Again, for both target animals, photography and spectroscopy provided similar estimates of quantum catches (S23 Fig and S15 Table, 0.826 < R^2^ < 0.940). As expected, the linear relationship between the 2 estimates was weaker for natural objects than for color standards [62,63]. However, this mismatch represents meaningful variation: the perceived color of these objects is altered by their shape, fine patterning, texture, and physical color (e.g., Figs 2 and S24).

While these levels of accuracy are similar to applications of still multispectral photography on artificial objects [28,62,64] and natural specimens of known reflectance [62,63], we expect that our method trades some accuracy for the ability to register signals that would otherwise be impossible to measure (S25 Fig and S5–S8 Videos). For colorimetric studies where a still multispectral image is sufficient, already existing, highly accurate methods that use a single camera are likely optimal [28]. In other cases, the ability to record perceived colors from a moving organism is well worth a potential reduction in accuracy. Recognizing this, we designed our pipeline to allow users to test the accuracy of their color estimations, which will enable researchers to make informed decisions about the reliability of their datasets and the most appropriate methods for their particular use cases.

We want to stress that the coefficient of determination, R^2^, reported here and in comparable studies (e.g., [28,62–64]), must be interpreted with caution. This metric only quantifies the strength of the linear relationship between the expected and predicted quantum catches. Strong relationships that fall on a different slope than 1:1 may still have relatively high R^2^ values; therefore, these metrics may not be sufficient to interpret the accuracy of the predicted animal quantum catch. Thus, we also report mean absolute prediction error (MAPE), root mean squared prediction error (RMSPE), as well as the range of the inner 75% of the ordered prediction errors (S1–S7 Tables). These 3 measures are on the same scale as the expected quantum catches and allow for direct assessment of how well the system can predict animal quantum catch. Overall, considering these metrics alongside the coefficient of determination are more informative and we encourage users of multispectral photography to consistently assess and report their accuracy.

### Software

The software system was designed with end-users in mind, and it uses automation and interactive platforms where possible. The functionality is packaged as a Python library called video2vision and can be downloaded and installed from the PyPI repository or from GitHub (see Data availability). Operations are grouped into directed acyclic graphs which we call pipelines, essentially a flowchart of operations with no loops (implemented with NetworkX library, [65]). Pipelines are saved as JSON files for reuse. Still images or video frames are loaded as numpy arrays [66] and processed in batches to minimize runtime. Most image processing operations are performed using the OpenCV library [67]. The system can be called from the command line to process one or more images or videos, but there are a few components that require greater interactivity, such as checking for alignment errors or specifying the spatial relationship between ARUCO markers and sample points. These more interactive steps are implemented in Jupyter notebooks [68]. The software system is designed to be easily extendable; new image processing operations can be quickly added as new classes following a defined API.

### Ethics statement

Abandoned eggs were collected for another project under US Fish and Wildlife Service Collecting permit (MB81216C-3) and Virginia Department of Game and Inland Fisheries Scientific collecting permit (070605).

### List of supplementary videos

*All videos have been subjected to a histogram stretch and a subsequent gamma correction for the purposes of displaying on screen. The uncorrected videos, representing the quantum catches, are available from* https://doi.org/10.5281/zenodo.10145358.

## Supporting information

S1 Video Three male orange sulphur butterflies (*Colias eurytheme*) in avian RNL false colors. The dorsal side of the butterflies’ wings shows strong angle-dependent UV iridescence. The UV iridescence is sex-linked: the males display it as an important mating cue [69]. The UV-iridescent portions are hidden when the butterflies are in resting position, becoming visible during flight and when the butterfly is displaying. The avian false color depiction is based on the coordinates of the receptor noise-limited (RNL) opponent space. Coordinates in this space represent differences in an animal’s photoreceptor responses, and the distances between the coordinates approximate the perceptual distances between the colors; for full details see Method A in S1 Text. In this depiction, the UV-iridescent portions appear purple, because they most strongly reflect in the ultraviolet and the red part of the spectrum. The ventral part of the wings appears gray-brown, just like the leaves, indicating its color is closer to the achromatic point.
(MP4)

S2 Video Museum specimen of a *Phoebis philea* butterfly in avian RNL false colors. Another potentially useful application of the system is the fast digitization of museum specimens. This butterfly possesses both pigmentary and structural UV coloration. Bright magenta colors highlight the predominantly UV-reflective areas, while the areas appearing purple reflect similar amounts of UV and long wavelength light. The specimen is mounted on a stand and slowly rotated, showcasing how the iridescent colors change depending on viewing angle.
(MP4)

S3 Video Museum specimen of an *Anteos* sp. butterfly in avian RNL false colors. This butterfly displays angle-dependent UV iridescence on its wings, which appears as purple in this video. The specimen is mounted on a stand and slowly rotated, showcasing how the iridescent colors change depending on viewing angle. Quantifying iridescence in museum specimens is another potentially useful application of the system.
(MP4)

S4 Video Iridescent peacock feather through the eyes of 4 different animals. The camera system can measure angle-dependent structural colors such as iridescence. This is illustrated here through a video of a highly iridescent peacock (*Pavo cristatus*) feather. The colors in this video represent (A) peafowl *Pavo cristatus* false color, where blue, green, and red quantum catches are depicted as blue, green, and red, respectively, and the UV is overlaid as magenta. Although broadly similar to a standard color video, the UV-iridescence (annotated in the video at approximately 5 s) can be seen on the blue-green barbs of the ocellus (“eyespot”). Further UV iridescence can be seen along the perimeter of the ocellus (between the outer 2 green stripes). Interestingly, the iridescence is more notable to the peafowl than to (B) humans (standard colors), (C) honeybees, or (D) dogs. For a description of bee false colors, please see S7 Fig. False colors for dichromatic dogs were defined so that the blue and green channels correspond to the responses of the blue and green receptors, and the red channel to the average of the 2 receptors. In this way, the colors shift from purple to yellowish green, with an achromatic middle point where both receptors are equally excited. To demonstrate the visualization options, we also encoded the peacock feather in the RNL opponent space (starting at 19 s).
(MP4)

S5 Video A butterfly in *Apis* vision. We illustrate a zebra swallowtail butterfly *Protographium marcellus* foraging on flowers. We depict this imagery in honeybee false colors where UV, blue, and green quantum catches are shown as blue, green, and red, respectively. Assessing the conspicuousness of this butterfly against a background of many small inflorescences would be challenging using spectroscopy. Also, note how scarce UV information is in such an average natural scene.
(MP4)

S6 Video A caterpillar’s anti-predator display in *Apis* vision. Conceal and reveal displays can pose a problem for spectroscopy and standard multispectral photography. Here, we show a video of a black swallowtail *Papilio polyxenes* caterpillar displaying its osmeteria. We illustrate this video in honeybee false colors such that UV, blue, and green quantum catches are shown as blue, green, and red, respectively. The (human) yellow osmeteria as well as the yellow spots along the caterpillar’s back both reflect strongly in the UV and appear magenta when the colors are shifted into honeybee false colors (as the strong responses on the honeybee’s UV-sensitive and green-sensitive photoreceptors are depicted as blue and red, respectively). Many predators of caterpillars perceive UV, and accordingly, this coloration might be an effective aposematic signal.
(MP4)

S7 Video A rainbow through the eyes of 4 different animals. Certain scenes would be challenging to measure using traditional methods and would appear entirely distinct to different animal viewers. As an illustration of this point, we show the same rainbow in (A) mouse, (B) honeybee, and (C) avian false colors, alongside a (D) video with standard colors (i.e., human colors). Specifically, for the (A) dichromatic mouse, we illustrate the UV and green-sensitive photoreceptors responses in blue and green, respectively. The red channel is given the average value of the UV and green quantum catches. When depicted in this way the mouse-view video shows the uppermost “green” band as broad and not clearly differentiated from the green background, while the lower, and very broad, UV band (depicted as blue) is visible. We illustrate the (B) honeybee’s UV, blue, and green photoreceptor responses in blue, green, and red, respectively. Using these honeybee false colors, the rainbow seems similar to the typical (D) human perceived rainbow but appears lower on the screen as their 3 photoreceptors are sensitive to lower wavelengths than ours. Finally, for the (C) tetrachromatic bird we illustrate their UV, blue, green, and red photoreceptor responses as magenta, blue, green, and red, respectively. Here, the UV information is overlaid on the human-visible range. The avian-view video is similar to the (D) standard human-view video, except the UV (depicted as magenta) appears below the blue band. Thus, their rainbow has more colors and extends lower on the frame than the (D) human-view rainbow. These “additional” UV color bands are visible in the lower portions of the screen for (A–C) animal-view videos, but not for the video depiction colors as they appear to the (D) typical human viewer.
(MP4)

S8 Video Application of sunscreen in *Apis* vision. Multispectral photography has many potential applications including examining topical effects, for example, tracking UV-absorbing urine tracks or evaluating cosmetics. Here, we show the application of UV-blocking sunscreen in honeybee false colors. As in other depictions, we show the honeybee’s UV, blue, and green photoreceptor responses as blue, green, and red, respectively. Note that the light toned skin (DH) appears similar in honeybee false colors as in human vision, because skin reflectance increases progressively at longer wavelengths. The sunscreen appears white to our eye because it reflects broadly over the human visible range, but it appears yellow in honeybee false colors because it absorbs UV light. Specifically, the honeybee’s UV photoreceptor (shown as blue) receives little light from the areas where the sunscreen has been applied, while their blue and green sensitive photoreceptors (shown as green and red, respectively) continue to capture abundant light. In honeybee false colors, this results in lower blue and higher green and red pixel values, which produces the yellow coloration.
(MP4)

S9 Video Close-up video of a leaf-footed bug egg in *Apis* vision. We illustrate a video depicting 2 eastern leaf-footed bug (*Leptoglossus phyllopus*) eggs. These eggs are approximately 2 mm in diameter and were on the base of a small leaf, which is shown blowing in the wind in honeybee false color (photoreceptors sensitive to UV, blue, and green light are shown as blue, green, and red, respectively). This demonstrates the capability of the system to image small items close up.
(MP4)

S10 Video A jumping spider in *Apis* vision. Here, we merge 2 videos of small (unidentified) jumping spiders in honeybee false colors (displaying the honeybee’s UV, blue, and green photoreceptor responses as blue, green, and red, respectively). These videos illustrate the ability of the camera to focus on small objects; however, the first video highlights the limitations of using manual focus at these close focal distances on fast-moving subjects. In that clip, the spider is only in good focus in some frames and the video was not perfectly aligned. Experimental designs with small, fast-moving organisms like these spiders could mount the camera such that it focuses on a display site. We illustrate that in the second video, which begins at approximately 1 min 11 s.
(MP4)

S11 Video A northern mockingbird (*Mimus polyglottos*) in avian vision. Here, we illustrate 2 northern mockingbirds interacting in a tree, in avian false colors. Specifically, we show blue, green, and red quantum catches as blue, green, and red, respectively, and UV quantum catches are overlaid as magenta. While the 80 mm lens is not designed for imaging distant subjects, the system captures avian-view imagery well and shows the “avian white” (reflective from the UV through the visible portions of the spectrum) patches of their feathers. It also illustrates that the sky as predominantly UV-colored (i.e., appearing magenta), due to shorter wavelengths being subjected to increased Rayleigh scattering. Thus, while the sky may appear blue to our eyes, it would appear UV-blue to many other organisms.
(MP4)

S12 Video Bees foraging and interacting on flowers in *Apis* vision. The camera system is capable to capture naturally occurring behaviors in their original context. This is illustrated with 3 short clips that depict bees foraging (first and second clips) and fighting (third clip) in their natural environment. The videos are shown in honeybee false colors (displaying the honeybee’s UV, blue, and green photoreceptor responses as blue, green, and red, respectively).
(MP4)

S1 Table Error estimations for videos of known standards, taken under full sunlight and normalized to Spectralon standards. Here, we describe how well our estimated animal quantum catches fit to expected animal quantum catches for the honeybee (*Apis* sp.) and the average UVS avian receiver (avian). To more fully assess accuracy, we present mean absolute prediction error (MAPE), root mean squared prediction error (RMSPE) as well as the linear association between camera-predicted and spectrometry-predicted quantum catch (R^2^), and the range of the inner 75% of errors (i.e., excluding the 25% largest absolute errors; 75% error band).
(DOCX)

S2 Table Error estimations for images of known standards, taken under full sunlight and normalized to Spectralon standards. Here, we describe how well our predicted animal quantum catches fit to expected animal quantum catches for the honeybee (*Apis* sp.) and the average UVS avian receiver (avian). To more fully assess accuracy, we present mean absolute prediction error (MAPE), root mean squared prediction error (RMSPE) as well as the linear association between camera-predicted and spectrometry-predicted quantum catch (R^2^), and the range of the inner 75% of errors (i.e., excluding the 25% largest absolute errors; 75% error band).
(DOCX)

S3 Table Error estimations for images of known standards, taken under full sunlight and normalized to ARUCO standards. Here, we describe how well our estimated animal quantum catches fit to expected animal quantum catches for the honeybee (*Apis* sp.) and the average UVS avian receiver (avian). To more fully assess accuracy, we present mean absolute prediction error (MAPE), root mean squared prediction error (RMSPE) as well as the linear association between camera-predicted and spectrometry-predicted quantum catch (R^2^), and the range of the inner 75% of errors (i.e., excluding the 25% largest absolute errors; 75% error band).
(DOCX)

S4 Table Error estimations for images of known standards, taken under lab light and normalized to Spectralon standards. Here, we describe how well our estimated animal quantum catches fit to expected animal quantum catches for the honeybee (*Apis* sp.) and the average UVS avian receiver (avian). To more fully assess accuracy, we present mean absolute prediction error (MAPE), root mean squared prediction error (RMSPE) as well as the linear association between camera-predicted and spectrometry-predicted quantum catch (R^2^), and the range of the inner 75% of errors (i.e., excluding the 25% largest absolute errors; 75% error band).
(DOCX)

S5 Table Error estimations for images of known standards, taken under lab light and normalized to ARUCO standards. Here, we describe how well our estimated animal quantum catches fit to expected animal quantum catches for the honeybee (*Apis* sp.) and the average UVS avian receiver (avian). To more fully assess accuracy, we present mean absolute prediction error (MAPE), root mean squared prediction error (RMSPE) as well as the linear association between camera-predicted and spectrometry-predicted quantum catch (R^2^), and the range of the inner 75% of errors (i.e., excluding the 25% largest absolute errors; 75% error band).
(DOCX)

S6 Table Error estimations for videos of known standards, taken outdoors in shade and normalized to ARUCO standards. Here, we describe how well our estimated animal quantum catches fit to expected animal quantum catches for the honeybee (*Apis* sp.) and the average UVS avian receiver (avian). To more fully assess accuracy, we present mean absolute prediction error (MAPE), root mean squared prediction error (RMSPE) as well as the linear association between camera-predicted and spectrometry-predicted quantum catch (R^2^), and the range of the inner 75% of errors (i.e., excluding the 25% largest absolute errors; 75% error band).
(DOCX)

S7 Table Error estimations for a set of example animals. Here, we describe how well our estimated animal quantum catches fit to expected animal quantum catches for a small set of example animals. The measurements were taken from videos of known standards, recorded in full sunlight and normalized to ARUCO standards. We present the linear association between camera-predicted and spectrometry-predicted quantum catch (R^2^).
(DOCX)

S8 Table Custom color cards. Here, we provide information on the 2 custom color cards that we used in this study. The first was the custom color card with ARUCO markers (S17 Fig), which includes 20 pastels and 8 grayscale patches (see Materials and methods for details). The second was a small color card, visible in a few shots (e.g., S22 Fig). We prefix each color target with a number corresponding with its position on the card (see S17 and S22 Figs) and its position within the reflectance spectra dataset.
(DOCX)

S9 Table The sensor conversion errors for a set of example species. The table contains the R^2^ values of the fit between the photoreceptor quantum catches calculated directly from reflectances vs. estimated from camera catches with the transformation matrix. The fit was evaluated on a reserved testing library of 250 spectra from FReD [59]. The coefficient of determination exceeds 0.958 on all bands, for all animals.
(DOCX)

S10 Table The sensor conversion errors for the *Apis mellifera* receptors. The table contains the R^2^ values of the fit between the photoreceptor quantum catches calculated directly from reflectances vs. estimated from camera catches with the transformation matrix. The fit was evaluated on a reserved testing library of 250 spectra from FReD [59], for the illuminations shown on S16 Fig. When making predictions for ideal illumination, the coefficient of determination of the *Apis* photoreceptors exceeds 0.958 on all bands. The performance is similar for various target illuminations.
(DOCX)

S11 Table The sensor conversion errors for the avian sp. **receptors.** The table contains the R^2^ values of the fit between the photoreceptor quantum catches calculated directly from reflectances vs. estimated from camera catches with the transformation matrix. The fit was evaluated on a reserved testing library of 250 spectra from FReD [59], for the illuminations shown on S16 Fig. When making predictions for ideal illumination, the coefficient of determination of the avian photoreceptors exceeds 0.972 on all bands. The performance is worse for the UV receptor when using nonideal illumination, with the lab illumination having the highest error.
(DOCX)

S12 Table The sensor conversion errors for the *Apis mellifera* receptors when evaluated on the USGS library. The table contains the R^2^ values of the fit between the photoreceptor quantum catches calculated directly from reflectances vs. estimated from camera catches with the transformation matrix. The fit was evaluated on reflectances downloaded from the USGS Spectral Library [61], for the illuminations shown on S16 Fig. When testing using the USGS database, the coefficient of determination of the *Apis* photoreceptors exceeds 0.99 on all bands, irrespective of the target illumination.
(DOCX)

S13 Table The sensor conversion errors for the avian receptors when evaluated on the USGS library. The table contains the R^2^ values of the fit between the photoreceptor quantum catches calculated directly from reflectances vs. estimated from camera catches with the transformation matrix. The fit was evaluated on reflectances downloaded from the USGS Spectral Library [61], for the illuminations shown on S16 Fig. When testing using the USGS database, the coefficient of determination of the avian photoreceptors exceeds 0.997 on all bands, irrespective of the target illumination.
(DOCX)

S14 Table Natural objects tested. Here, we provide information on the natural objects we used in this study, indicating the region and how that region was coded on spectral reflectance measurements.
(DOCX)

S15 Table Error estimations for prediction for images of natural objects, taken under full sunlight and normalized to ARUCO standards. Here, we describe how well our estimated animal quantum catches fit to spectrometry-predicted animal quantum catches for the honeybee (*Apis* sp.) and the average UVS avian receiver (avian). To more fully assess accuracy, we present mean absolute prediction error (MAPE), root mean squared prediction error (RMSPE) as well as the linear association between camera-predicted and spectrometer-predicted quantum catch (R^2^), and the range of the inner 75% of errors (i.e., excluding the 25% largest absolute errors; 75% error band).
(DOCX)

S16 Table RAW to JPG conversion error. The mean absolute error associated with the reconstruction of the RAW values using Sony’s formula (JPG Conversion MAE) and the mean absolute error associated with the losses due to quantization and the lossy compression of the JPG algorithm.
(DOCX)

S1 Fig Evaluating the fit for videos of known standards. In this case, the videos were taken under full sunlight and normalized to a set of Spectralon standards. The plots show the animal quantum catch predicted from reflectance (Spectrometer-predicted AC) against our camera-predicted animal quantum catch (Camera-predicted AC). We plot the fit for known color standards (our custom ARUCO standard, another set of pastels and a DKK Color Calibration Chart), and for both the honeybee (*Apis* sp., top) and the average ultraviolet sensitive avian receiver (Avian sp., bottom), for each of their 3 and 4 photoreceptors, respectively. The marker colors indicate the human-perceived color of the sample. For data on fit, please see S1 Table. The data underlying this figure can be found in S1 Data.
(TIF)

S2 Fig Evaluating the fit for images of known standards. In this case, the images were taken under full sunlight and normalized to a set of Spectralon standards. The plots show the animal quantum catch predicted from reflectance (Spectrometer-predicted AC) against our camera-predicted animal quantum catch (Camera-predicted AC). We plot the fit for known color standards (our custom ARUCO standard, another set of pastels and a DKK Color Calibration Chart), and for both the honeybee (*Apis* sp., top) and the average ultraviolet sensitive avian receiver (Avian sp., bottom), for each of their 3 and 4 photoreceptors, respectively. The marker colors indicate the human-perceived color of the sample. For data on fit, please see S2 Table. The data underlying this figure can be found in S1 Data.
(TIF)

S3 Fig Evaluating the fit for images of known standards when normalizing to the custom grayscale. In this case, the images were taken under full sunlight and normalized to a set of ARUCO standards. The plots show the animal quantum catch predicted from reflectance (Spectrometer-predicted AC) against our camera-predicted animal quantum catch (Camera-predicted AC). We plot the fit for known color standards (our custom ARUCO standard, another set of pastels and a DKK Color Calibration Chart), and for both the honeybee (*Apis* sp., top) and the average ultraviolet sensitive avian receiver (Avian sp., bottom), for each of their 3 and 4 photoreceptors, respectively. The marker colors indicate the human-perceived color of the sample. For data on fit, please see S3 Table. The data underlying this figure can be found in S1 Data.
(TIF)

S4 Fig Evaluating the fit for images of known standards under lab light. In this case, the images were taken under lab light and normalized to a set of Spectralon standards. The plots show the animal quantum catch predicted from reflectance (Spectrometer-predicted AC) against our camera-predicted animal quantum catch (Camera-predicted AC). We plot the fit for known color standards (our custom ARUCO standard, another set of pastels and a DKK Color Calibration Chart), and for both the honeybee (*Apis* sp., top) and the average ultraviolet sensitive avian receiver (Avian sp., bottom), for each of their 3 and 4 photoreceptors, respectively. The marker colors indicate the human-perceived color of the sample. For data on fit, please see S4 Table. The data underlying this figure can be found in S1 Data.
(TIF)

S5 Fig Evaluating the fit for images of known standards under lab light when normalizing to a custom grayscale. In this case, the images were taken under lab light and normalized to a set of ARUCO standards. The plots show the animal quantum catch predicted from reflectance (Spectrometer-predicted AC) against our camera-predicted animal quantum catch (Camera-predicted AC). We plot the fit for known color standards (our custom ARUCO standard, another set of pastels and a DKK Color Calibration Chart), and for both the honeybee (*Apis* sp., top) and the average ultraviolet sensitive avian receiver (Avian sp., bottom), for each of their 3 and 4 photoreceptors, respectively. The marker colors indicate the human-perceived color of the sample. For data on fit, please see S5 Table. The data underlying this figure can be found in S1 Data.
(TIF)

S6 Fig Evaluating the fit for videos of known standards, recorded in shade. In this case, the videos were taken in shade outdoors and normalized to a set of ARUCO standards. The plots show the animal quantum catch predicted from reflectance (Spectrometer-predicted AC) against our camera-predicted animal quantum catch (Camera-predicted AC). We plot the fit for known color standards (our custom ARUCO standard, another set of pastels and a DKK Color Calibration Chart), and for both the honeybee (*Apis* sp., top) and the average ultraviolet sensitive avian receiver (Avian sp., bottom), for each of their 3 and 4 photoreceptors, respectively. The marker colors indicate the human-perceived color of the sample. For data on fit, please see S6 Table. The data underlying this figure can be found in S1 Data.
(TIF)

S7 Fig Example false color image. A black-eyed Susan (*Rudbeckia hirta*) depicted as a honeybee (*Apis mellifera*) false color image and the same flower in a human-vision. In honeybee false color images, the blue, green, and red channels represent quantum catches of their UV-, blue-, and green-sensitive photoreceptors, respectively. We provide a visual key (bottom left corner) illustrating each of the bee’s 3 photoreceptors (vertices) and the colors used to represent the variable stimulation of these 3 photoreceptors (interior colors). This flower has a nectar guide that aids recruitment [43]. To our eye, the black-eyed Susan appears entirely yellow because it reflects primarily long-wavelength light in the human-visible range. Whereas in the bee false color image, the distal petals appear magenta because they reflect UV in addition to long-wavelength light, stimulating both the photoreceptors sensitive to UV (depicted as blue) and those sensitive to green light (depicted as red). By contrast, the central portion of the petals does not reflect UV and therefore appears red. We applied a gamma correction to the honeybee false color and linear (human) image for display purposes (AC^0.3^ and CC^0.5^). These are the same images as shown in Fig 1.
(TIF)

S8 Fig Modular 3D printed housing. (A) Schematic showing the assembled cage, with mounting points for the (i) visible light Sony camera, the (ii) full-spectrum modified camera. The complete set of parts are as follows: (B) Side 2, (C) Side 1, (D) Base, (E) Bellows Cube Face, (F) Rear Camera Face, (G) Top Camera Face, (H) Mirror Latch, (I) Mirror Mount, (J) Internal Bracket, (K) Internal Bracket (mirrored), (L) UV Bandpass Filter Holder, (M) Internal Shroud, (N) Camera Base Connector, (O) UV Bandpass Cone, (P) Visible Cone Baffle, and 3 options for Bellows Lens Mounts, for mounting (Q) 80 mm f5.6, (R) 135mm f5.6, or (S) 210 mm f5.6 Nikon EL-Nikkor Enlarging lenses. We printed the parts using a Prusa i3 MKS+ 3D printer, with and without the MMU2s+, from black PLA and PETG filament (Hatchbox) with 0.15 mm QUALITY presets in PrusaSlicer. Higher quality prints are recommended for regular field use. The STL files of all the parts required to print a system, along with a complete bill of materials and detailed instructions for printing and assembly, are freely available from the Gitlab Repository https://gitlab.com/multispectrum-beamsplitter. We have also included a set of 3MF files that can be opened with the free PrusaSlicer software. These files illustrate the recommended print orientation of each file and highlight the few parts that require supports. Future updates will be provided via the repository as the design continues to evolve and improve.
(TIF)

S9 Fig Modular 3D printed bellows. We recommend using commercially available bellows such as the Novoflex BALLPRO due to their robustness, but provide plans for a DIY option as an alternative. (A) Schematic showing the assembled DIY sliding bellow system, attached to the housing, with a (i) lens and bag bellows attachment plate, (ii) rail to allow for focusing, and a (iii) spacer. The complete sets of parts are as follows: (B) Base Extension, (C) Rail, (D) Rail End Stop, (E) Cube Face, (F) Bag Bellows Cube Mount, (G) Bag Bellows Lens Mount, (H) Sliding Plate. In addition, the DIY bellows require semi stiff fabric that is light tight (e.g., faux black leather). The fabric was cut using a laser and then assembled with contact cement. We printed the parts using a Prusa i3 MKS+ 3D printer, with and without the MMU2s+, from black PLA (Hatchbox) with 0.15 mm QUALITY presets in PrusaSlicer. The files and information to print the system is available from the Gitlab Repository https://gitlab.com/multispectrum-beamsplitter.
(TIF)

S10 Fig Capturing polarized light. Fluctuations in the pixel values from the UV (A) and the visible camera (B) as a function of the polarization plane of the illuminating light. Photos were taken of the exit port of an integrating sphere (StellarNet IC2), illuminated with broadband light (full-spectrum xenon bulb, Thorlabs, SLS 202 through 1,000 μm fiber optics, Edmund Optics, 58–458), through a polarization filter positioned at varying rotational angles. Example photos are shown in the insets. UV light with a polarization plane of 90°, i.e., horizontally polarized, is less likely to be reflected by the beam splitter than vertically polarized UV light, and up to 0.6% of horizontally polarized light may be blocked in the UV channel. On the other hand, visible light with a polarization plane of 0°, i.e., vertically polarized light, is less likely to be transmitted by the beam splitter than horizontally polarized visible light, and up to 2.4, 0.8, and 1.0% of the polarized light may be blocked in the blue, green, and red channels. The effect is too small (approximately 1 to 2 pixel value) to interfere with the intended use of the camera; however, care must be taken if the system is adapted for recording polarized light. The data underlying this figure can be found in S1 Data.
(TIF)

S11 Fig Illumination used for estimating sensor sensitivities. We used a xenon light source (SLS 205, Thorlabs) connected to a monochromator (Optimetrics, DMC1-03) to deliver narrow bands of light (colored bands, mean FWHM ± s.e. = 7.3 ± 0.29 nm) from 280 nm to 800 nm. The total power used for estimation (solid black line) was the sum photon flux (μmol s^−1^ m^−2^). To depict summed data (solid black line) and individual spectra (colorful) plots on the same display, individual spectra were multiplied by 6.5. Second order scatter is visible from approximately 300 nm to 350 nm as smaller orange to red peaks when illuminated with light from 600 nm to 700 nm. These were removed (see Materials and methods) and we replicated these camera spectral sensitivity measurements using another approach that omitted second order scatter using longpass filters (for more details, see Method A in S1 Text). The data underlying this figure can be found in S1 Data.
(TIF)

S12 Fig Comparison of camera sensor sensitivity measurements using 2 methods over 2 ranges. Relative camera sensor sensitivity for the ultraviolet (purple lines), blue (blue lines), green (green lines), and red (red lines) sensors, measured from 300 nm to 1,100 nm (solid) or 300 nm to 700 nm (dashed). Using longpass filters, we excluded second order scatter over the extended range (for details, see Method A in S1 Text) and confirmed these effects did not impact our readings over the reduced range (see Materials and methods). To ensure the data were comparable, we relativized both curves over the 300 nm to 700 nm range. For the extended range (solid) sensitivities above 700 nm were negligible for the ultraviolet, blue, green, and red sensors. The data underlying this figure can be found in S1 Data.
(TIF)

S13 Fig The infrared sensitivity of the camera. We photographed a set of 8 Spectralon standards under direct sunlight with abundant IR light (the total irradiance measured in μWatt/cm^2^ in the infrared, 700 nm to 1,100 nm, spectrum was 5 to 6 times higher than in the ultraviolet, 300 nm to 400 nm, spectrum). We set both cameras at the same shutter speeds (1/4,000) and sequentially increased this a stop for each camera up to shutter speeds of 30 s. Photos were taken under 3 conditions: as normal (“open” condition), with the camera cap on to quantify dark noise (“capped” condition), while in the third, “filter” condition we placed a set of 2 longpass filters directly in front of the lens (MidOpt LP715-25 and a Semrock BLP01-532R-25) that collectively blocked all light other than near infrared. (A) Representative photos at 4 exposure speeds show that both the UV and the visible camera are capable of registering infrared light. (B) The pixel values of the white Spectralon standard (99% reflective up to 2,300 nm) show that the intended UV and VIS image saturate before the IR signal becomes detectable. At the shutter speeds required for UV photography (indicated by arrows) the infrared contamination is statistically indistinguishable from dark noise (comparison of pixel values from the “capped” condition vs. from the “filter” condition by a Z-test: UV: *p* = 0.743; blue: *p* = 0.960; green: *p* = 0.841; red: *p* = 0.985). The data underlying this figure can be found in S1 Data.
(TIF)

S14 Fig Custom color standard. We used a custom color card (A) that featured 4 ARUCO fiducial markers and 28 distinct colors. The color patches were made from pastels (positions 0–5 and 14–27) or a mixture of barium sulfate (white) and flat black (Black 3.0) paints (positions 6–13) that are isoluminant across the 300 nm to 700 nm wavelength range (B). The use of the custom color standard enables fast, highly accurate, automated methods for calibration, normalization, and transformation. For details on how to construct these cards, see S8 Table. The data underlying this figure can be found in S1 Data.
(TIF)

S15 Fig The accuracy of the linearization and normalization steps. The linearization is highly accurate, regardless of whether the grayscale standards are Spectralon standards or the Barium-sulfate mixes on our ARUCO custom color card, and whether the exact Sony formula or an approximate power transform is used. The data underlying this figure can be found in S1 Data.
(TIF)

S16 Fig The example illumination functions used in the study. “Ideal” refers to the isoluminant illumination, “sunlight” to the standard d65 illumination, “forest” was taken from the PAVO package, and “lab” is the metal halide lamp in our lab. The data underlying this figure can be found S1 Data.
(TIF)

S17 Fig The photoreceptor sensitivities of the 2 example animals, the Western honeybee *Apis mellifera* [57,58] and the average UV-sensitive bird [45,58]. The photoreceptors have peak sensitivities in the ultraviolet (dotted black line), blue (dashed blue line), green (green line), and red (dot-dashed red line) parts of the spectrum. The data underlying this figure can be found in S1 Data.
(TIF)

S18 Fig The accuracy of the transformation step in the training library. The figure shows the relationship between the photoreceptor quantum catches calculated directly from reflectances (AC from reflectance) vs. estimated from camera catches with the transformation matrix (AC from camera catch). In this paper, we used 2,494 spectra from the FReD database [59] to derive a transformation matrix, *T* that converts linear camera catches to animal catches. Here, we illustrate the fit for those relationships. R^2^ values for the full library are shown (rather than the testing library used to test accuracy). The data underlying this figure can be found in S1 Data.
(TIF)

S19 Fig The sensor conversion error associated with the transformation matrix. The figure shows the R^2^ values of the fit between the photoreceptor quantum catches calculated directly from reflectances vs. estimated from camera catches with the transformation matrix. The fit was evaluated using a set of reflectances reserved for testing from the FReD (solid lines) and on the USGS Spectral Library (dashed lines), for a synthetically generated library of photoreceptors with peak sensitivities between 300 nm and 700 nm, based on the A1 (A) and A2 templates (B) from [60]. The performance is reliably R^2^ > 0.90 (blue dotted line), in most cases exceeding 0.99, for all wavelengths between 343 nm and 700 nm. The system only becomes inaccurate at the extreme end of the ultraviolet, past 340 nm, for which the ultraviolet camera has little sensitivity. This suggests that any organism with peak photoreceptor sensitivities falling in this range (the “effective range” of the camera) should produce accurate results. Note however that the A1 and A2 templates used above are approximations, and the precise fit for a particular organism will depend on the exact shape of its photoreceptors’ sensitivities. For example, the sensitivity of the honeybee’s UV receptor is narrower and less sensitive to far-UV than the template, and so its fit is better than predicted here (R^2^ = 0.958 when testing on FReD, R^2^ = 0.991when testing on USGS, see Tables S10 and S12). The data underlying this figure can be found in S1 Data.
(TIF)

S20 Fig The sensor conversion error associated with the transformation matrix, under various viewing illuminations. The figure shows the R^2^ values of the fit between the photoreceptor quantum catches calculated directly from reflectances vs. estimated from camera catches with the transformation matrix. The fit was evaluated using a set of reflectances reserved for testing from the FReD, for a synthetically generated library of photoreceptors with peak sensitivities between 300 nm and 700 nm, based on the A1 (A) and A2 templates (B) from [60], for 4 viewing illuminations: ideal (black solid line), direct sunlight (yellowish green dashed line), forest shade (green dotted line), and the metal halide lamp in the lab (blue dot-dashed line). The performance between 343 nm to 700 nm is reliably R^2^ > 0.90 (blue dotted line), in most cases exceeding 0.99, for all cases tested, indicating that the method is not sensitive to the viewing illumination. The data underlying this figure can be found in S1 Data.
(TIF)

S21 Fig The sensor conversion error for narrowband spectra. The absolute error of the transformation is shown as function of the peak reflectance (x axis) and the receptor’s peak sensitivity (y axis), for reflectance spectra with 3 different values of full-width-at-half-maximum (FWHM). In this analysis, we used simulated reflectance spectra, assumed to follow a Gaussian distribution, and the standard photoreceptor template from [60]. The performance remains reasonable (<0.017 absolute error) even for hypothetical materials with reflectance spectra that have a full-width-at-half-maximum of 10 nm. Minor errors appear when estimating the response of a short wavelength sensitive receptor (peak sensitivity <400 nm) to very narrow emissions in the far-UV (<340 nm) and around approximately 390 nm where the camera has a sensitivity gap. The data underlying this figure can be found in S1 Data.
(TIF)

S22 Fig Examples of honeybee false color images. Here, we illustrate a (A, B) summer snowflake *Leucojum aestivum*, (C, D) blue phlox *Phlox divaricata*, and a (D, E) blue violet *Viola sororia* in honeybee false color (left) and human-visible colors (right). We also show a simple, cheap, pastel-based color standard that we used to validate animal-perceived quantum catches (A, B). We applied a gamma correction to the images (AC_i_^0.3^ and CC_i_^0.5^, respectively).
(TIF)

S23 Fig Evaluating the fit for images of natural objects. In this case, the images were taken under full sunlight and normalized to a set of ARUCO standards. The plots show the animal quantum catch predicted from reflectance (Spectrometer-predicted AC) against our camera-predicted animal quantum catch (Camera-predicted AC). We plot the fit for a collection of flowers (diamonds), leaves (triangles), birds’ eggs (squares), and birds’ feathers (circles); see S14 Table for sample details. The fit is shown for both the honeybee (*Apis* sp., top) and the average ultraviolet-sensitive avian receiver (Avian sp., bottom), for each of their 3 and 4 photoreceptors, respectively. The linear relationship between the 2 estimates was weaker for natural objects than for color standards. The mismatch represents a meaningful variation: the perceived color of natural objects is altered by their shape, fine patterning, texture, and physical color. The marker colors indicate the human-perceived color of the sample. For data on fit, please see S15 Table. The data underlying this figure can be found in S1 Data.
(TIF)

S24 Fig Example images of natural objects. A display illustrating a subset of natural objects we used for assessing the accuracy of our system (S23 Fig and S14 and S15 Tables). We show (A) a white oak *Quercus alba* leaf, (B) a European starling *Sturnus vulgaris* specimen, (C) gray catbird *Dumetella carolinensis* egg, and (D) a bouquet of flowers. The correlation between spectrometry-predicted and camera-predicted animal catches is weaker for natural objects than for pastels (S23 Fig and S15 Table). The samples above illustrate the fact that the shape of stimuli, such as (A) leaves, (C) eggs, interacts with the direction of illuminating light and therefore impacts the appearance of surface colors. In some cases, such as (B) feather iridescence certain colors may (correctly) appear on an image but not on diffuse reflectance measurements. Finally, many objects such as flowers on (D) occlude and shade themselves and other plants. In all of these situations multispectral photography most likely provides a closer approximation to the quantum catches of free-living organisms than spectroscopy.
(TIF)

S25 Fig Example animal-view videos capturing colors otherwise hard to measure. Our camera system provides a method that will allow researchers to accurately capture scenes in relative quantum catches units, in scenarios that would be challenging for other methods. For example, we illustrate a (A) zebra swallowtail butterfly *Protographium marcellus* moving between flowers, where assessing the color contrast between the butterfly and the background of flowers and leaves would be challenging to measure using spectroscopy. We show a (B) black swallowtail *Papilio polyxenes* caterpillar revealing its otherwise concealed osmeteria. This brief display would be impossible to measure using spectroscopy or traditional multispectral photography. We also illustrate a (C) rainbow, as an example of an optical effect that would be hard to accurately capture with any other method. Finally, we illustrate the (D) application of UV-blocking sunscreen, which would be measurable using other methods but not as a continuous movement. All frames are plotted in honeybee false color, where bee perceived colors are shifted into the human-visible space (i.e., UV, blue, and green quantum catch images are depicted as blue, green, and red in the false color image). The videos are available as S5–S8 Videos.
(TIF)

S26 Fig The correspondence between human (top row) and RNL false colors (bottom) for a set of example colors. Left to right: ultraviolet, ultraviolet-blue, blue, blue-green, green, dark yellow, red, and gray.
(TIF)

S1 Text Supplementary methods. Extended information regarding the RNL color space (Method A), Spectroscopy (Method B), our approaches for image and video alignment (Method C), detailed information about SONY’s S-Log3 format (Method D), and tests of our system’s performance on narrowband reflectance (Method E).
(DOCX)

S1 Data Supporting Data. Numerical data underlying the plots in Figs 3, 5, 6, S1–S6, S10–S21, and S23.
(ZIP)

## Abbreviations

ARUCO: Augmented Reality University of Cordoba
ECC: enhanced correlation coefficient
FReD: Floral Reflectance Database
MAPE: mean absolute prediction error
RMSPE: root mean squared prediction error
