# Supplementary material for: Recording animal-view videos of the natural world using a novel camera system and software package
Source: PLoS Biol. 2024 Jan 23;22(1):e3002444. doi: 10.1371/journal.pbio.3002444 (PMC10805291; doi:10.1371/journal.pbio.3002444)
Supplement: S3 Table — Here, we describe how well our estimated animal quantum catches fit to expected animal quantum catches for the honeybee (Apis sp.) and the average UVS avian receiver (avian). To more fully assess accuracy, we present mean absolute prediction error (MAPE), root mean squared prediction error (RMSPE) as well as the linear association between camera-predicted and spectrometry-predicted quantum catch (R2), and the range of the inner 75% of errors (i.e., excluding the 25% largest absolute errors; 75% error band). (DOCX) [file pbio.3002444.s015.docx]

| **Visual system** | **Channel** | **RMSPE** | **MAPE** | **R^2^** | **75% error band** |
| --- | --- | --- | --- | --- | --- |
| *Apis* | UV | 0.036 | 0.031 | 0.976 | [-0.039; 0.041] |
|  | blue | 0.049 | 0.039 | 0.962 | [-0.055; 0.048] |
|  | green | 0.037 | 0.030 | 0.983 | [-0.039; 0.042] |
| Avian | UV | 0.035 | 0.029 | 0.979 | [-0.039; 0.039] |
|  | blue | 0.050 | 0.041 | 0.969 | [-0.058; 0.059] |
|  | green | 0.070 | 0.050 | 0.967 | [-0.065; 0.070] |
|  | red | 0.066 | 0.046 | 0.974 | [-0.054; 0.043] |
