# Supplementary material for: Recording animal-view videos of the natural world using a novel camera system and software package
Source: PLoS Biol. 2024 Jan 23;22(1):e3002444. doi: 10.1371/journal.pbio.3002444 (PMC10805291; doi:10.1371/journal.pbio.3002444)
Supplement: S7 Table — Here, we describe how well our estimated animal quantum catches fit to expected animal quantum catches for a small set of example animals. The measurements were taken from videos of known standards, recorded in full sunlight and normalized to ARUCO standards. We present the linear association between camera-predicted and spectrometry-predicted quantum catch (R2). (DOCX) [file pbio.3002444.s019.docx]

| **Animal** | **UV** | **Blue** | **Green** | **Red** |
| --- | --- | --- | --- | --- |
| Honeybee  (*Apis mellifera*)^1^ | 0.942 | 0.970 | 0.977 | - |
| Ultraviolet-sensitive bird (avian sp.)^2^ | 0.971 | 0.970 | 0.963 | 0.978 |
| Buff-tailed bumblebee (*Bombus terrestris dalmaticus*)^3^ | 0.946 | 0.964 | 0.977 | - |
| Jumping spider  (unspecified)^4^ | 0.971 | - | 0.976 | 0.963 |
| Domestic chick (*Gallus gallus*)^5^ | 0.969 | 0.965 | 0.966 | 0.980 |
| Bluetit  (*Cyanistes caeruleus*)^4^ | 0.974 | 0.970 | 0.964 | 0.980 |
| Peafowl  (*Pavo cristatus*)^4^ | 0.967 | 0.941 | 0.962 | 0.978 |
| Dog  (*Canis lupus familiaris*)^4^ | - | 0.968 | 0.979 | - |
| Mouse (*Mus musculus*)^6^ | 0.964 | - | 0.968 | - |

^1^ Peitsch et al. 1992, Maia et al. 2013

^2^ Ender and Mielke 2005, Maia et al. 2013

^3^ Skorupski et al. 2007

^4^ micaToolbox, Troscianko and Stevens 2015

^5^ Osorio et al. 1999

^6^ de Farias Rocha et al. 2016
