# Supplementary material for: Recording animal-view videos of the natural world using a novel camera system and software package
Source: PLoS Biol. 2024 Jan 23;22(1):e3002444. doi: 10.1371/journal.pbio.3002444 (PMC10805291; doi:10.1371/journal.pbio.3002444)
Supplement: S8 Table — Here, we provide information on the 2 custom color cards that we used in this study. The first was the custom color card with ARUCO markers (S17 Fig), which includes 20 pastels and 8 grayscale patches (see Materials and methods for details). The second was a small color card, visible in a few shots (e.g., S22 Fig). We prefix each color target with a number corresponding with its position on the card (see S17 and S22 Figs) and its position within the reflectance spectra dataset. (DOCX) [file pbio.3002444.s020.docx]

| **Standard** | **Material** | **Manufacturer descriptions** | **Target description** |
| --- | --- | --- | --- |
| ARUCO standard | Blick Artists' Soft Pastel | 20 Artists' Pastels Half Sticks. 21948-1209 | (1) Black^1^, (2) White^1^, (3) Cool Grey 3^1^, (4) Burnt Umber 4, (5) Burnt Sienna 4, (6) Raw Sienna 2 |
|  | Barium sulfate paint and black paint | Labsphere 6080, and Culture Hustle, Black 3.0 | (7) ~99%, (8) ~70%, (9) ~50%, (10) ~43%, (11) ~16%, (12) ~11%, (13) ~5%, (14) ~3% |
|  | Blick Artists' Soft Pastel | 20 Artists' Pastels Half Sticks. 21948-1209 | (15) Yellow Ochre 4, (16) Olive Green 4^2^, (17) Sap Green 2, (18) Viridian Hue 4^2^, (19) Phthalo Blue 3 (Green Shade), (20) Prussian Blue 1, (21) Ultramarine 4, (22) Purple 3, (23) Crimson Lake 1, (24) Cadmium Red Hue 4, (25) Cadmium Red Orange Hue 4, (26) Cadmium Orange Hue 2, (27) Yellow 3, (28) Lemon Yellow 2 |
| Pastel card | Blick Artists' Soft Pastel | 20079-XXXX^3^ | (1) White^1^, (2) Cool gray 1, (3) Sepia 1, (4) Cool gray 2, (5) Cool gray 3^1^, (6) Cool gray 4, (7) Blue gray 4, (8) Black^1^, (9) Phthalo blue (red shade) 4, (10) Lemon yellow 4, (11) Bright green 4, (12) Crimson lake 4, (13) Purple brown 1 |
| \| ^1^ This color was present in both color cards, though validation tests were conducted using reflectance spectra from each sample. ^2^ This color did not properly adhere to the surface and was excluded from tests. ^3^ These colors were purchased individually, and each would have a different serial number in the 20079 series. \| \| --- \| | | | |
