# Supplementary material for: Recording animal-view videos of the natural world using a novel camera system and software package
Source: PLoS Biol. 2024 Jan 23;22(1):e3002444. doi: 10.1371/journal.pbio.3002444 (PMC10805291; doi:10.1371/journal.pbio.3002444)
Supplement: S9 Table — The table contains the R2 values of the fit between the photoreceptor quantum catches calculated directly from reflectances vs. estimated from camera catches with the transformation matrix. The fit was evaluated on a reserved testing library of 250 spectra from FReD [59]. The coefficient of determination exceeds 0.958 on all bands, for all animals. (DOCX) [file pbio.3002444.s021.docx]

| **Animal** | **UV** | **Blue** | **Green** | **Red** |
| --- | --- | --- | --- | --- |
| Honeybee  (*Apis mellifera*)^1^ | 0.958 | 0.978 | 1.000 | - |
| Ultraviolet-sensitive bird (avian sp.)^2^ | 0.972 | 1.000 | 0.998 | 1.000 |
| Buff-tailed bumblebee (*Bombus terrestris dalmaticus*)^3^ | 0.958 | 0.983 | 1.000 | - |
| Jumping spider  (unspecified)^4^ | 0.963 | - | 1.000 | 0.984 |
| Domestic chick (*Gallus gallus*)^5^ | 0.972 | 0.999 | 0.999 | 1.000 |
| Bluetit  (*Cyanistes caeruleus*)^4^ | 0.982 | 0.998 | 0.999 | 1.000 |
| Peafowl  (*Pavo cristatus*)^4^ | 0.974 | 0.995 | 0.997 | 0.999 |
| Dog  (*Canis lupus familiaris*)^4^ | - | 0.982 | 0.999 | - |
| Mouse (*Mus musculus*)^6^ | 0.983 | - | 0.999 | - |

^1^ Peitsch et al. 1992, Maia et al. 2013

^2^ Ender and Mielke 2005, Maia et al. 2013

^3^ Skorupski et al. 2007

^4^ micaToolbox, Troscianko and Stevens 2015

^5^ Osorio et al. 1999

^6^ de Farias Rocha et al. 2016
