# Supplementary material for: Recording animal-view videos of the natural world using a novel camera system and software package
Source: PLoS Biol. 2024 Jan 23;22(1):e3002444. doi: 10.1371/journal.pbio.3002444 (PMC10805291; doi:10.1371/journal.pbio.3002444)
Supplement: S10 Table — The table contains the R2 values of the fit between the photoreceptor quantum catches calculated directly from reflectances vs. estimated from camera catches with the transformation matrix. The fit was evaluated on a reserved testing library of 250 spectra from FReD [59], for the illuminations shown on S16 Fig. When making predictions for ideal illumination, the coefficient of determination of the Apis photoreceptors exceeds 0.958 on all bands. The performance is similar for various target illuminations. (DOCX) [file pbio.3002444.s022.docx]

| **Illumination** | **Apis - UV** | | **Apis - Blue** | | **Apis - Green** | |
| --- | --- | --- | --- | --- | --- | --- |
| ideal | | 0.958 | | 0.978 | | 1.000 |
| sunlight | | 0.979 | | 0.984 | | 1.000 |
| forest | | 0.972 | | 0.984 | | 1.000 |
| lab | | 0.955 | | 0.984 | | 1.000 |
