# Supplementary material for: Recording animal-view videos of the natural world using a novel camera system and software package
Source: PLoS Biol. 2024 Jan 23;22(1):e3002444. doi: 10.1371/journal.pbio.3002444 (PMC10805291; doi:10.1371/journal.pbio.3002444)
Supplement: S12 Table — The table contains the R2 values of the fit between the photoreceptor quantum catches calculated directly from reflectances vs. estimated from camera catches with the transformation matrix. The fit was evaluated on reflectances downloaded from the USGS Spectral Library [61], for the illuminations shown on S16 Fig. When testing using the USGS database, the coefficient of determination of the Apis photoreceptors exceeds 0.99 on all bands, irrespective of the target illumination. (DOCX) [file pbio.3002444.s024.docx]

| **Illumination** | **Apis - UV** | **Apis - Blue** | **Apis - Green** |
| --- | --- | --- | --- |
| ideal | 0.991 | 0.999 | 1.000 |
| sunlight | 0.999 | 0.999 | 1.000 |
| forest | 0.997 | 0.999 | 1.000 |
| lab | 0.999 | 0.999 | 1.000 |
