# Supplementary material for: Recording animal-view videos of the natural world using a novel camera system and software package
Source: PLoS Biol. 2024 Jan 23;22(1):e3002444. doi: 10.1371/journal.pbio.3002444 (PMC10805291; doi:10.1371/journal.pbio.3002444)
Supplement: S14 Table — Here, we provide information on the natural objects we used in this study, indicating the region and how that region was coded on spectral reflectance measurements. (DOCX) [file pbio.3002444.s026.docx]

| **Common name** | **Species** | **Specimen type** | **Regions/ Measurement IDs** | |  |
| --- | --- | --- | --- | --- | --- |
| Showy goldeneye | *Heliomeris multiflora* | flower | | tip, base | |
| Marsh mallow | *Althaea officinalis* | flower | | tip, base | |
| Black-eyed Susan | *Rudbeckia hirta* | flower | | tip base | |
| Maximilian sunflower | *Helianthus maximiliani* | flower | | tip, base | |
| Fameflower | *Talinum paniculatum* | flower | | tip, base | |
| Everlasting pea | *Lathyrus latifoliés* | flower | | tip | |
| Burning bush | *Euonymus alatus* | leaf | | leaf 1, leaf 4, leaf 8, leaf 14, leaf 19, leaf 23, leaf 25, leaf 29, leaf 32, leaf 36 | |
| Shadblow serviceberry | *Amelanchier canadensis* | leaf | | leaf 2, leaf 10, leaf 13, leaf 31, leaf 34 | |
| Eastern black walnut | *Juglans nigra* | leaf | | leaf 3, leaf 12 | |
| American bittersweet | *Celastrus scandens* | leaf | | leaf 5 | |
| Golden raintree | *Koelreuteria paniculata* | leaf | | leaf 6, leaf 7, leaf 18, leaf 26, leaf 37 | |
| Pignut Hickory | *Carya glabra* | leaf | | leaf 9, leaf 16, leaf 21, leaf 22, leaf 33, leaf 35 | |
| White oak | *Quercus alba* | leaf | | leaf 11 (back) | |
| Northern red oak | *Quercus rubra* | leaf | | leaf 17, leaf 20 | |
| Victoria creeper | *Parthenocissus quinquefolia* | leaf | | leaf 27 | |
| Fox grape | *Vitis vulpina* | leaf | | leaf 28, leaf 39 | |
| Hackberry | *Prunus padus* | leaf | | leaf 30 | |
| Bur oak | *Quercus macrocarpa* | leaf | | leaf 38 | |
| Black locust | *Robinia pseudoacacia* | leaf | | leaf 40 | |
| Japanese quail | *Coturnix coturnix* | egg^1^ | | egg A ground, egg A spot, egg A spot, egg B ground | |
| American robin | *Turdus migratorius* | egg^2^ | | egg A ground, egg B ground | |
| Gray catbird | *Dumetella carolinensis* | egg^2^ | | egg A ground, egg B ground | |
| Brown thrasher | *Toxostoma rufum* | egg^2^ | | egg A ground, egg B ground, egg C ground | |
| Northern mockingbird | *Mimus polyglottos* | egg^2^ | | egg A ground with spots mixed, egg A blue ground, egg A spot, egg B ground, egg C ground | |
| Mourning dove | *Zenaida macroura* | egg^2^ | | egg A ground, egg B ground | |
| Domestic duck | *Anas platyrhynchos* | egg^1^ | | egg A, egg B | |
| Baltimore oriole | *Icterus galbula* | bird^3^ | | breast, belly | |
| American Kestrel | *Falco sparverius* | bird^3^ | | breast, throat, spot | |
| Eastern bluebird | *Sialia sialis* | bird^3^ | | breast, belly, rump | |
| Northern cardinal | *Cardinalis cardinalis*, male | bird^3^ | | breast, throat | |
| Blackburnian warbler | *Setophaga fusca,* male | bird^3^ | | throat, belly, black streak | |
| Brown-headed cowbird | *Molothrus ater*, female | bird^3^ | | breast | |
| Indigo bunting | *Passerina cyanea,* male | bird^3^ | | belly, breast, throat | |
| American goldfinch | *Spinus tristis,* male | bird^3^ | | breast | |
| Banaquit | *Coereba flaveola* | bird^3^ | | belly, breast, throat | |
| Ruby-throated hummingbird | *Archilocus colubris,* male | bird^3^ | | tail, breast, throat | |
| European starling | *Sturnus vulgaris* | bird^3^ | | belly (green area), throat | |
| ^1.^ Commercially purchased. ^2.^ Abandoned egg collected under US Fish and Wildlife Service Collecting permit (MB81216C-3) and Virginia Department of Game and Inland Fisheries Scientific collecting permit (070605) ^3.^ Provided by the teaching collection at George Mason University | | | | | |
