# Supplementary material for: Recording animal-view videos of the natural world using a novel camera system and software package
Source: PLoS Biol. 2024 Jan 23;22(1):e3002444. doi: 10.1371/journal.pbio.3002444 (PMC10805291; doi:10.1371/journal.pbio.3002444)
Supplement: S16 Table — The mean absolute error associated with the reconstruction of the RAW values using Sony’s formula (JPG Conversion MAE) and the mean absolute error associated with the losses due to quantization and the lossy compression of the JPG algorithm. (DOCX) [file pbio.3002444.s028.docx]

| **Band** | **JPG Conversion MAE** | **Roundtrip MAE** |
| --- | --- | --- |
| Ultraviolet | 0.00256807 | 0.00245848 |
| Blue | 0.00129013 | 0.00103883 |
| Green | 0.00150668 | 0.00151699 |
| Red | 0.00081083 | 0.00098472 |
