# Supplementary figures and images for: Recording animal-view videos of the natural world using a novel camera system and software package
Source: PLoS Biol. 2024 Jan 23;22(1):e3002444. doi: 10.1371/journal.pbio.3002444 (PMC10805291; doi:10.1371/journal.pbio.3002444)

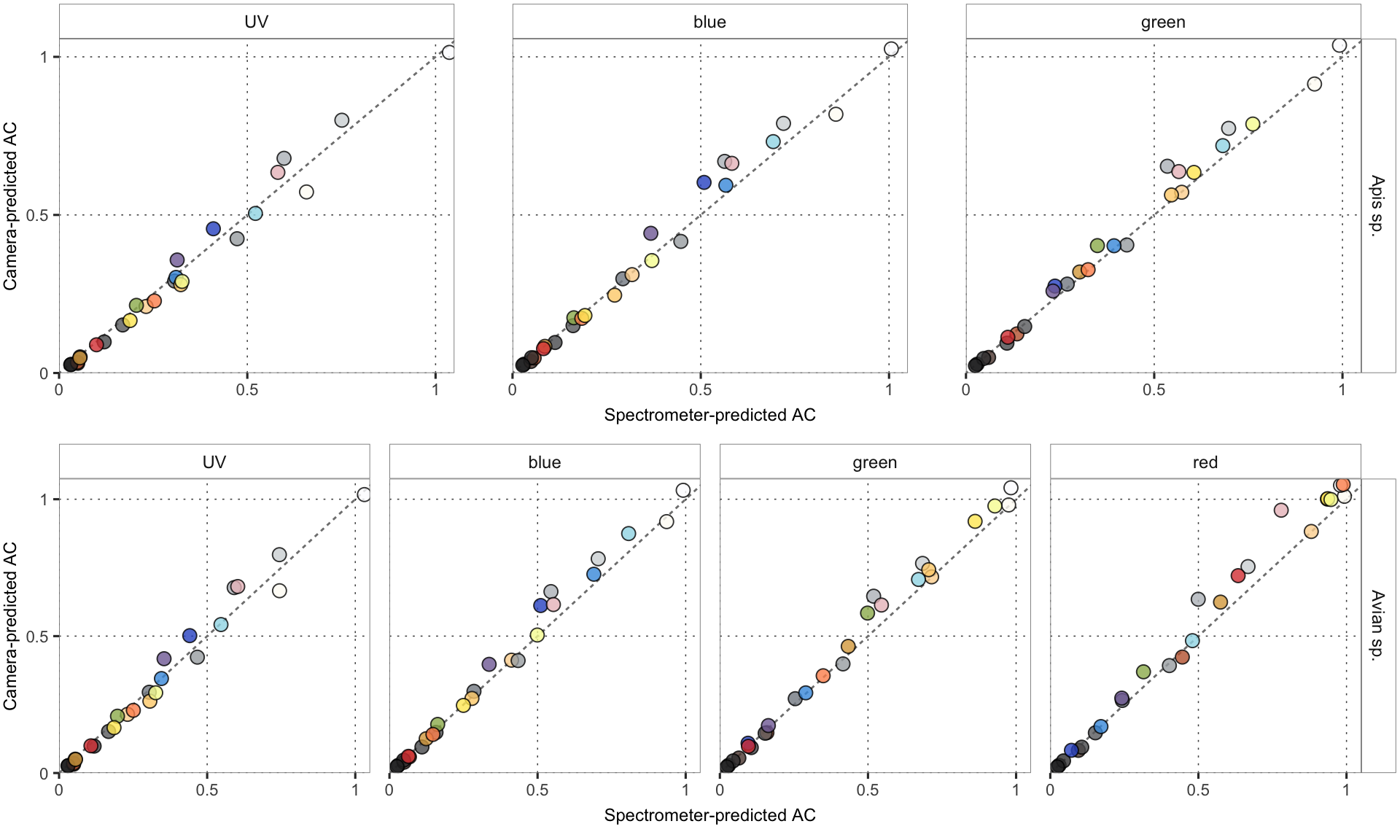

Supplement: S1 Fig — In this case, the videos were taken under full sunlight and normalized to a set of Spectralon standards. The plots show the animal quantum catch predicted from reflectance (Spectrometer-predicted AC) against our camera-predicted animal quantum catch (Camera-predicted AC). We plot the fit for known color standards (our custom ARUCO standard, another set of pastels and a DKK Color Calibration Chart), and for both the honeybee (Apis sp., top) and the average ultraviolet sensitive avian receiver (Avian sp., bottom), for each of their 3 and 4 photoreceptors, respectively. The marker colors indicate the human-perceived color of the sample. For data on fit, please see S1 Table. The data underlying this figure can be found in S1 Data. (TIF) [file pbio.3002444.s029.tif]

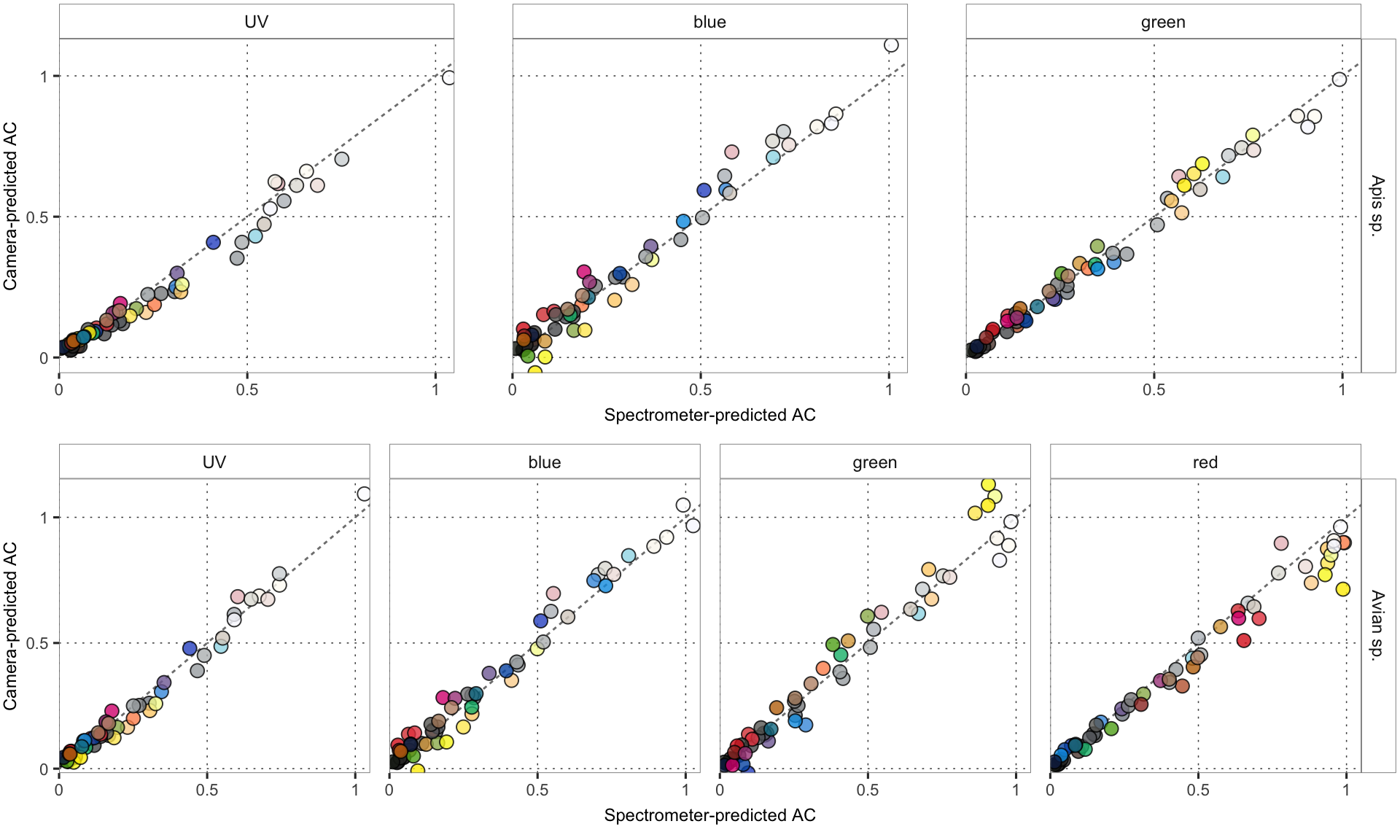

Supplement: S2 Fig — In this case, the images were taken under full sunlight and normalized to a set of Spectralon standards. The plots show the animal quantum catch predicted from reflectance (Spectrometer-predicted AC) against our camera-predicted animal quantum catch (Camera-predicted AC). We plot the fit for known color standards (our custom ARUCO standard, another set of pastels and a DKK Color Calibration Chart), and for both the honeybee (Apis sp., top) and the average ultraviolet sensitive avian receiver (Avian sp., bottom), for each of their 3 and 4 photoreceptors, respectively. The marker colors indicate the human-perceived color of the sample. For data on fit, please see S2 Table. The data underlying this figure can be found in S1 Data. (TIF) [file pbio.3002444.s030.tif]

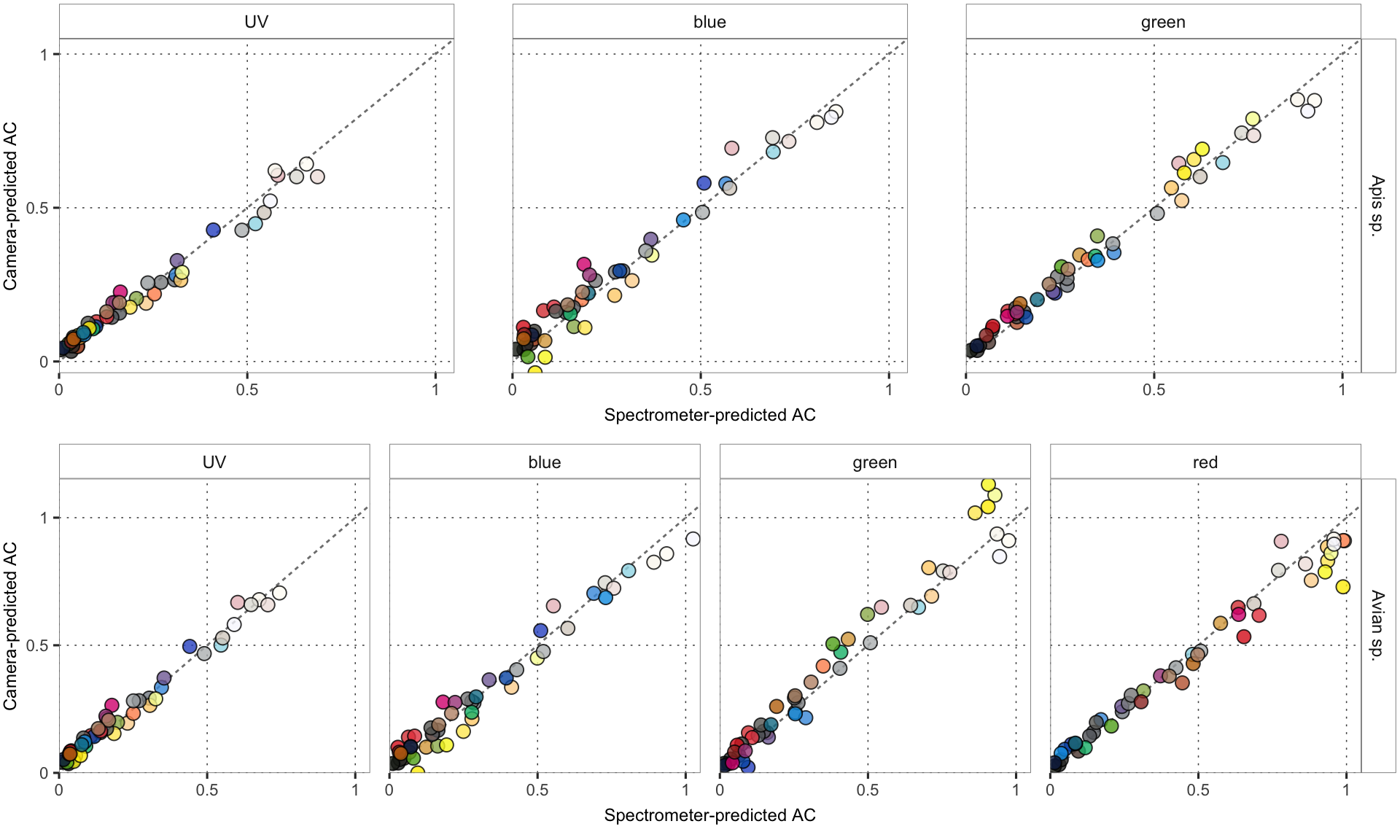

Supplement: S3 Fig — In this case, the images were taken under full sunlight and normalized to a set of ARUCO standards. The plots show the animal quantum catch predicted from reflectance (Spectrometer-predicted AC) against our camera-predicted animal quantum catch (Camera-predicted AC). We plot the fit for known color standards (our custom ARUCO standard, another set of pastels and a DKK Color Calibration Chart), and for both the honeybee (Apis sp., top) and the average ultraviolet sensitive avian receiver (Avian sp., bottom), for each of their 3 and 4 photoreceptors, respectively. The marker colors indicate the human-perceived color of the sample. For data on fit, please see S3 Table. The data underlying this figure can be found in S1 Data. (TIF) [file pbio.3002444.s031.tif]

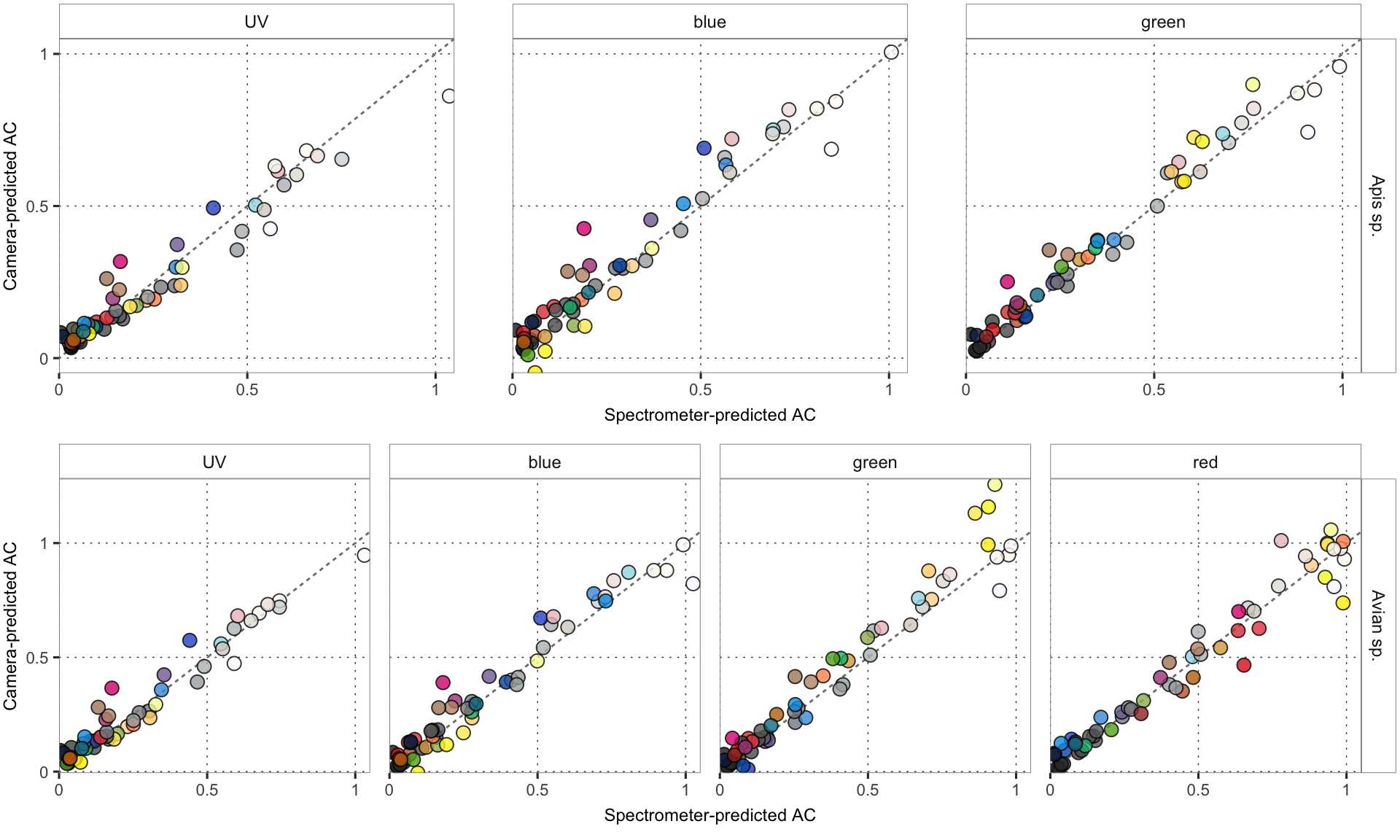

Supplement: S4 Fig — In this case, the images were taken under lab light and normalized to a set of Spectralon standards. The plots show the animal quantum catch predicted from reflectance (Spectrometer-predicted AC) against our camera-predicted animal quantum catch (Camera-predicted AC). We plot the fit for known color standards (our custom ARUCO standard, another set of pastels and a DKK Color Calibration Chart), and for both the honeybee (Apis sp., top) and the average ultraviolet sensitive avian receiver (Avian sp., bottom), for each of their 3 and 4 photoreceptors, respectively. The marker colors indicate the human-perceived color of the sample. For data on fit, please see S4 Table. The data underlying this figure can be found in S1 Data. (TIF) [file pbio.3002444.s032.tif]

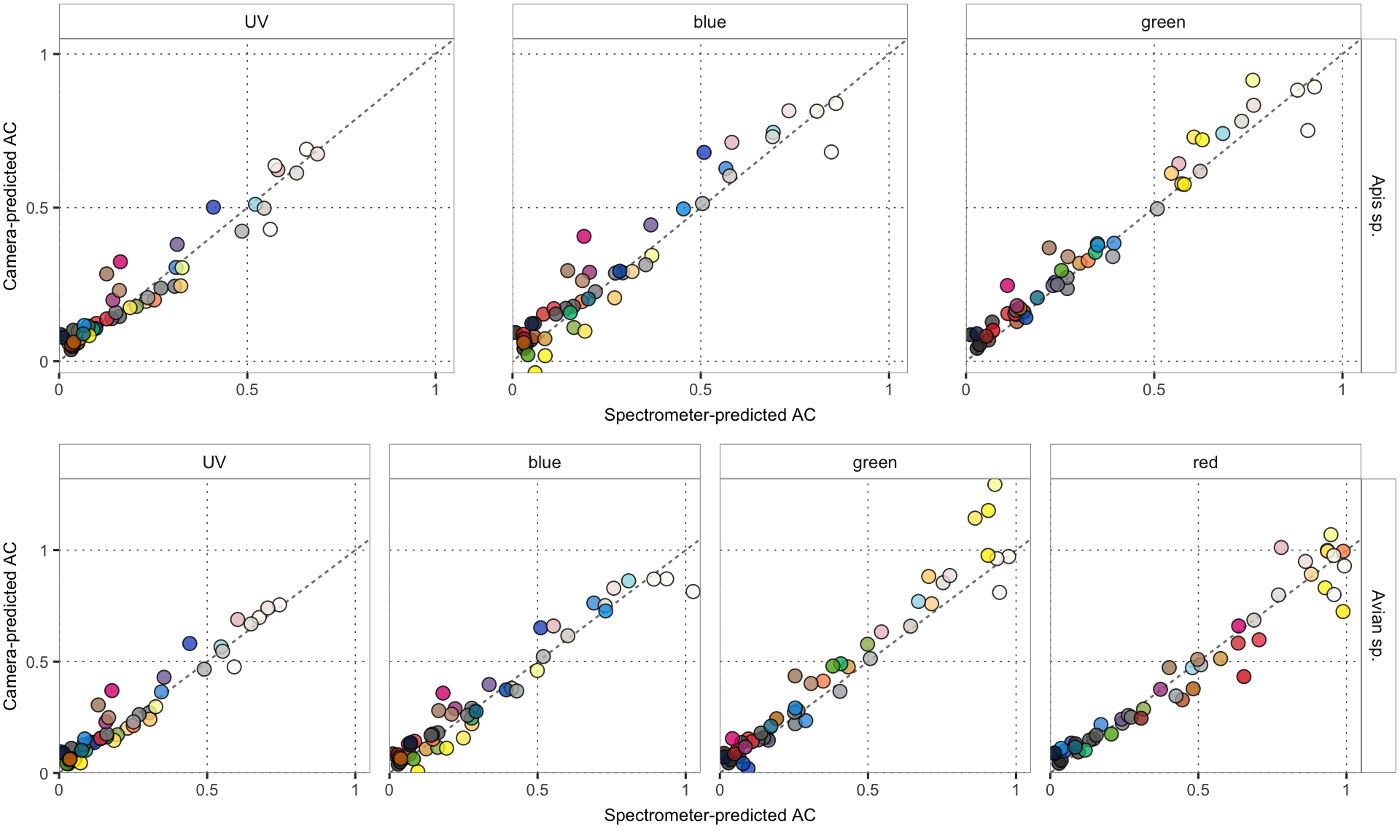

Supplement: S5 Fig — In this case, the images were taken under lab light and normalized to a set of ARUCO standards. The plots show the animal quantum catch predicted from reflectance (Spectrometer-predicted AC) against our camera-predicted animal quantum catch (Camera-predicted AC). We plot the fit for known color standards (our custom ARUCO standard, another set of pastels and a DKK Color Calibration Chart), and for both the honeybee (Apis sp., top) and the average ultraviolet sensitive avian receiver (Avian sp., bottom), for each of their 3 and 4 photoreceptors, respectively. The marker colors indicate the human-perceived color of the sample. For data on fit, please see S5 Table. The data underlying this figure can be found in S1 Data. (TIF) [file pbio.3002444.s033.tif]

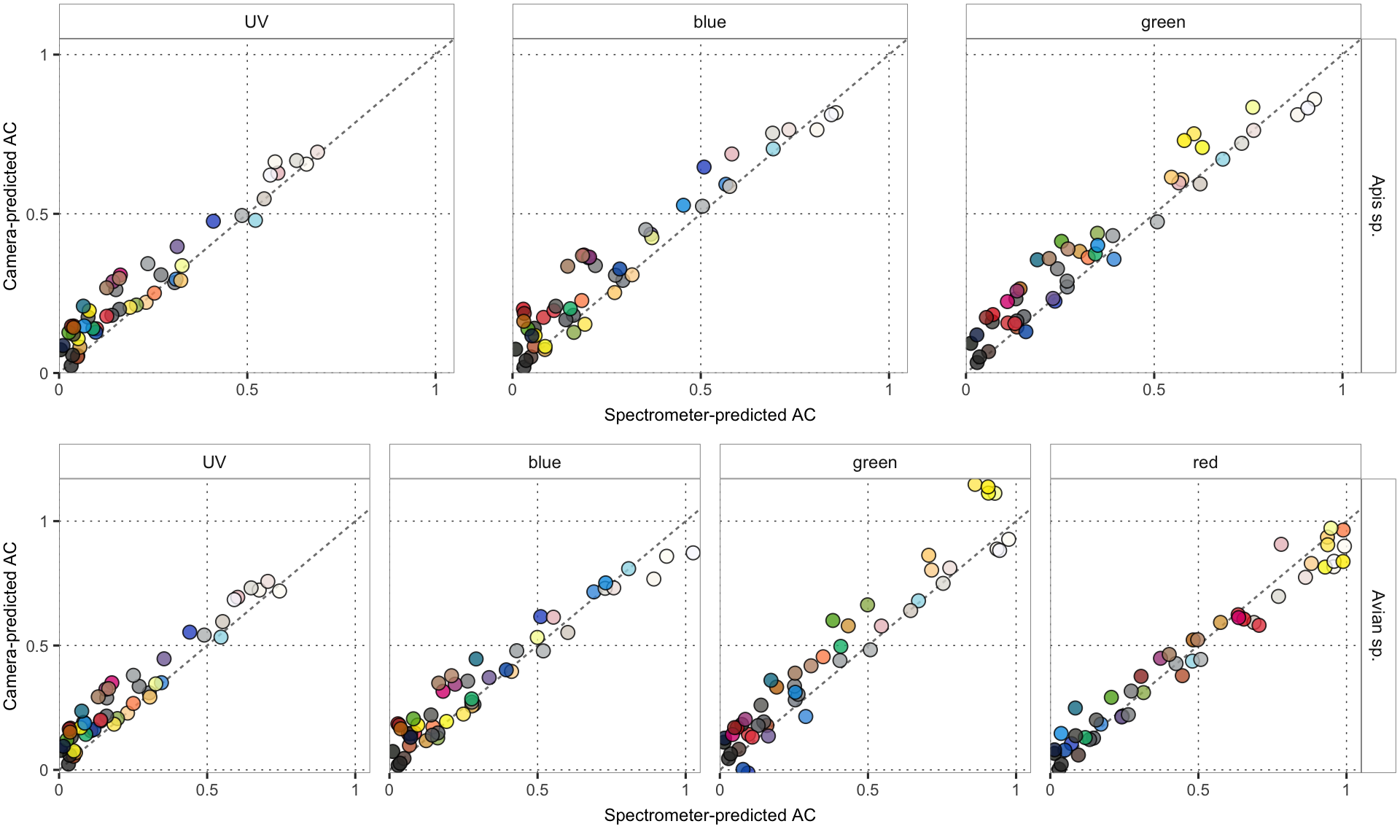

Supplement: S6 Fig — In this case, the videos were taken in shade outdoors and normalized to a set of ARUCO standards. The plots show the animal quantum catch predicted from reflectance (Spectrometer-predicted AC) against our camera-predicted animal quantum catch (Camera-predicted AC). We plot the fit for known color standards (our custom ARUCO standard, another set of pastels and a DKK Color Calibration Chart), and for both the honeybee (Apis sp., top) and the average ultraviolet sensitive avian receiver (Avian sp., bottom), for each of their 3 and 4 photoreceptors, respectively. The marker colors indicate the human-perceived color of the sample. For data on fit, please see S6 Table. The data underlying this figure can be found in S1 Data. (TIF) [file pbio.3002444.s034.tif]

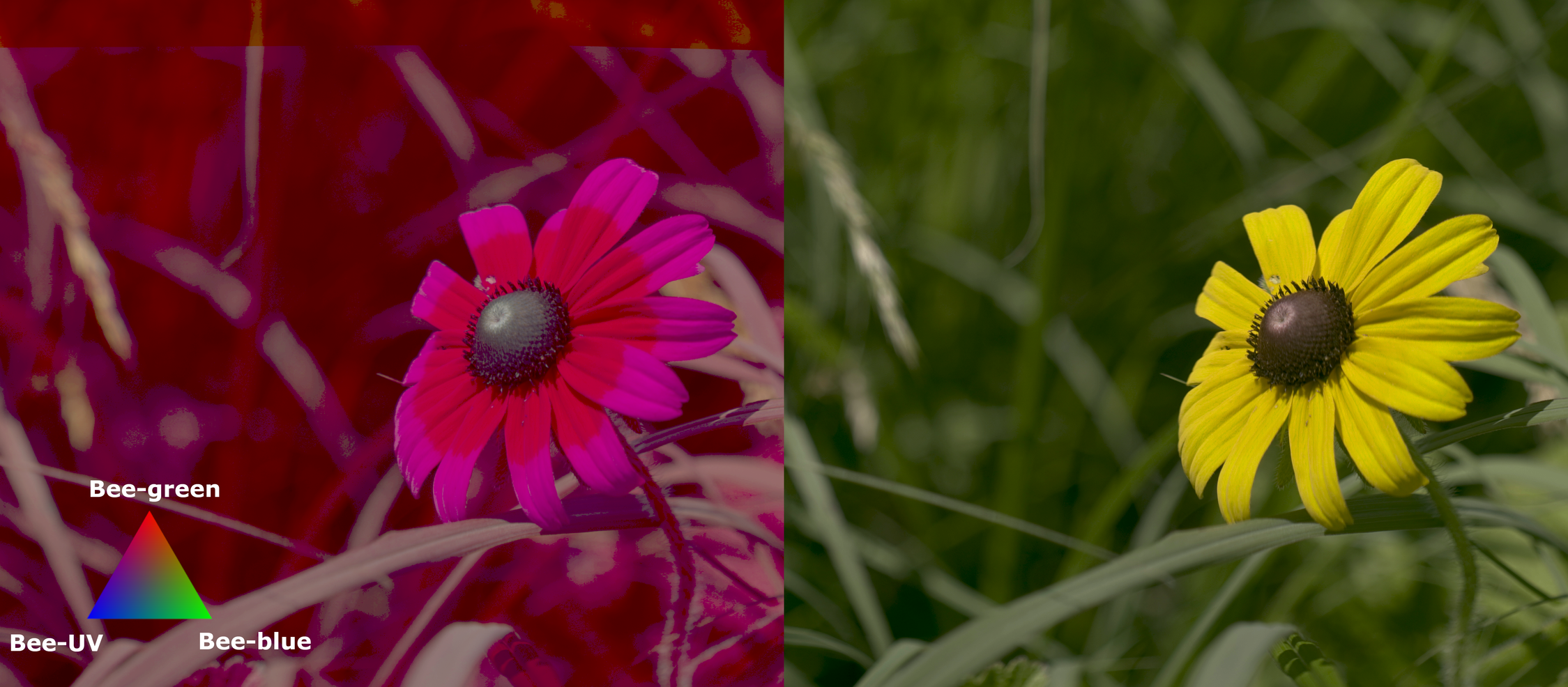

Supplement: S7 Fig — A black-eyed Susan (Rudbeckia hirta) depicted as a honeybee (Apis mellifera) false color image and the same flower in a human-vision. In honeybee false color images, the blue, green, and red channels represent quantum catches of their UV-, blue-, and green-sensitive photoreceptors, respectively. We provide a visual key (bottom left corner) illustrating each of the bee’s 3 photoreceptors (vertices) and the colors used to represent the variable stimulation of these 3 photoreceptors (interior colors). This flower has a nectar guide that aids recruitment [43]. To our eye, the black-eyed Susan appears entirely yellow because it reflects primarily long-wavelength light in the human-visible range. Whereas in the bee false color image, the distal petals appear magenta because they reflect UV in addition to long-wavelength light, stimulating both the photoreceptors sensitive to UV (depicted as blue) and those sensitive to green light (depicted as red). By contrast, the central portion of the petals does not reflect UV and therefore appears red. We applied a gamma correction to the honeybee false color and linear (human) image for display purposes (AC0.3 and CC0.5). These are the same images as shown in Fig 1. (TIF) [file pbio.3002444.s035.tif]

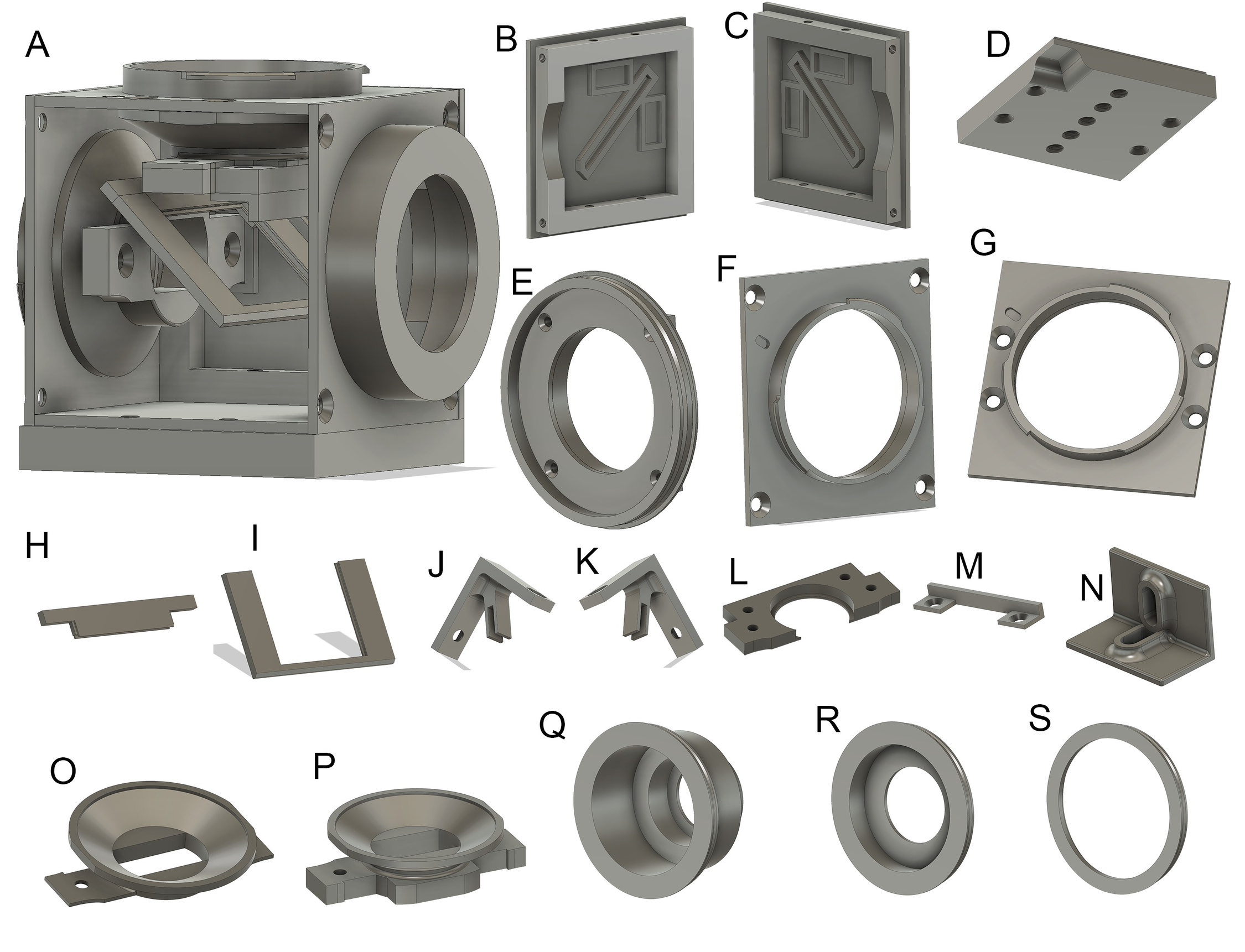

Supplement: S8 Fig — (A) Schematic showing the assembled cage, with mounting points for the (i) visible light Sony camera, the (ii) full-spectrum modified camera. The complete set of parts are as follows: (B) Side 2, (C) Side 1, (D) Base, (E) Bellows Cube Face, (F) Rear Camera Face, (G) Top Camera Face, (H) Mirror Latch, (I) Mirror Mount, (J) Internal Bracket, (K) Internal Bracket (mirrored), (L) UV Bandpass Filter Holder, (M) Internal Shroud, (N) Camera Base Connector, (O) UV Bandpass Cone, (P) Visible Cone Baffle, and 3 options for Bellows Lens Mounts, for mounting (Q) 80 mm f5.6, (R) 135mm f5.6, or (S) 210 mm f5.6 Nikon EL-Nikkor Enlarging lenses. We printed the parts using a Prusa i3 MKS+ 3D printer, with and without the MMU2s+, from black PLA and PETG filament (Hatchbox) with 0.15 mm QUALITY presets in PrusaSlicer. Higher quality prints are recommended for regular field use. The STL files of all the parts required to print a system, along with a complete bill of materials and detailed instructions for printing and assembly, are freely available from the Gitlab Repository https://gitlab.com/multispectrum-beamsplitter. We have also included a set of 3MF files that can be opened with the free PrusaSlicer software. These files illustrate the recommended print orientation of each file and highlight the few parts that require supports. Future updates will be provided via the repository as the design continues to evolve and improve. (TIF) [file pbio.3002444.s036.tif]

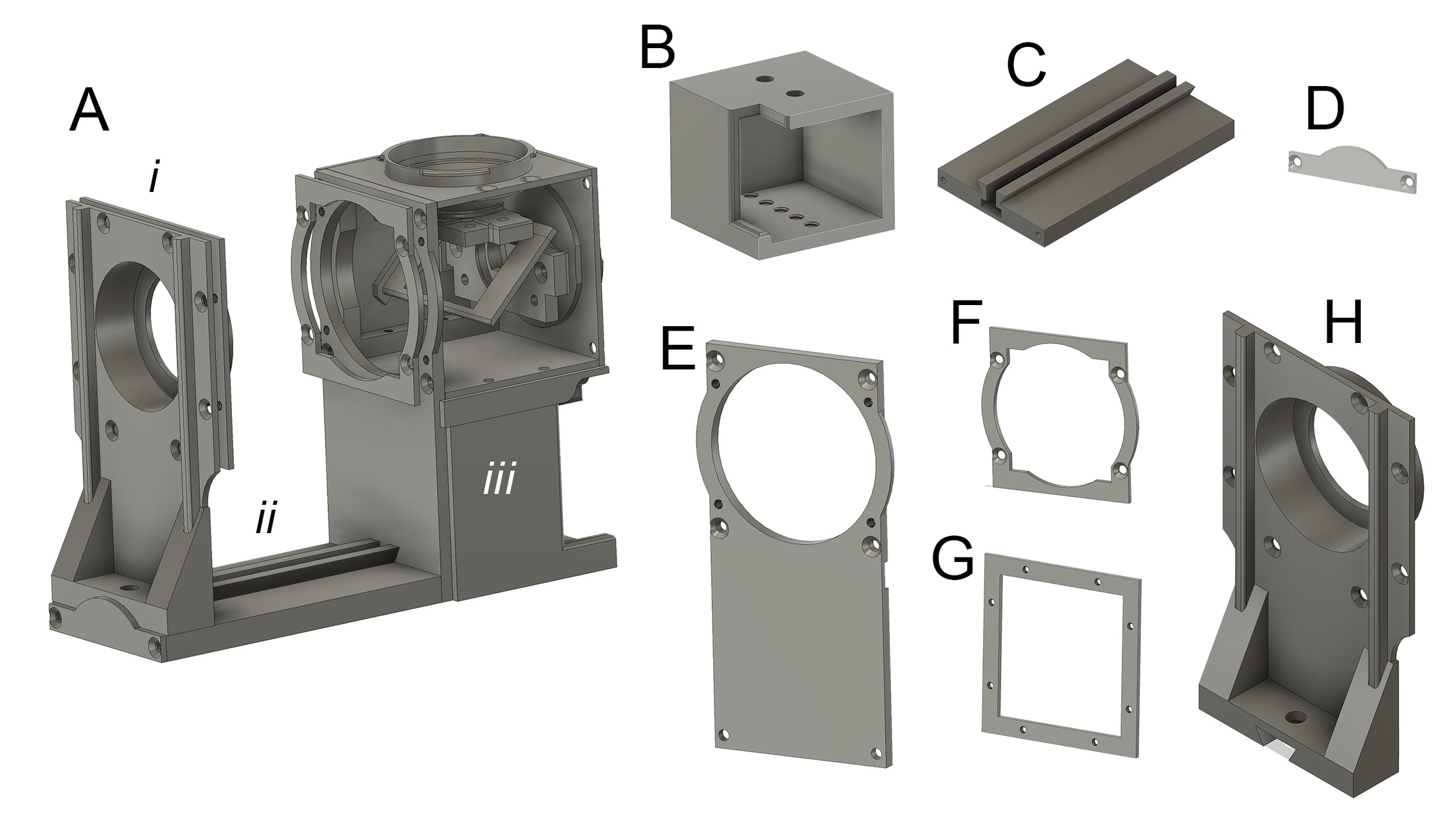

Supplement: S9 Fig — We recommend using commercially available bellows such as the Novoflex BALLPRO due to their robustness, but provide plans for a DIY option as an alternative. (A) Schematic showing the assembled DIY sliding bellow system, attached to the housing, with a (i) lens and bag bellows attachment plate, (ii) rail to allow for focusing, and a (iii) spacer. The complete sets of parts are as follows: (B) Base Extension, (C) Rail, (D) Rail End Stop, (E) Cube Face, (F) Bag Bellows Cube Mount, (G) Bag Bellows Lens Mount, (H) Sliding Plate. In addition, the DIY bellows require semi stiff fabric that is light tight (e.g., faux black leather). The fabric was cut using a laser and then assembled with contact cement. We printed the parts using a Prusa i3 MKS+ 3D printer, with and without the MMU2s+, from black PLA (Hatchbox) with 0.15 mm QUALITY presets in PrusaSlicer. The files and information to print the system is available from the Gitlab Repository https://gitlab.com/multispectrum-beamsplitter. (TIF) [file pbio.3002444.s037.tif]

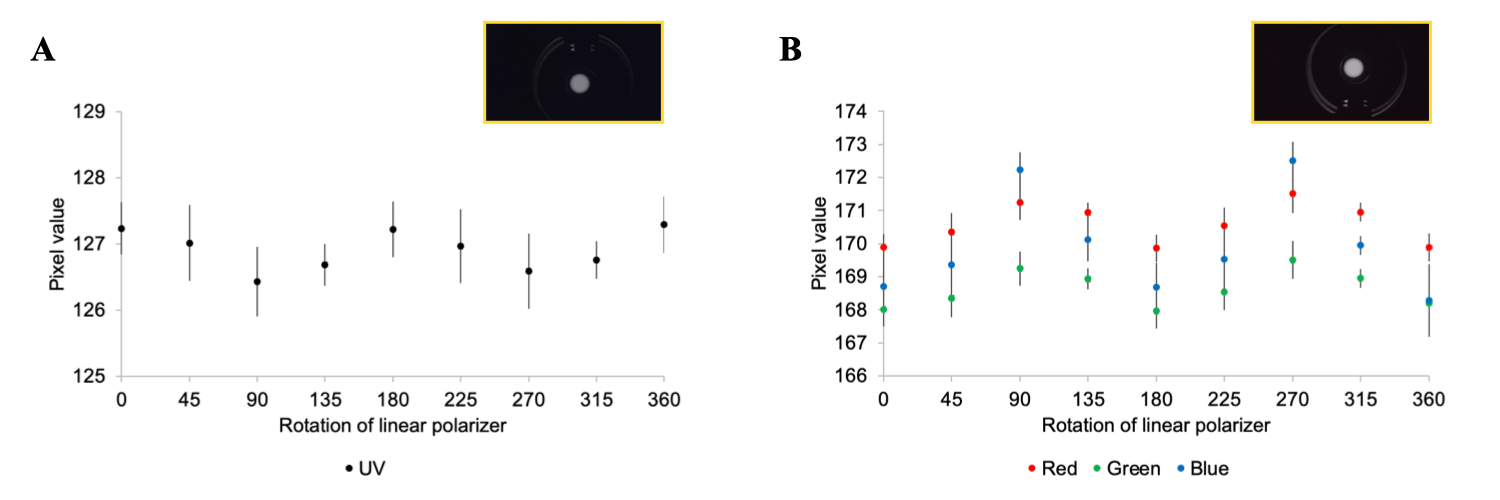

Supplement: S10 Fig — Fluctuations in the pixel values from the UV (A) and the visible camera (B) as a function of the polarization plane of the illuminating light. Photos were taken of the exit port of an integrating sphere (StellarNet IC2), illuminated with broadband light (full-spectrum xenon bulb, Thorlabs, SLS 202 through 1,000 μm fiber optics, Edmund Optics, 58–458), through a polarization filter positioned at varying rotational angles. Example photos are shown in the insets. UV light with a polarization plane of 90°, i.e., horizontally polarized, is less likely to be reflected by the beam splitter than vertically polarized UV light, and up to 0.6% of horizontally polarized light may be blocked in the UV channel. On the other hand, visible light with a polarization plane of 0°, i.e., vertically polarized light, is less likely to be transmitted by the beam splitter than horizontally polarized visible light, and up to 2.4, 0.8, and 1.0% of the polarized light may be blocked in the blue, green, and red channels. The effect is too small (approximately 1 to 2 pixel value) to interfere with the intended use of the camera; however, care must be taken if the system is adapted for recording polarized light. The data underlying this figure can be found in S1 Data. (TIF) [file pbio.3002444.s038.tif]

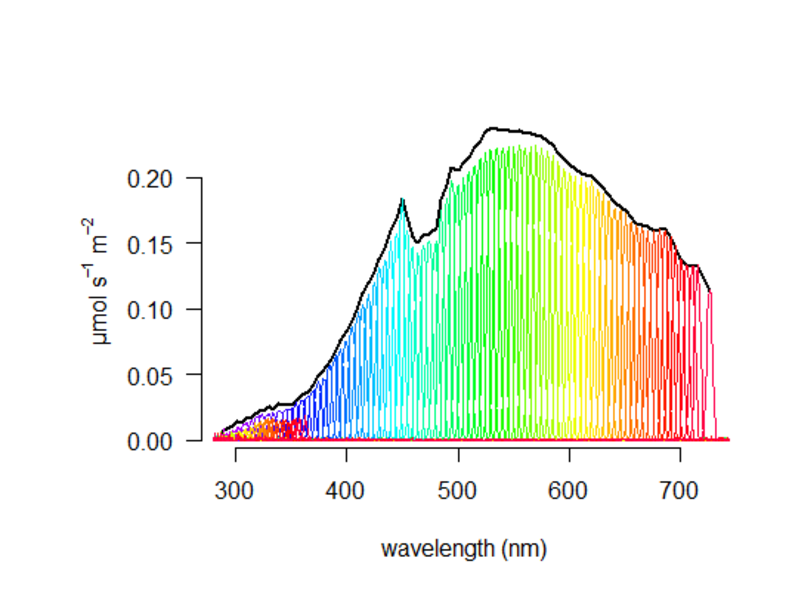

Supplement: S11 Fig — We used a xenon light source (SLS 205, Thorlabs) connected to a monochromator (Optimetrics, DMC1-03) to deliver narrow bands of light (colored bands, mean FWHM ± s.e. = 7.3 ± 0.29 nm) from 280 nm to 800 nm. The total power used for estimation (solid black line) was the sum photon flux (μmol s−1 m−2). To depict summed data (solid black line) and individual spectra (colorful) plots on the same display, individual spectra were multiplied by 6.5. Second order scatter is visible from approximately 300 nm to 350 nm as smaller orange to red peaks when illuminated with light from 600 nm to 700 nm. These were removed (see Materials and methods) and we replicated these camera spectral sensitivity measurements using another approach that omitted second order scatter using longpass filters (for more details, see Method A in S1 Text). The data underlying this figure can be found in S1 Data. (TIF) [file pbio.3002444.s039.tif]

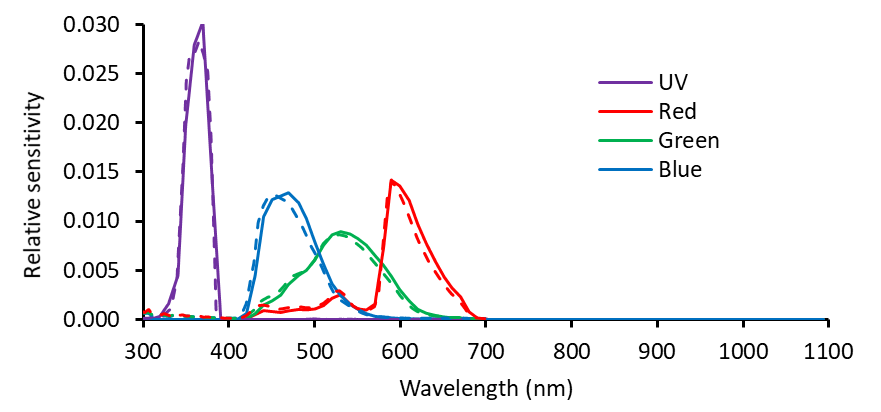

Supplement: S12 Fig — Relative camera sensor sensitivity for the ultraviolet (purple lines), blue (blue lines), green (green lines), and red (red lines) sensors, measured from 300 nm to 1,100 nm (solid) or 300 nm to 700 nm (dashed). Using longpass filters, we excluded second order scatter over the extended range (for details, see Method A in S1 Text) and confirmed these effects did not impact our readings over the reduced range (see Materials and methods). To ensure the data were comparable, we relativized both curves over the 300 nm to 700 nm range. For the extended range (solid) sensitivities above 700 nm were negligible for the ultraviolet, blue, green, and red sensors. The data underlying this figure can be found in S1 Data. (TIF) [file pbio.3002444.s040.tif]

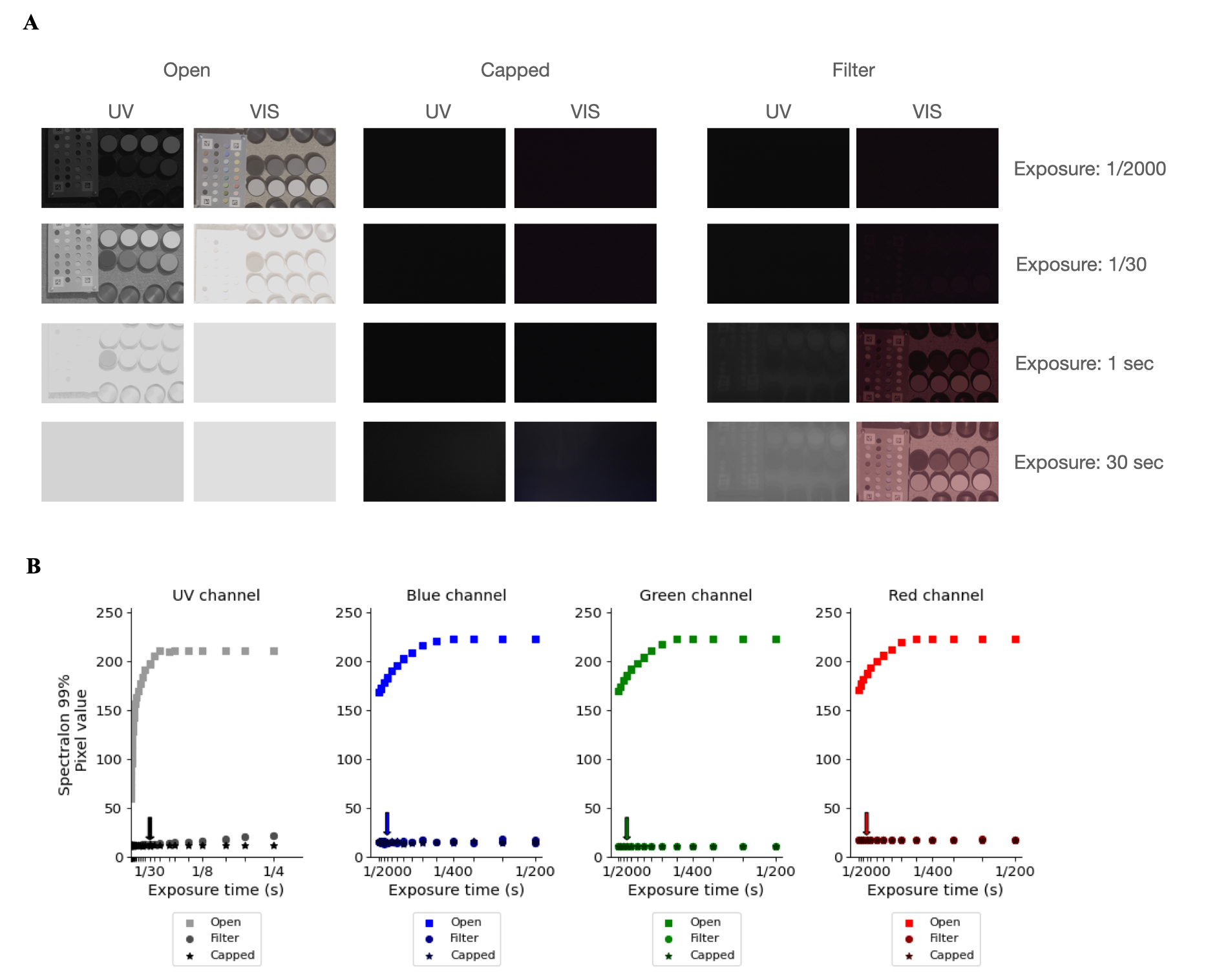

Supplement: S13 Fig — We photographed a set of 8 Spectralon standards under direct sunlight with abundant IR light (the total irradiance measured in μWatt/cm2 in the infrared, 700 nm to 1,100 nm, spectrum was 5 to 6 times higher than in the ultraviolet, 300 nm to 400 nm, spectrum). We set both cameras at the same shutter speeds (1/4,000) and sequentially increased this a stop for each camera up to shutter speeds of 30 s. Photos were taken under 3 conditions: as normal (“open” condition), with the camera cap on to quantify dark noise (“capped” condition), while in the third, “filter” condition we placed a set of 2 longpass filters directly in front of the lens (MidOpt LP715-25 and a Semrock BLP01-532R-25) that collectively blocked all light other than near infrared. (A) Representative photos at 4 exposure speeds show that both the UV and the visible camera are capable of registering infrared light. (B) The pixel values of the white Spectralon standard (99% reflective up to 2,300 nm) show that the intended UV and VIS image saturate before the IR signal becomes detectable. At the shutter speeds required for UV photography (indicated by arrows) the infrared contamination is statistically indistinguishable from dark noise (comparison of pixel values from the “capped” condition vs. from the “filter” condition by a Z-test: UV: p = 0.743; blue: p = 0.960; green: p = 0.841; red: p = 0.985). The data underlying this figure can be found in S1 Data. (TIF) [file pbio.3002444.s041.tif]

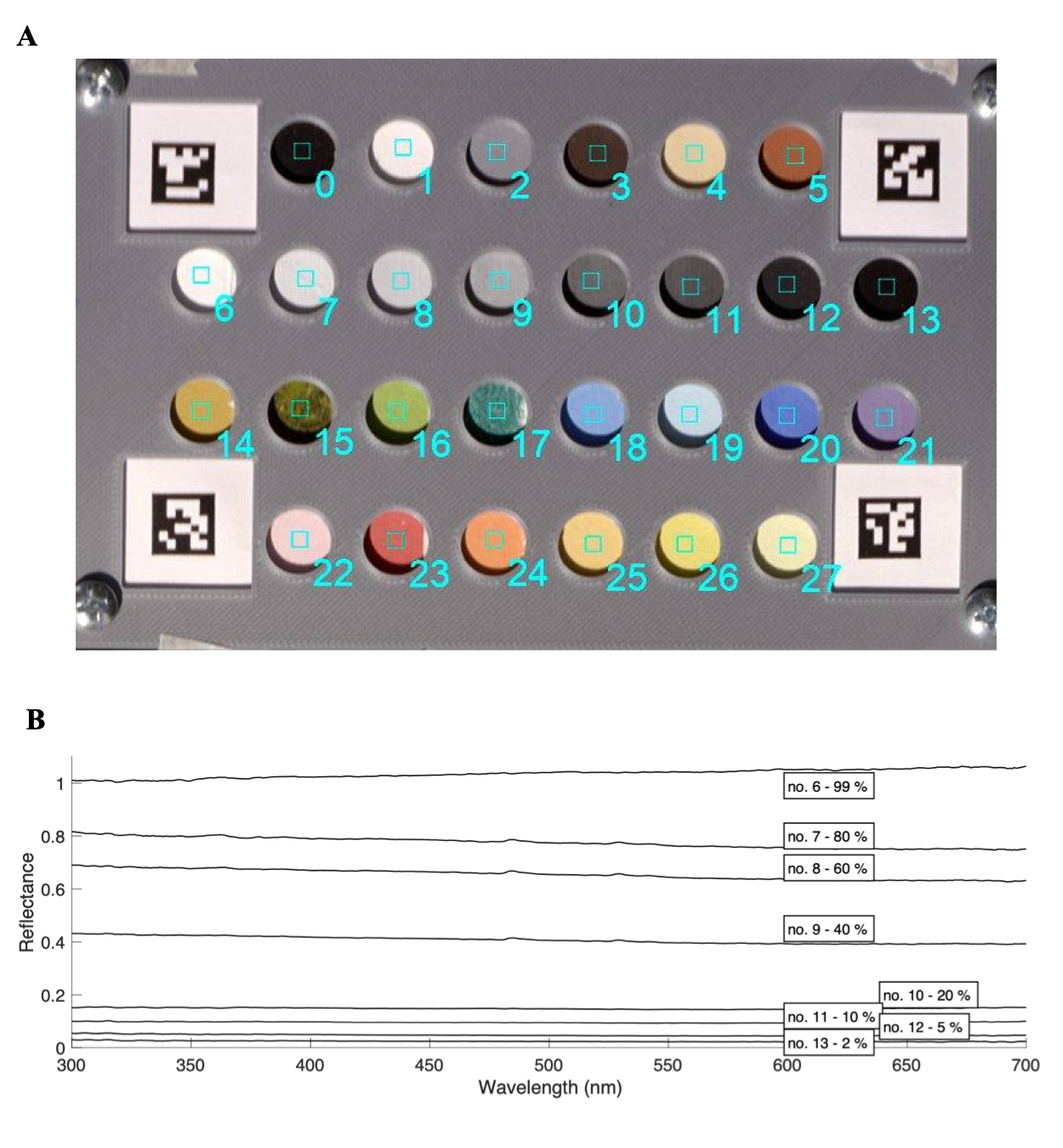

Supplement: S14 Fig — We used a custom color card (A) that featured 4 ARUCO fiducial markers and 28 distinct colors. The color patches were made from pastels (positions 0–5 and 14–27) or a mixture of barium sulfate (white) and flat black (Black 3.0) paints (positions 6–13) that are isoluminant across the 300 nm to 700 nm wavelength range (B). The use of the custom color standard enables fast, highly accurate, automated methods for calibration, normalization, and transformation. For details on how to construct these cards, see S8 Table. The data underlying this figure can be found in S1 Data. (TIF) [file pbio.3002444.s042.tif]

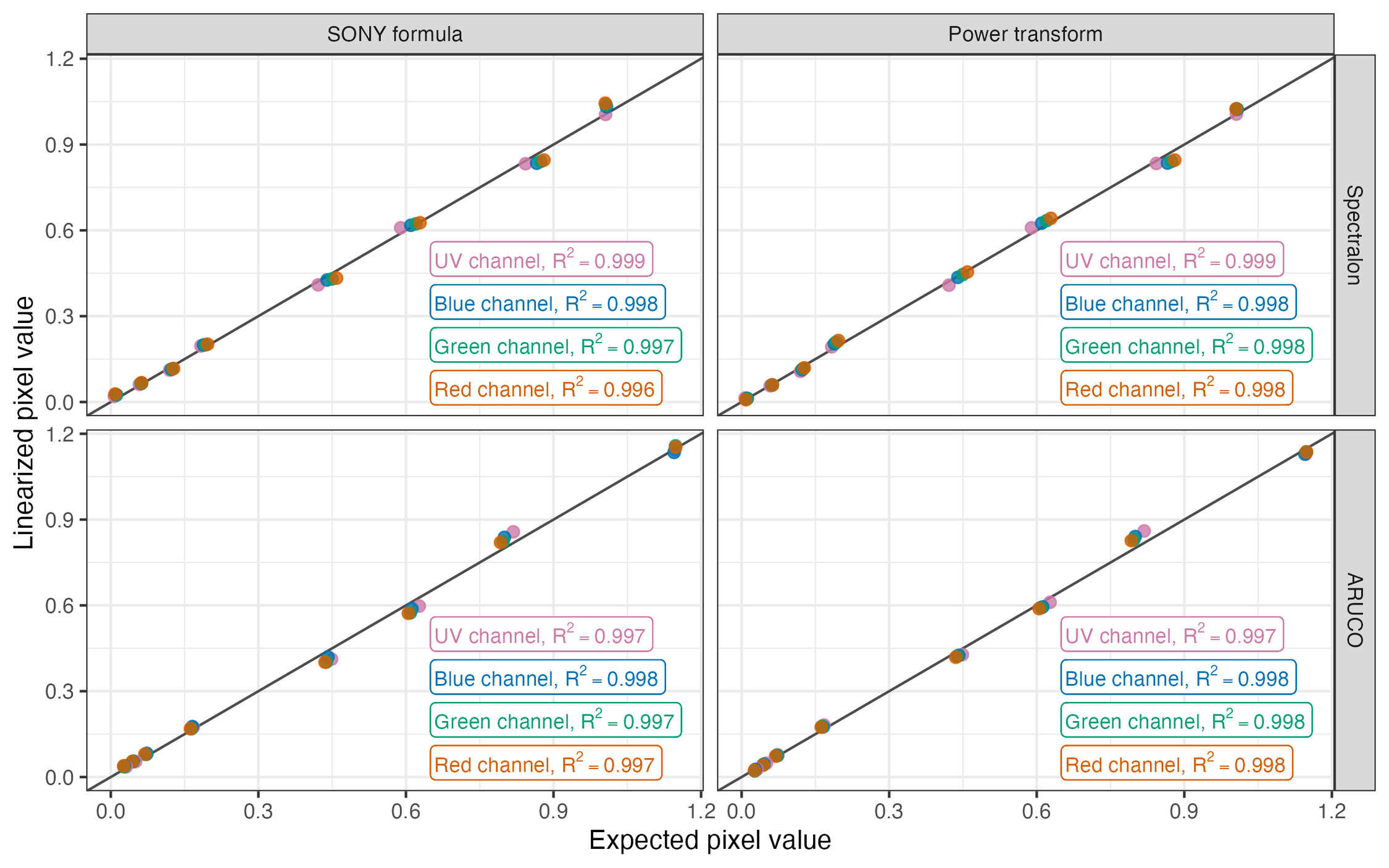

Supplement: S15 Fig — The linearization is highly accurate, regardless of whether the grayscale standards are Spectralon standards or the Barium-sulfate mixes on our ARUCO custom color card, and whether the exact Sony formula or an approximate power transform is used. The data underlying this figure can be found in S1 Data. (TIF) [file pbio.3002444.s043.tif]

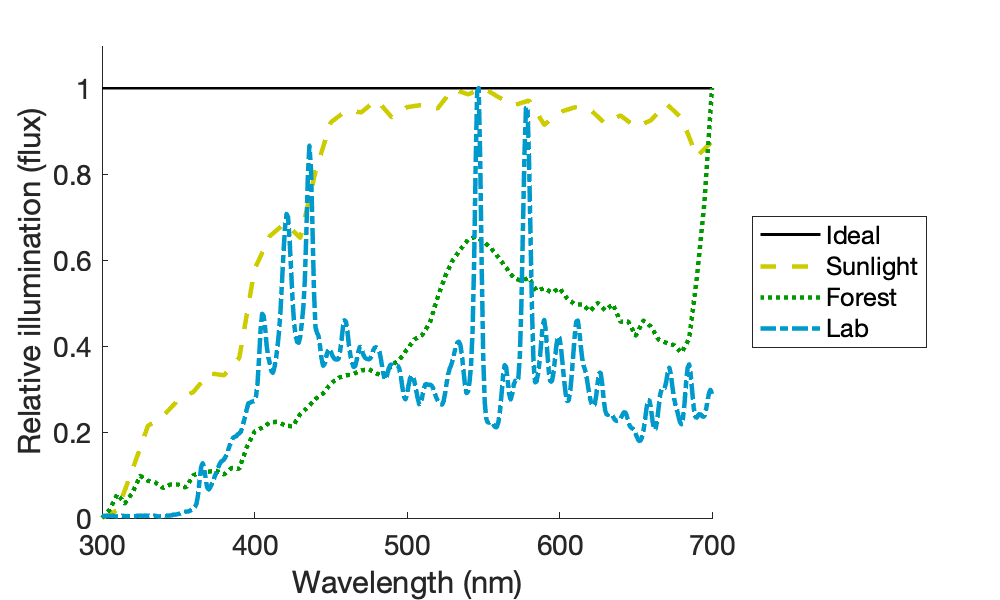

Supplement: S16 Fig — “Ideal” refers to the isoluminant illumination, “sunlight” to the standard d65 illumination, “forest” was taken from the PAVO package, and “lab” is the metal halide lamp in our lab. The data underlying this figure can be found S1 Data. (TIF) [file pbio.3002444.s044.tif]

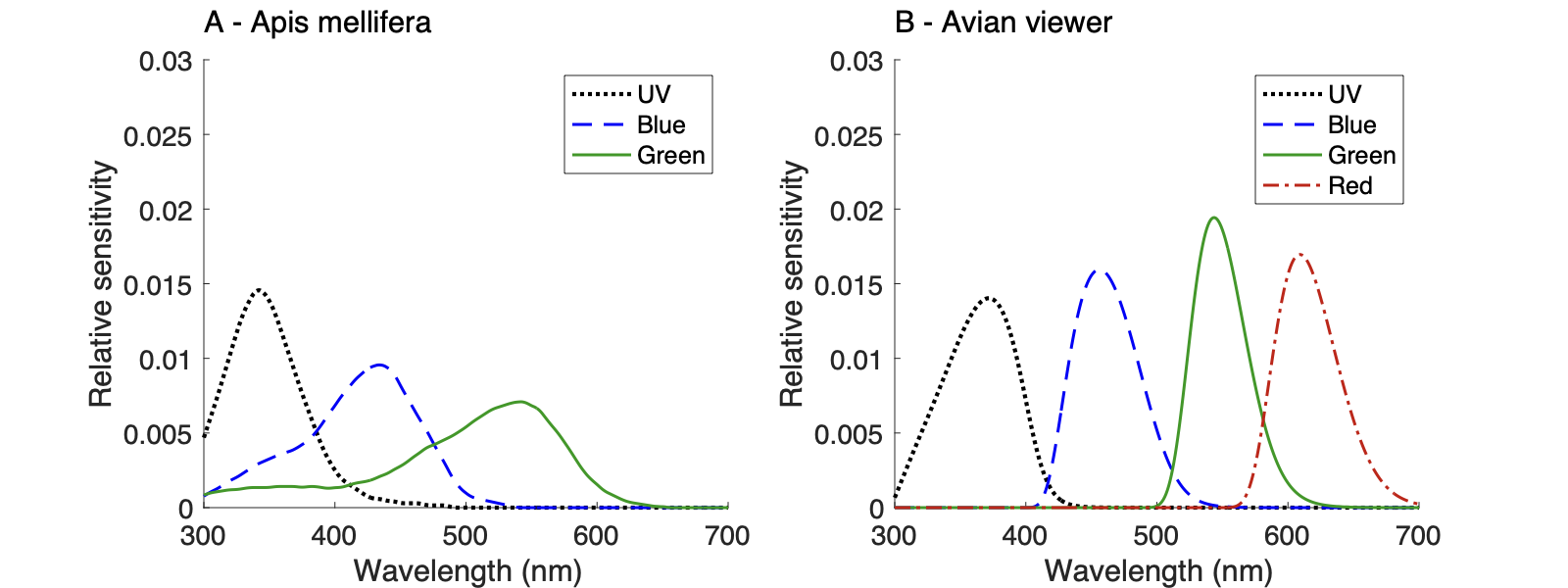

Supplement: S17 Fig — The photoreceptors have peak sensitivities in the ultraviolet (dotted black line), blue (dashed blue line), green (green line), and red (dot-dashed red line) parts of the spectrum. The data underlying this figure can be found in S1 Data. (TIF) [file pbio.3002444.s045.tif]

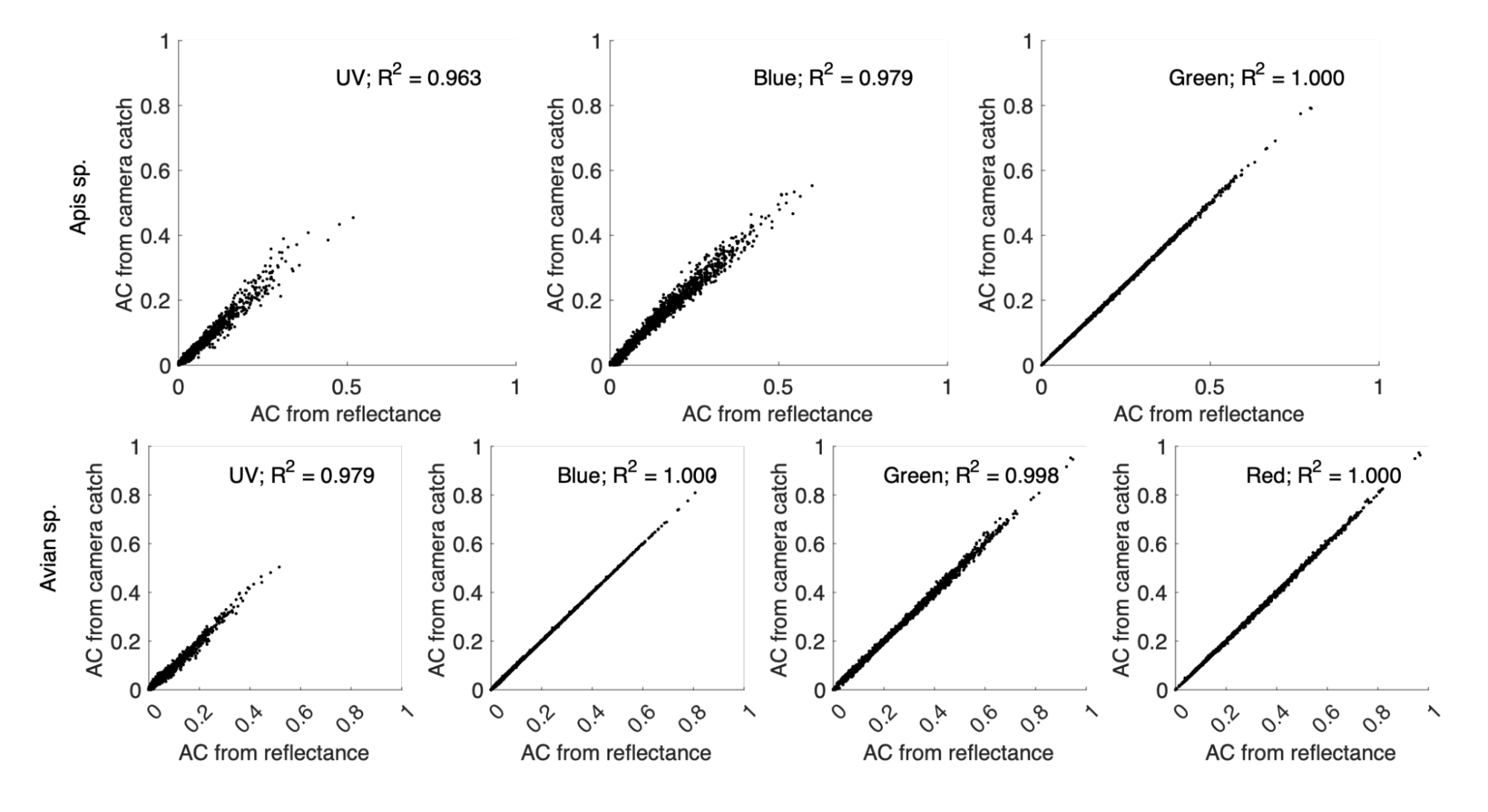

Supplement: S18 Fig — The figure shows the relationship between the photoreceptor quantum catches calculated directly from reflectances (AC from reflectance) vs. estimated from camera catches with the transformation matrix (AC from camera catch). In this paper, we used 2,494 spectra from the FReD database [59] to derive a transformation matrix, T that converts linear camera catches to animal catches. Here, we illustrate the fit for those relationships. R2 values for the full library are shown (rather than the testing library used to test accuracy). The data underlying this figure can be found in S1 Data. (TIF) [file pbio.3002444.s046.tif]

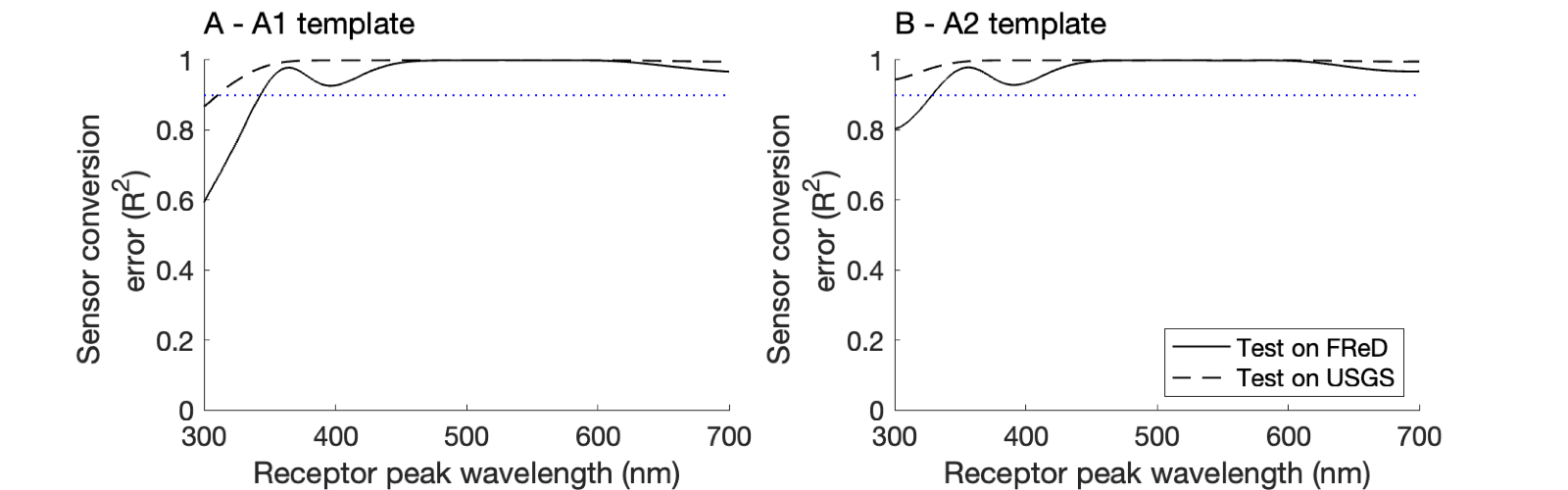

Supplement: S19 Fig — The figure shows the R2 values of the fit between the photoreceptor quantum catches calculated directly from reflectances vs. estimated from camera catches with the transformation matrix. The fit was evaluated using a set of reflectances reserved for testing from the FReD (solid lines) and on the USGS Spectral Library (dashed lines), for a synthetically generated library of photoreceptors with peak sensitivities between 300 nm and 700 nm, based on the A1 (A) and A2 templates (B) from [60]. The performance is reliably R2 > 0.90 (blue dotted line), in most cases exceeding 0.99, for all wavelengths between 343 nm and 700 nm. The system only becomes inaccurate at the extreme end of the ultraviolet, past 340 nm, for which the ultraviolet camera has little sensitivity. This suggests that any organism with peak photoreceptor sensitivities falling in this range (the “effective range” of the camera) should produce accurate results. Note however that the A1 and A2 templates used above are approximations, and the precise fit for a particular organism will depend on the exact shape of its photoreceptors’ sensitivities. For example, the sensitivity of the honeybee’s UV receptor is narrower and less sensitive to far-UV than the template, and so its fit is better than predicted here (R2 = 0.958 when testing on FReD, R2 = 0.991when testing on USGS, see Tables S10 and S12). The data underlying this figure can be found in S1 Data. (TIF) [file pbio.3002444.s047.tif]

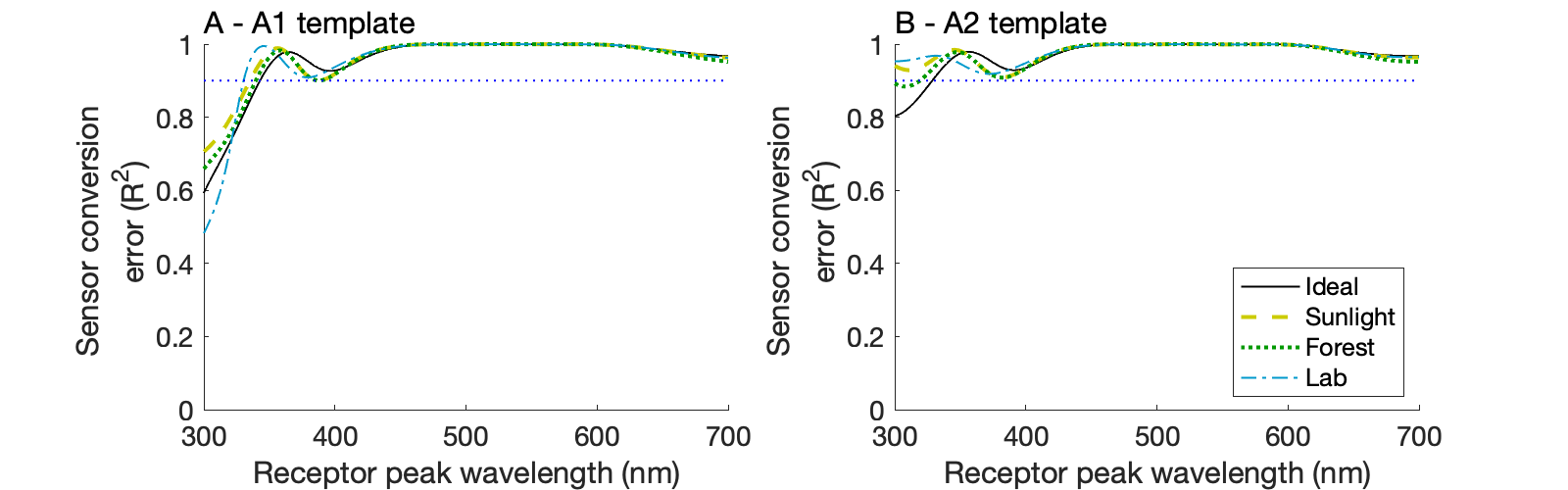

Supplement: S20 Fig — The figure shows the R2 values of the fit between the photoreceptor quantum catches calculated directly from reflectances vs. estimated from camera catches with the transformation matrix. The fit was evaluated using a set of reflectances reserved for testing from the FReD, for a synthetically generated library of photoreceptors with peak sensitivities between 300 nm and 700 nm, based on the A1 (A) and A2 templates (B) from [60], for 4 viewing illuminations: ideal (black solid line), direct sunlight (yellowish green dashed line), forest shade (green dotted line), and the metal halide lamp in the lab (blue dot-dashed line). The performance between 343 nm to 700 nm is reliably R2 > 0.90 (blue dotted line), in most cases exceeding 0.99, for all cases tested, indicating that the method is not sensitive to the viewing illumination. The data underlying this figure can be found in S1 Data. (TIF) [file pbio.3002444.s048.tif]

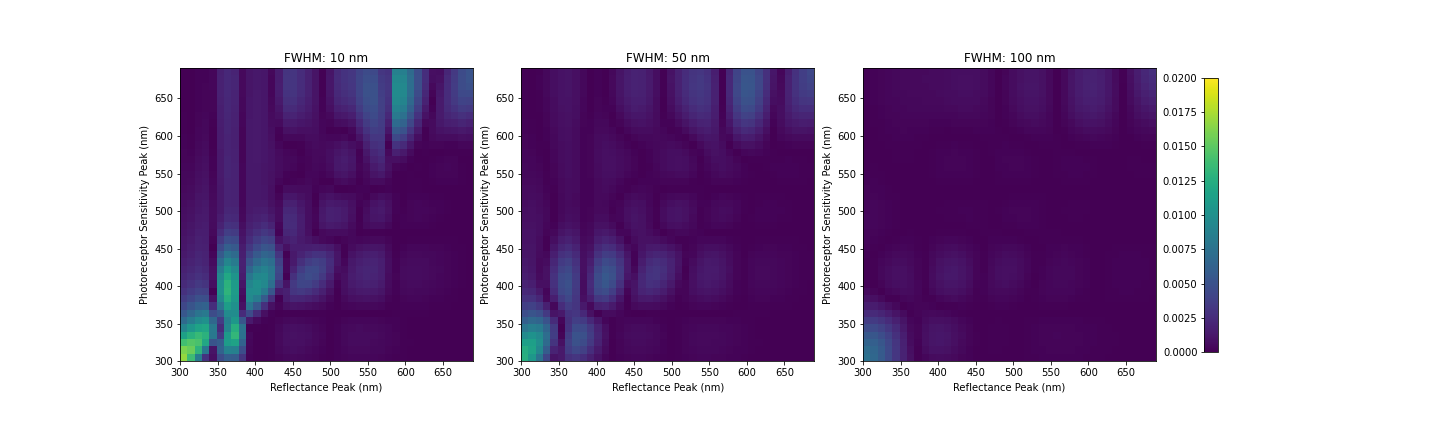

Supplement: S21 Fig — The absolute error of the transformation is shown as function of the peak reflectance (x axis) and the receptor’s peak sensitivity (y axis), for reflectance spectra with 3 different values of full-width-at-half-maximum (FWHM). In this analysis, we used simulated reflectance spectra, assumed to follow a Gaussian distribution, and the standard photoreceptor template from [60]. The performance remains reasonable (<0.017 absolute error) even for hypothetical materials with reflectance spectra that have a full-width-at-half-maximum of 10 nm. Minor errors appear when estimating the response of a short wavelength sensitive receptor (peak sensitivity <400 nm) to very narrow emissions in the far-UV (<340 nm) and around approximately 390 nm where the camera has a sensitivity gap. The data underlying this figure can be found in S1 Data. (TIF) [file pbio.3002444.s049.tif]

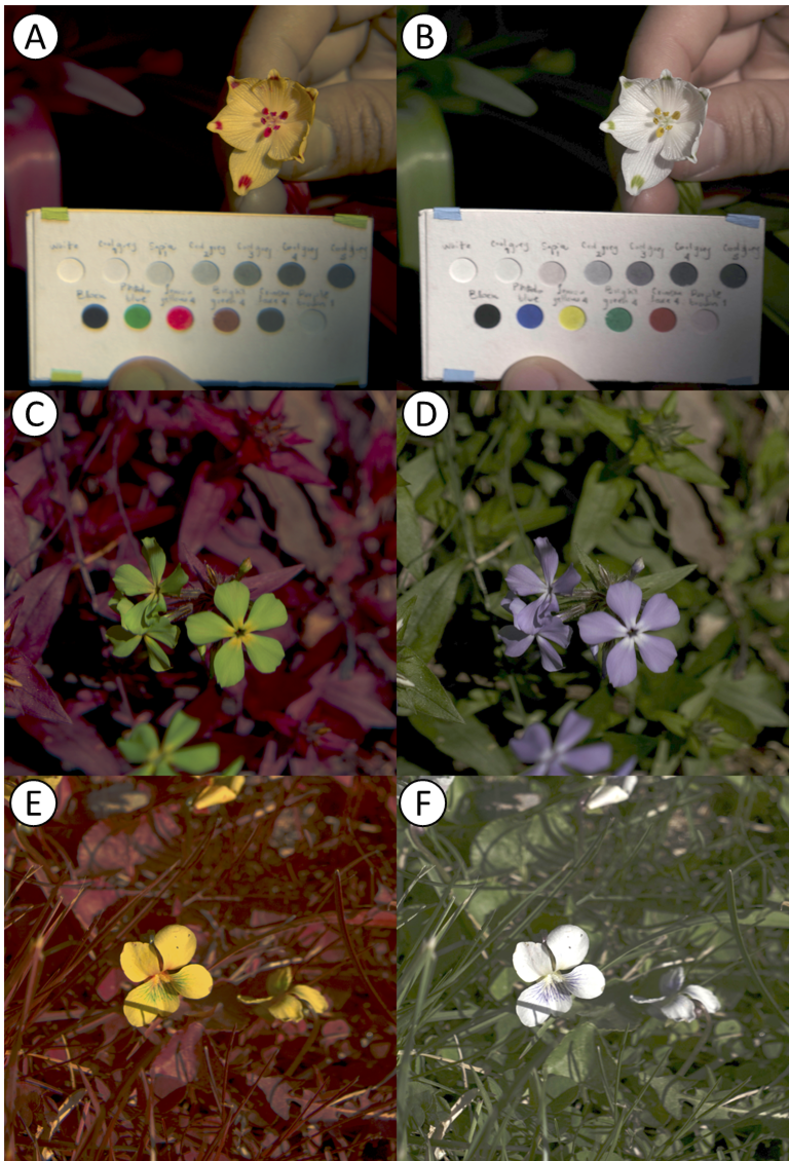

Supplement: S22 Fig — Here, we illustrate a (A, B) summer snowflake Leucojum aestivum, (C, D) blue phlox Phlox divaricata, and a (D, E) blue violet Viola sororia in honeybee false color (left) and human-visible colors (right). We also show a simple, cheap, pastel-based color standard that we used to validate animal-perceived quantum catches (A, B). We applied a gamma correction to the images (ACi0.3 and CCi0.5, respectively). (TIF) [file pbio.3002444.s050.tif]

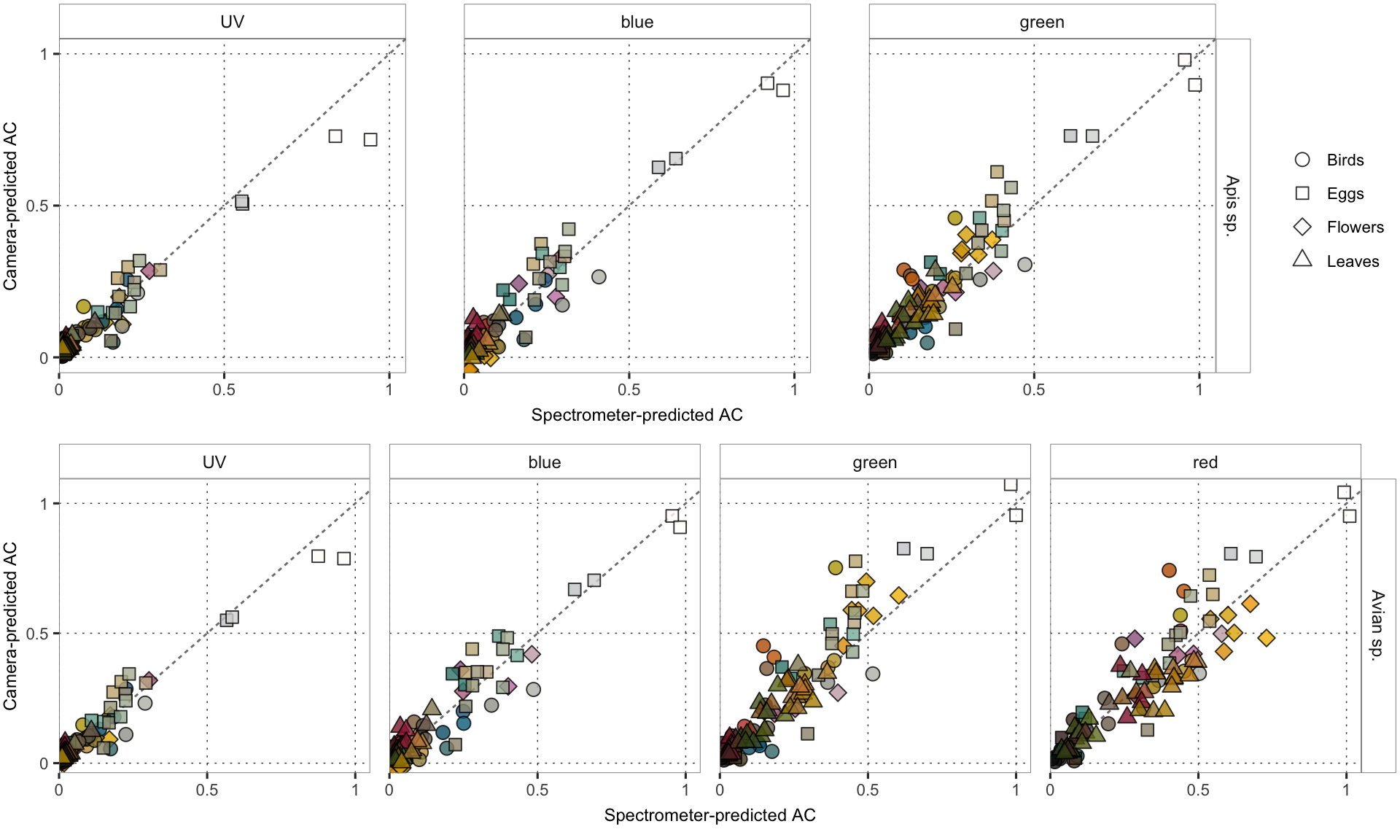

Supplement: S23 Fig — In this case, the images were taken under full sunlight and normalized to a set of ARUCO standards. The plots show the animal quantum catch predicted from reflectance (Spectrometer-predicted AC) against our camera-predicted animal quantum catch (Camera-predicted AC). We plot the fit for a collection of flowers (diamonds), leaves (triangles), birds’ eggs (squares), and birds’ feathers (circles); see S14 Table for sample details. The fit is shown for both the honeybee (Apis sp., top) and the average ultraviolet-sensitive avian receiver (Avian sp., bottom), for each of their 3 and 4 photoreceptors, respectively. The linear relationship between the 2 estimates was weaker for natural objects than for color standards. The mismatch represents a meaningful variation: the perceived color of natural objects is altered by their shape, fine patterning, texture, and physical color. The marker colors indicate the human-perceived color of the sample. For data on fit, please see S15 Table. The data underlying this figure can be found in S1 Data. (TIF) [file pbio.3002444.s051.tif]

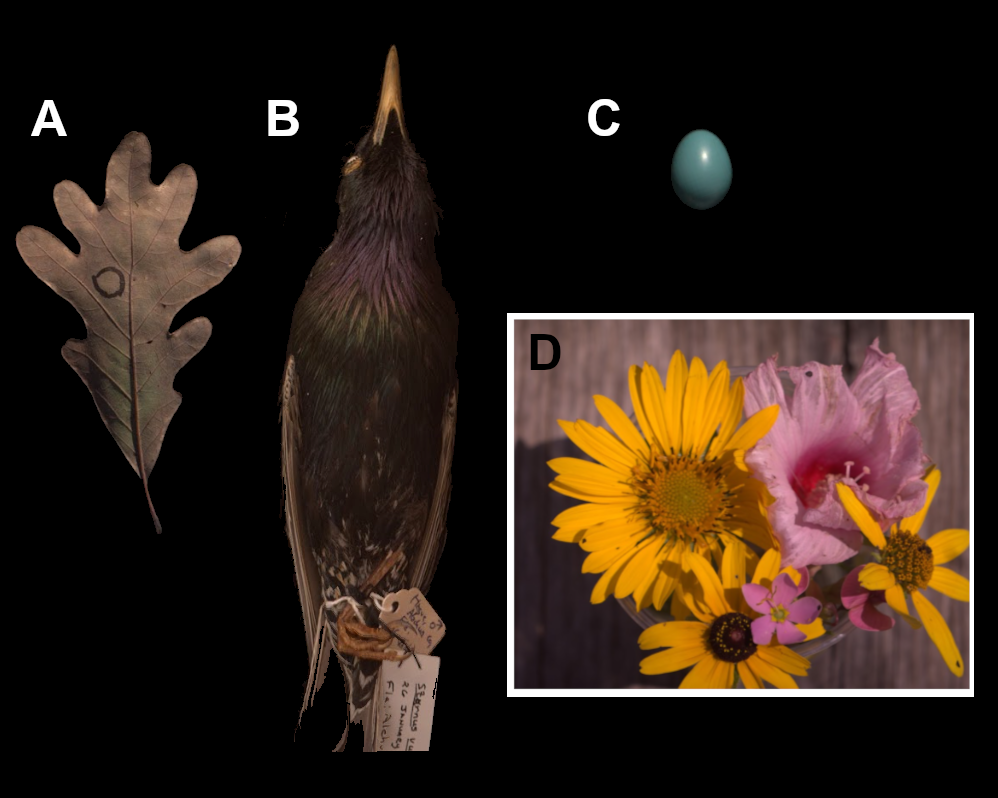

Supplement: S24 Fig — A display illustrating a subset of natural objects we used for assessing the accuracy of our system (S23 Fig and S14 and S15 Tables). We show (A) a white oak Quercus alba leaf, (B) a European starling Sturnus vulgaris specimen, (C) gray catbird Dumetella carolinensis egg, and (D) a bouquet of flowers. The correlation between spectrometry-predicted and camera-predicted animal catches is weaker for natural objects than for pastels (S23 Fig and S15 Table). The samples above illustrate the fact that the shape of stimuli, such as (A) leaves, (C) eggs, interacts with the direction of illuminating light and therefore impacts the appearance of surface colors. In some cases, such as (B) feather iridescence certain colors may (correctly) appear on an image but not on diffuse reflectance measurements. Finally, many objects such as flowers on (D) occlude and shade themselves and other plants. In all of these situations multispectral photography most likely provides a closer approximation to the quantum catches of free-living organisms than spectroscopy. (TIF) [file pbio.3002444.s052.tif]

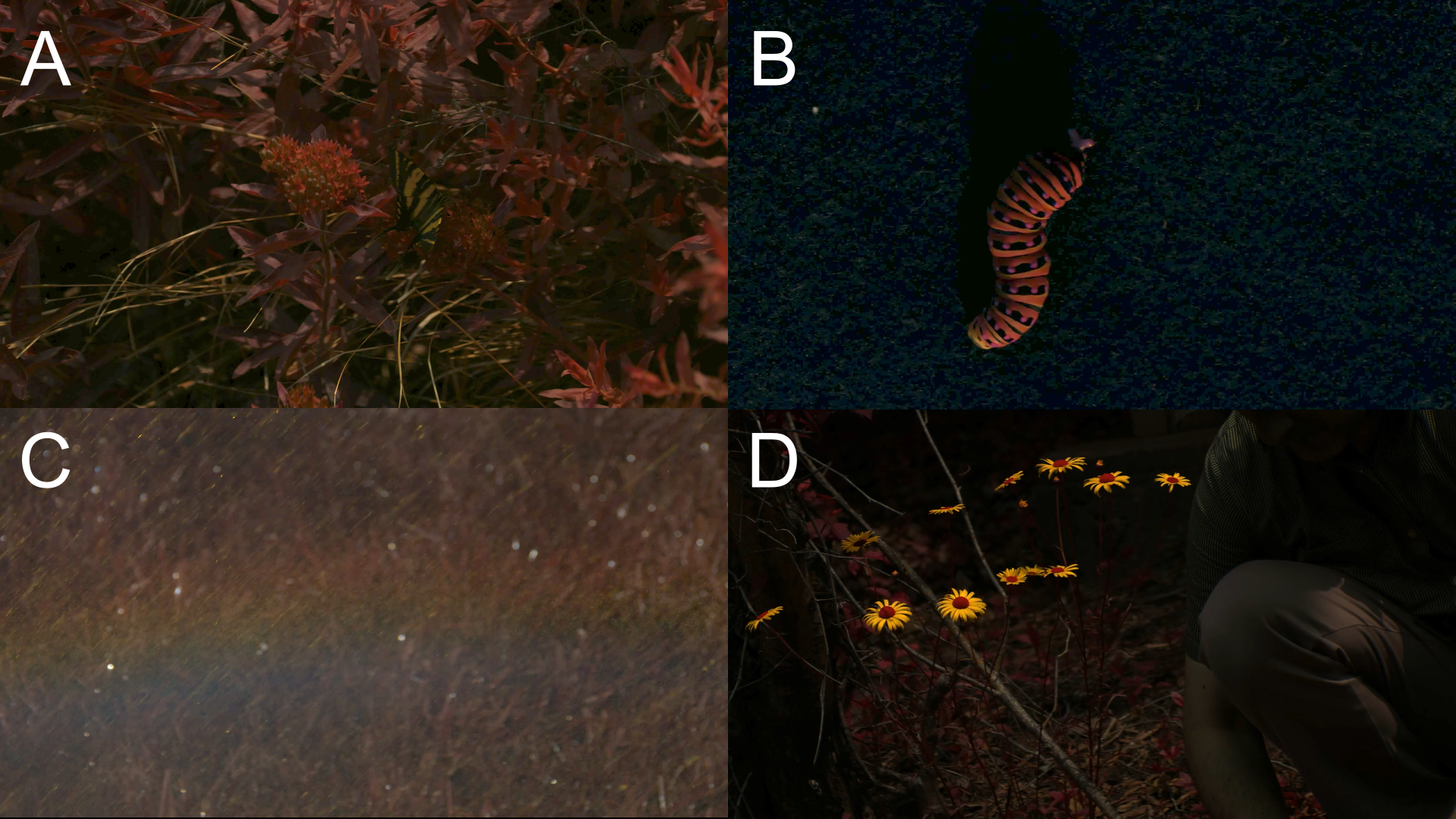

Supplement: S25 Fig — Our camera system provides a method that will allow researchers to accurately capture scenes in relative quantum catches units, in scenarios that would be challenging for other methods. For example, we illustrate a (A) zebra swallowtail butterfly Protographium marcellus moving between flowers, where assessing the color contrast between the butterfly and the background of flowers and leaves would be challenging to measure using spectroscopy. We show a (B) black swallowtail Papilio polyxenes caterpillar revealing its otherwise concealed osmeteria. This brief display would be impossible to measure using spectroscopy or traditional multispectral photography. We also illustrate a (C) rainbow, as an example of an optical effect that would be hard to accurately capture with any other method. Finally, we illustrate the (D) application of UV-blocking sunscreen, which would be measurable using other methods but not as a continuous movement. All frames are plotted in honeybee false color, where bee perceived colors are shifted into the human-visible space (i.e., UV, blue, and green quantum catch images are depicted as blue, green, and red in the false color image). The videos are available as S5–S8 Videos. (TIF) [file pbio.3002444.s053.tif]

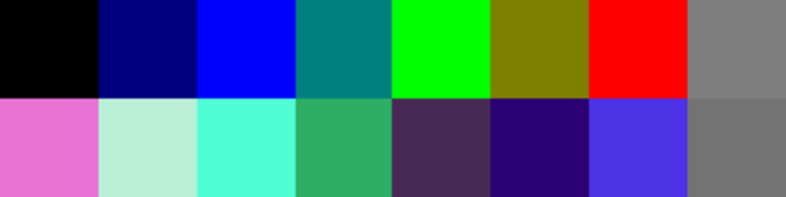

Supplement: S26 Fig — Left to right: ultraviolet, ultraviolet-blue, blue, blue-green, green, dark yellow, red, and gray. (TIF) [file pbio.3002444.s054.tif]
