# Supplementary material for: Recording animal-view videos of the natural world using a novel camera system and software package
Source: PLoS Biol. 2024 Jan 23;22(1):e3002444. doi: 10.1371/journal.pbio.3002444 (PMC10805291; doi:10.1371/journal.pbio.3002444)
Supplement: S1 Text — Extended information regarding the RNL color space (Method A), Spectroscopy (Method B), our approaches for image and video alignment (Method C), detailed information about SONY’s S-Log3 format (Method D), and tests of our system’s performance on narrowband reflectance (Method E). (DOCX) [file pbio.3002444.s055.docx]

S1 Text: Supplementary Information

**Recording animal-view videos of the natural world using a novel camera system and software package**

Table of contents:

Supplementary Methods Pages 2 - 13

Method A: RNL color space Page 2

Method B: Spectroscopy Page 5

Method C: Alignment Page 7

Method D: S-Log3 format Page 10

Method E: Performance on narrowband reflectance Page 12

References from the main text Page 13

References exclusively within S1 Text Supplementary information Page 13

**Supplementary Methods A: RNL Color Space**

Since humans are trichromatic, i.e. typically possess three different types of color receptors, visualizing videos from the perspective of animals that possess four or more receptor types is difficult. We adopt the Receptor Noise Limited color space from Vorobyev and Osorio [17], as presented in Renoult et al. [46], to provide a human-perceivable interpretation.

The RNL color space converts *N* color channels into *N* - 1 axes, with the transformation chosen so that the distance in the new color space is equal to the number of just-noticeable-differences between the colors. While the method can be applied to any number of color channels, we only use it for *N* = 4.

For a given pixel, let $Q_{i}$ denote the quantum catch of the band *i* photoreceptors. We apply a logarithmic transform to model the Weber-Fechner law:

$$s_{i}=log\left( Q_{i} \right)$$

To prevent numerical overflow, when $Q_{i}=0$ we clip it to an arbitrary minimum value $\hat{Q}=0.5 / 256$. We would ordinarily normalize by a background value; however, the application of the logarithm, followed by the differencing we will apply, eliminates the background term from the equations, so we omit this term.

We next apply the linear transformation:

$$X=c_{X}(s_{3}-s_{2})$$

$$Y= c_{Y}\left( s_{1}-{d^{Y}}_{1}s_{3}-{d^{Y}}_{2}s_{2} \right)$$

$$Z=c_{Z}\left( s_{0}-{d^{Z}}_{1}s_{1}-{d^{Z}}_{2}s_{2}-{d^{Z}}_{3}s_{3} \right)$$

where:

$$c_{X}=\frac{1}{\sqrt{{e_{2}}^{2}+{e_{3}}^{2}}}$$

$$c_{Y}=\sqrt{\frac{{e_{2}}^{2}+{e_{3}}^{2}}{{(e}_{2}e_{3})^{2}+(e_{1}e_{2})^{2}+(e_{1}e_{3})^{2}}}$$

$$c_{Z}=\sqrt{\frac{{(e}_{2}e_{3})^{2}+(e_{1}e_{2})^{2}+(e_{1}e_{3})^{2}}{(e_{1}e_{2}e_{3})^{2}+(e_{0}e_{2}e_{3})^{2}+(e_{0}e_{1}e_{3})^{2}+(e_{0}e_{1}e_{2})^{2}}}$$

$${d^{Y}}_{1}=\frac{{e_{2}}^{2}}{{e_{2}}^{2}+{e_{3}}^{2}}{, d^{Y}}_{2}=\frac{{e_{3}}^{2}}{{e_{2}}^{2}+{e_{3}}^{2}}$$

$${d^{Z}}_{1}=\frac{(e_{1}e_{2})^{2}}{{(e}_{2}e_{3})^{2}+(e_{1}e_{2})^{2}+(e_{1}e_{3})^{2}}$$

$${d^{Z}}_{2}=\frac{(e_{1}e_{3})^{2}}{{(e}_{2}e_{3})^{2}+(e_{1}e_{2})^{2}+(e_{1}e_{3})^{2}}$$

$${d^{Z}}_{3}=\frac{(e_{2}e_{3})^{2}}{{(e}_{2}e_{3})^{2}+(e_{1}e_{2})^{2}+(e_{1}e_{3})^{2}}$$

and

$$e_{i}=w\sqrt{\frac{\rho_{3}}{\rho_{i}}}$$

Here, *w* is the Weber fraction, assumed to be 0.1, and $\rho_{i}$ is the relative density of photoreceptors in band *i*. Note that each output, *X*, *Y*, and *Z*, is the difference between an input band and a weighted sum of the other bands, where the sum of the weights in each case is equal to 1. Since band 0 corresponds to ultraviolet in our avian photoreceptors, band 1 to blue, band 2 to green, and band 3 to red, we assign *X*, *Y*, and *Z* to the red, green, and blue channels respectively. This ensures that differences in longer wavelength quantum catches are expressed in the longer wavelength red channel, while differences in the shorter wavelength quantum catches are expressed in the shorter wavelength blue channel.

This transform produces outputs in a wide range, roughly [-40, 40]. We apply an affine transform to change the outputs to the range [0, 1], by calculating the minimum and maximum possible outputs across each band and using them to scale and shift *X*, *Y*, and *Z*. Since the inputs $Q_{i}$ are assumed to lie in the range [0, 1], the minimum and maximum possible values of $s_{i}$ are $log\hat{Q}$ (which is negative) and 0, where $\hat{Q}$ is our assumed minimum input value $(0.5 / 256)$. Since the sums of the weights in each channel are always 1, the minimum and maximum possible values of *X* are $c_{X}log\hat{Q}, c_{X}\left| log\hat{Q} \right|$, the minimum and maximum possible values of *Y* are $c_{Y}log\hat{Q}, c_{Y}\left| log\hat{Q} \right|$, and the minimum and maximum possible values of *Z* are $c_{Z}log\hat{Q}, c_{Z}\left| log\hat{Q} \right|$. In order to apply an equivalent transform to all channels, we take the minimum and maximum of these, obtaining:

$$\hat{X}=\frac{X-q_{-}}{q_{+}-q_{-}}, \hat{Y}=\frac{Y-q_{-}}{q_{+}-q_{-}}, \hat{Z}=\frac{Z-q_{-}}{q_{+}-q_{-}}$$

$$q_{+}=max(c_{X}, c_{Y}, c_{Z})\left| log\hat{Q} \right|$$

$$q_{-}=min(c_{X}, c_{Y}, c_{Z})log\hat{Q}$$

The ends of the range produced by this procedure occur when the colors in the input are maximally different, e.g. if band 0 has value 1.0 and all other bands have value 0. Since these extreme differences are relatively rare in nature, the resulting images tend to appear washed out. Further, very dark pixels, where all bands are close to zero, are mapped to the middle of the range, around 0.5. We therefore apply a final scaling, to map the brightness of the output pixels to match the brightness of the corresponding input pixel:

$\tilde{X}=\frac{|q|_{1}}{|\hat{X}|_{1}}\hat{X}$, $\tilde{Y}=\frac{|q|_{1}}{|\hat{Y}|_{1}}\hat{Y}$, $\tilde{Z}=\frac{|q|_{1}}{|\hat{Z}|_{1}}\hat{Z}$

Where $\left| \cdot\right|_{1}$ denotes the Manhattan distance.

When visualizing avian colors in the RNL color space, the colors are plotted by their coordinates in the RNL space (x = red, y = green, z = blue). Specifically, red pixel values represent the x axis, i.e. a greater response of avian photoreceptors sensitive to red light than those sensitive to green light, while absence of red means that the avian green intensity is greater than the avian red intensity. Similarly, a green pixel value (y axis) corresponds to the difference between the avian blue intensity and a weighted average of the avian red and green intensities, i.e. a higher green value implies that the avian blue photoreceptor is strongly excited. A blue pixel value (z axis) corresponds to the difference between the avian ultraviolet intensity and a weighted average of the other intensities, with high blue value implying a higher response of the ultraviolet receptors compared to all others. As the original RNL space includes negative values, the x, y and z axes are shifted to fit within the 0 to 255 range (see above). Consequently, bird-UV appears as pink; UV-blue as greenish-white; blue as turquoise; and so on. See S26 Fig for a set of example colors.

**Supplementary Methods B: Spectroscopy**

**Measuring transmission, reflectance, and sensitivity**

*Transmission and reflectance of optics*: The transmission spectra of lenses, bandpass filters, and the beam splitter (as well as the light reflected by the beam splitter) were measured relative to a broadband light source. Specifically, we delivered ultraviolet to near infrared light (240 nm to 1200 nm) from a xenon light source (Thorlabs, SLS 202) through a 1000 µm fiber optic (Edmund Optics, 58-458) into an integrating sphere (StellarNet IC2, effective range 200 nm to 1700 nm). Then, for lenses and bandpass filters, measurements were made with a spectrometer (Ocean Optics, Flame, effective range 280 nm to 1100 nm) coupled with a Spectralon-based cosine corrector (Ocean Optics, CC-3-DA). First, we measured a reference spectrum of the light from the integrating sphere before placing the optics in the beam path. To measure the reflectance and transmission of the dichroic beam splitter we placed it in a dichroic filter mount (Thorlabs, CM1-DCH – 30), equipped with fiber mounts (Thorlabs SM1SMA) housing two 1000 µm fiber optics (Edmund Optics, 58-458) that passed reflected and transmitted light. This light was in turn directed to the spectrometer through a 1000 µm fiber optic (Edmund Optics, 58-458). Again, we measured a reference spectrum prior to placing the optics within the beam path. To reduce stray light, we capped (Thorlabs, SM1CP2) the opening opposite of the reflection port of the dichroic filter mount and placed its open front port directly in front of the fully illuminated integrating sphere.

*Reflectance of objects*: We measured diffuse spectral reflectance using a field-portable spectrophotometer (Jaz, Ocean Optics) with an internal pulsed xenon (Jaz-PX, Ocean Optics) light source. All measurements were relative to an isoluminant white standard (WS-1-SL, Ocean Optics) that was made from Spectralon and reflected 99% of incident radiation over large spans of the electromagnetic spectrum (~200-2300 nm). We used a custom black box that was coated with spectrally dark paint as a dark standard (Culture Hustle, Black 3.0, see lowest spectrum on S14 Fig). We used a 600 µm bi-furcating fiber optic and to avoid glare, we measured at a coincident oblique measurement angle.

*Sensitivity*: To ensure that our camera system didn’t pass significant quantities of infrared light, we remeasured the entire system’s sensor sensitivities up to ~1100 nm. This required a slightly different approach than the sensitivity measurements described in the main text. For these measurements, we followed the procedure from the manuscript, but this time used two spectrometers, an Ocean Optics Flame and an Ocean Optics NIRQuest, that were sensitive from 300 nm to 1100 nm and 900 nm to 1700 nm, respectively. In practice, we found that the Flame spectrometer became unreliable at ~950 - 1000 nm. Both had a Spectralon-based cosine corrector (Ocean Optics, CC-3-DA) mounted over the measurement port, as described in the main manuscript, and both were radiometrically calibrated to a NIST calibrated 200-Watt quartz tungsten-halogen lamp (Optronic Laboratories, OL 220C). To measure spectral sensitivities, we adjusted light from a xenon light source (Thorlabs, SLS 202) using monochromators. In this case, we need to use two monochromators to cover the extended range (Optimetrics, DMC1-03 and DMC1-04), that were effective from 300 nm to 800 nm and from 500 nm to 1200 nm. Broadband light was delivered to the monochromator using a 1000 µm fiber optic (Edmund Optics, 58-458). Then, light from the monochromator was shone directly at the cosine correctors of each spectrometer using a second 1000 µm fiber optic (Edmund Optics, 58-458) with a mounted collimating lens (Ocean Optics, 74-UV). Finally, we incrementally adjusted the monochromator from 280 nm to 1100 nm and at each increment we photographed the illuminated cosine corrector and saved a spectrum. For each increment, we adjusted the shutter speed of the camera and the integration time of the spectrometer, as necessary. We also recorded a dark spectrum and dark photograph at each respective increment. This time we removed second order scatter by using inline longpass filters (Semrock BLP01-532R-25: 540 nm to 700 nm, and MidOpt LP715: 715 nm to 1100 nm). The RAW average pixel for each dark image was again subtracted by the pixel value for each RAW photograph of the illuminated cosine corrector; however, this time we used the pixel value per second. Then, the resultant signal was then divided by the irradiance (µWatt cm^-2^), within 40 nm of each peak (see equation 1 in main text). We have not found measurable responses in the 700 nm -1100 nm range (S12 Fig).

**Supplementary Methods C: Alignment**

**Homography Warp**

Our image processing pipeline involves a two-stage process of aligning the VIS and UV cameras. First, we apply a coarse alignment of the UV channel using a homography warp. Homography warp is widely used in computer vision for changing the perspective of images. We provide only a brief description here, based on the implementation from Bradski [66].

The homography warp is a method of transforming an image to approximate the effect of flips, rotations, and other perspective shifts. The warp is defined by eight coefficients $C_{1}, C_{2}, C_{3}, C_{4}, C_{5,} C_{6,} C_{7,} C_{8}$, typically abbreviated as a matrix $C$ whose lower-right coefficient is 1. For a point $p=(x, y)$, we calculate the coordinates of the warped point $p'=(x', y')$ by:

$$x'=\frac{C_{1}x+C_{2}y+C_{3}}{C_{7}x+C_{8}y+1}$$

$$y'=\frac{C_{4}x+C_{5}y+C_{6}}{C_{7}x+C_{8}y+1}$$

Homography warps can be inverted by finding the inverse of the matrix $C$. When applying a homography warp to an image, the warp is inverted and the coordinates of each pixel in the resulting image are located in the original image. The values for each pixel are then determined by interpolation, typically bilinear.

The warp coefficients are determined by manually selecting matching points on the UV and VIS images. Since the cameras are fixed in position, the coarse alignment can be found once and then reused.

**Enhanced Correlation Coefficient (ECC) Method**

The second step of the alignment process is to apply a fine-grained correction the homography warp coefficients identified during the coarse alignment. We used the Enhanced Correlation Coefficient (ECC) algorithm for refining the coefficients. Here we provide a brief description of the ECC method [53].

The ECC algorithm aligns a warped image to a reference image of the same scene. The method is computationally efficient and reasonably invariant to photometric distortions. Its main disadvantage is that, since it uses gradient descent to find the alignment, it requires a good initial coarse alignment to avoid becoming trapped in a local minimum.

We begin by converting each image to a single band by taking the mean across the bands. Let us define $i_{r}$ to be the reference image (e.g., the greyscale image from the visual camera) and define $i_{w}(p)$ to be the warped image (e.g., the greyscale image from the ultraviolet camera) following a homography warp $p$. We interpret each image as a vector in some high-dimensional space, where each entry corresponds to a pixel. We define the ECC value to be:

$$ECC(p)=\left| \left| \frac{\underline{i}_{r}}{\left| \left| \underline{i}_{r} \right| \right|}-\frac{\underline{i}_{w}(p)}{\left| \left| \underline{i}_{w}(p) \right| \right|} \right| \right|^{2}$$

Where $\underline{i}_{r}, \underline{i}_{w}(p)$ refer to the de-meaned vectors obtained by subtracting the mean and $\left| \left| \cdot\right| \right|$ is the Euclidean norm. This is a target objective that will be zero if the normalized images are equal. Minimizing the ECC is equivalent to maximizing the quantity:

$$\rho(p)=\frac{{\underline{i}_{r}}^{T}\underline{i}_{w}(p)}{\left| \left| \underline{i}_{r} \right| \right|\left| \left| \underline{i}_{w}(p) \right| \right|}$$

Where the superscript T denotes the transpose. Applying a first order Taylor approximation, the arbitrary quantity $\rho$ can be minimized by steps:

$\Delta p=(\underline{G}^{T}\underline{G})^{-1}\underline{G}^{T}\left( \frac{\left| \left| \underline{i}_{w}(p) \right| \right|^{2}-{\underline{i}_{w}}^{T}(p)P_{G}\underline{i}_{w}(p)}{{\underline{i}_{r}}^{T}\underline{i}_{w}(p)-{\underline{i}_{r}}^{T}P_{G}\underline{i}_{w}(p)} \right)$ if ${\underline{i}_{r}}^{T}\underline{i}_{w}(p)>{\underline{i}_{r}}^{T}P_{G}\underline{i}_{w}(p)$

$\Delta p=(\underline{G}^{T}\underline{G})^{-1}\underline{G}^{T}\left( \lambda\frac{\underline{i}_{r}}{\left| \left| \underline{i}_{r} \right| \right|}-\underline{i}_{w}(p) \right)$ otherwise

Where $G$ is the Jacobian matrix of the warped image $\underline{i}_{w}(p)$ in terms of $p$, $\underline{G}$ is the column-de-meaned $G$, and:

$$P_{G}=\underline{G}(\underline{G}^{T}\underline{G})^{-1}\underline{G}^{T}$$

$\lambda=$ max$\left( \sqrt{\frac{\left( {\underline{i}_{w}}^{T}(p)P_{G}\underline{i}_{w} \right)\left| \left| \underline{i}_{r} \right| \right|^{2}}{{\underline{i}_{r}}^{T}P_{G}\underline{i}_{r}}, \frac{\left( {\underline{i}_{r}}^{T}P_{G}\underline{i}_{w}(p)-{\underline{i}_{r}}^{T}\underline{i}_{w}(p) \right)\left| \left| \underline{i}_{r} \right| \right|}{{\underline{i}_{r}}^{T}P_{G}\underline{i}_{r}}} \right)$

Under this procedure, the ECC decreases monotonically until reaching a minimum.

We incorporate several minor improvements on the technique, using the implementation in OpenCV [66]. First, in all operations preceding the calculation of the ECC, we calculate a mask of the valid pixels. We masked out a 50-pixel margin around the border of each image. Additionally, during each affine warp we track which pixels are filled in by the warp and mask out null pixels. For example, during the coarse alignment homography warp, we consider the black space around the edge of the image after the warp to be invalid. We then calculate the ECC only over the valid pixels.

Second, we calculate multiple ECC warps using different frames, typically four. We then calculate the ECC of all of the VIS frames and the warped UV frames in the batch for each warp, and select the warp that results in the lowest mean ECC across frames.

**Temporal Synchronization Method**

When performing temporal synchronization we first locate a batch of images that contain significant motion. We detect motion by first calculating a reference background from the batch. Each batch generally consists of 32 frames, but the batch size is limited by available memory and may need to be reduced on some computers. If the batch contains at least eight frames, we calculate the background by taking the median value of each pixel across the batch. If the batch contains fewer than eight frames, we found the median to be less stable, and use the mean instead.

Once the background is determined, we calculate the absolute value of the difference between each pixel in the frame and the corresponding pixel in the background. We declare a pixel to be in motion if the difference exceeds 0.1, where the pixel values have been scaled to a [0.0, 1.0] range. We declare the frame to be in motion if at least 0.5% of the pixels in the frame are in motion. We process batches until we find a batch that contains motion.

Once we have found a batch of frames that contain motion, we perform a spatial alignment for each possible temporal shift within a range of shifts, generally −10 frames to +10 frames. Videos that are more than 10 frames apart need to be manually cropped before processing. For each possible temporal shift, we use the calculated spatial alignment to align the batch of frames, then calculate the average ECC value across each pair of aligned and shifted UV and VIS frames in the batch. We select the temporal shift and spatial alignment that produces the best average ECC value.

**Supplementary Methods D: S-Log3 format**

**Derivation of S-Log3 Formula**

The formula for the conversion of JPG pixel values to linear, RAW pixel values (provided in equation 2 in main text) is derived from the formulae provided by Sony Corporation [51]:

$r=10^{(x_{\mathrm{JPG}}\cdot1023.0-420)/261.5}\cdot(0.18+0.01)-0.01$ for $x_{\mathrm{JPG}}\geq171.2102946929/1023$

and

$\frac{0.01125(x_{\mathrm{JPG}}\cdot1023.0-95.0)}{171.2102946929-95.0}$ for $x_{\mathrm{JPG}}<171.2102946929/1023$

The constant $c_{4}=171.2102946929/1023=0.1673609$.

First, we derive the top equation in equation 2 (see main text) as follows:

$$r=10^{(x_{J\mathrm{PG}}\cdot1023.0-420)/261.5}\cdot\left( 0.18+0.01 \right)-0.01=$$

$$=0.19\cdot10^{\left( \frac{1023}{261.5} \right)\cdot x_{\mathrm{JPG}}-\left( \frac{420}{261.5} \right)}-0.01=$$

$$=0.19\cdot10^{-\frac{420}{261.5}}10^{\left( \frac{1023}{261.5} \right)\cdot x_{\mathrm{JPG}}}-0.01=$$

$$=0.19\cdot10^{-\frac{420}{261.5}}\left( 10^{\frac{1023}{261.5}} \right)^{x_{\mathrm{JPG}}}-0.01=$$

$$=c_{1}{c_{2}}^{x_{\mathrm{JPG}}}+c_{3}$$

where:

$$c_{1}=0.19\cdot10^{-420/261.5}=0.0047058$$

$c_{2}=$ $10^{1023/261.5}$ = $8166.69$

$$c_{3}=-0.01$$

The lower equation in equation 2 (see main text) is obtained by:

$$\frac{0.01125(x_{\mathrm{JPG}}\cdot1023.0-95.0)}{171.2102946929-95.0}=$$

$$=\left( \frac{0.01125\cdot1023.0}{171.2102946929-95.0} \right)x_{\mathrm{JPG}}-\left( \frac{0.01125\cdot95.0}{171.2102946929-95.0} \right)=$$

$$=0.14101306x_{\mathrm{JPG}}-0.014023696$$

In practice, we found that introducing an additional scaling term improved the accuracy:

$r=c_{1}{c_{2}}^{sx_{\mathrm{JPG}}}+c_{3}$ for $sx_{\mathrm{JPG}}\geq c_{4}$

$r=c_{5}{sx}_{\mathrm{JPG}}+c_{6}$ for ${sx}_{\mathrm{JPG}}<c_{4}$

where the scaling term for the input is $s=0.92578125$.

To confirm that these linearized images are adequate representations of RAW images, we tested the formulae on a library of 84 still images for which we had both RAW and JPG versions. The images were taken under a variety of lighting conditions: indoors and outdoors. We replaced the reflectance $r$ with $m_{b}x_{\mathrm{ARW}}$, where $x_{\mathrm{ARW}}$ is the pixel value in the RAW image and $m_{b}$ is a band-specific scaling factor calculated by using the least-squares to fit $r$ calculated from the JPG. The mean absolute error associated with conversions from JPG to RAW were exceedingly small (S16 Table). We compared this error to those resulting from converting RAW to JPG and then back to RAW (i.e., “roundtrip”), essentially quantifying the data loss resulting from compression to JPG format. Since the RAW to JPG conversion should be the only lossy step, if the process of reconstructing the RAW is accurate, then the RAW to JPG error and the roundtrip error should be comparable. Factoring in losses due to 8-bit quantization and the lossy compression of the JPG algorithm, the mean absolute error of JPG to ARW and roundtrip conversions were comparable across all color channels.

**Supplementary Methods E: Performance on Narrowband Reflectance**

Our method relies on the fact that real reflectance spectra of natural materials tend to be relatively broadband. To evaluate the reliability of quantum catch estimates when imaging objects with narrowband reflectance spectra, we estimated the accuracy using idealized narrowband curves. In this analysis, we assumed that reflectance follows a Gaussian curve, and generated spectra with varying maximum reflectance peak values and full-width-at-half-maximum (FWHM) values. We used animal photoreceptor sensitivities described by a standard photoreceptor template with a specified peak sensitivity as in Govardovskii et al. [59], assumed ideal illumination and trained the conversion map using the FReD database [58]. We calculated the absolute error in the transformation step, and plotted these errors as a heatmap (S21 Fig).

As expected, the largest errors occur when the reflectance curve is narrowest (FWHM=10 nm). Even in this case, however, the highest absolute error is still less than 0.017. This is comparable to the highest mean absolute error when testing the conversion for the photoreceptor templates on the test portion of the FReD, and about four times as high as the highest mean absolute error when testing the conversion for avian and *Apis* photoreceptors. In other words, while the error is higher than the typical use cases we present, it remains within acceptable ranges, even for the narrowest curves. For context, LED lights that are used as narrow band points sources have spectra with FWHM values ranging from ~3-7 nm to 150 - 200 nm [69].

**References from the main text**

17. Vorobyev M, Osorio D. Receptor noise as a determinant of colour thresholds. Proc R Soc London B. 1998;265: 351–358. doi:10.1098/rspb.1998.0302

46. Renoult JP, Kelber A, Schaefer HM. Colour spaces in ecology and evolutionary biology. Biol Rev. 2017;92: 292–315. doi:10.1111/brv.12230

51. Sony Corporation. Technical Summary for S-Gamut3.Cine/S-Log3 and S-Gamut3/S-Log3. 2016. pp. 1–7. Available: https://pro.sony/s3/cms-static-content/uploadfile/06/1237494271406.pdf

53. Evangelidis G, Psarakis E. Parametric image alignment using enhanced correlation coefficient maximization. IEEE Trans Pattern Anal Mach Intell. 2008;30: 1858–1865. Available: https://hal.inria.fr/hal-00864385

58. Arnold SEJ, Faruq S, Savolainen V, McOwan PW, Chittka L. FReD: The floral reflectance database - a web portal for analyses of flower colour. PLoS One. 2010;5: 1–9. doi:10.1371/journal.pone.0014287

59. Govardovskii VI, Fyhrquist N, Reuter T, Kuzmin DG, Donner K. In search of the visual pigment template. Vis Neurosci. 2000;17: 509–528.

66. Bradski G. The OpenCV library. Dr Dobb’s J Softw Tools. 2000;25: 120–125.

**References exclusively within Supplementary information**

69. Galyanin V, Belikov V, Belikova V, Bogomolov A. Emission band width approximation of light-emitting diodes in the region 350–2100 nm. Sensors Actuators, B Chem. 2017;252: 773–776. doi:10.1016/j.snb.2017.05.119
